# Global, regional, and national burden of ischemic heart disease and ischemic stroke and their risk factors in youths and young adults aged 15–39 years (1990–2021): a comparative analysis of risk factors from global burden of disease study 2021

**DOI:** 10.1080/16549716.2025.2560711

**Published:** 2025-10-16

**Authors:** Siwei Xie, Long Zhang, Yan Zhang, Zhi-Jie Zheng, Jianping Li, Shuduo Zhou

**Affiliations:** a Department of Clinical Epidemiology, Peking University First Hospital, Beijing, China; b Department of Environmental Health and Engineering, Johns Hopkins Bloomberg School of Public Health, Baltimore, MD, USA; c Department of Cardiology, Peking University First Hospital, Beijing, China; d Department of Global Health, Peking University School of Public Health, Beijing, China

**Keywords:** Ischemic heart disease, ischemic stroke, youths and young adults, disease burden, disease disparities, comparative analysis

## Abstract

**Background:**

There has been a growing trend of ischemic heart disease (IHD) and ischemic stroke (IS) affecting younger populations worldwide.

**Objectives:**

Assess and compare disease burden of IHD and IS in youth and young adults aged 15–39 from 1990 to 2021.

**Methods:**

Using data from the Global Burden of Disease (GBD) 2021 study for individuals aged 15–39 years across 204 countries and territories, we estimated age-standardized incidence, prevalence, mortality rate, and disability-adjusted life years (DALYs) for IHD and IS. Temporal trends from 1990 to 2021 were evaluated using the average annual percentage change (AAPC). We estimated risk factor contributions by calculating the population attributable fractions (PAFs) of DALYs.

**Results:**

In 2021, the global age-standardized incidence rate (per 100,000 population) of IHD among youth and young adults was 36.86, approximately three times higher than that of IS (12.84). From 1990 to 2021, incidence and prevalence of IHD increased significantly (AAPC 0.25% and 0.20%). IS showed declining trends in all metrics (*p* < .001), but experienced a resurgence in incidence after 2015. In 2021, males had higher IHD incidence rates than females (44.33 vs 29.24), while females had higher IS incidence rates than males (13.47 vs 12.23). Dietary risks are the leading contributors to IHD DALYs globally (66.2% in 2021).

**Conclusions:**

This study highlights significant global disparities in the burden and trends of IHD and IS among young adults. More comprehensive and proactive interventions are urgently needed across all countries to mitigate the burden of IHD and IS on the young population.

## Paper Context

- **Main findings**: This study reveals rising trends in ischemic heart disease among youths and young adults worldwide, while ischemic stroke showed initial declines until 2015 but has since resurged in several regions, with significant differences between sexes and regions.
- **Added knowledge**: This research provides the comprehensive global comparative analysis to date of ischemic heart disease and ischemic stroke burden and risk factors among youths and young adults using the global data.
- **Global health impact for policy and action**: The findings highlight the urgent need for targeted prevention strategies, lifestyle interventions, and improved healthcare access to reduce cardiovascular diseases in young populations worldwide.

## Background

Ischemic heart disease (IHD) and ischemic stroke (IS) are among the leading causes of morbidity and mortality worldwide, significantly contributing to the global burden of cardiovascular diseases [1–3]. Driven by factors such as unhealthy lifestyles, rising metabolic diseases, mental stress, and environmental pollution, recent trends have shown a concerning rise in the incidence and prevalence of IHD among youth and young adults aged 15 to 39 years. Over the past three decades, the global incidence and prevalence rates of IHD in this age group have increased by approximately 16% and 19%, respectively, with even higher increases observed in low- and middle-income countries [4–7]. This shift represents a significant public health challenge, as the early onset of cardiovascular disease may contribute to prolonged disability, reduced quality of life, and sustained increases in healthcare costs across the lifespan [8–10]. Beyond individual health consequences, the early onset of IHD and IS among young adults imposes considerable economic and societal burdens, including productivity losses, long-term disability care, and increased demands on healthcare systems.

Comparing IHD and IS in the young population is essential because these conditions share common etiological pathways and risk factors, including hypertension, smoking, and unhealthy diets. At the same time, each disease also involves distinct pathophysiological mechanisms and clinical trajectories, which makes a comparative perspective important [5,6,9,11]. Despite shared risk factors, IHD and IS may display distinct patterns in incidence, severity, progression, or outcomes among youth and young adults [11–13]. The dual perspective of analyzing IHD and IS together also helps avoid misleading conclusions that may arise if the conditions are studied in isolation. Conducting a comparative analysis simultaneously allows for the identification of both shared and distinct epidemiological patterns and risk profiles, enhancing our understanding of common pathophysiological pathways and unique disease mechanisms. This approach allows the identification of regional variations, sex-related disparities, and associated risk factors, thereby informing the development of integrated prevention strategies targeting shared modifiable risks. Simultaneously, it supports the development of tailored interventions that address the unique features of each disease.

Currently, there is a significant gap in the literature regarding the comparative analysis of IHD and IS trends in the young population. Many existing studies have either focused on the general population or examined both IHD and IS separately, leaving a critical gap in understanding their comparative patterns among youth and young adults [5,6,10,14,15]. For instance, Zhang et al. and Yuan et al. investigated young-onset IS and IHD separately [5,6], while Shi et al. projected IHD burden but did not specifically target younger populations [10]. Earlier studies such as Feigin et al. and Boot et al. provided valuable insights into stroke burden, but their data are relatively outdated or limited in scope [14,15]. To our knowledge, a comparative analysis of the epidemiological characteristics and risk factors for IHD and IS in this age group has not been well documented on a global scale using the updated GBD data.

To address this gap, this study utilized data from the Global Burden of Disease (GBD) study (1990–2021) to systematically analyze age-standardized incidence, prevalence, mortality, and disability-adjusted life years (DALYs) attributable to IHD and IS among individuals aged 15–39 years. By examining temporal trends, regional disparities, sex differences, and key risk factors, this study aimed to enhance understanding of the evolving epidemiology of IHD and IS in this young population. The findings are intended to support policymakers and healthcare providers in developing proactive and age-specific public health interventions to mitigate the global burden of IHD and IS.

## Methods

### Study population

The GBD 2021 provided comprehensive estimates of incidence, prevalence, mortality, and DALYs for IHD and IS, stratified by age and sex, across 204 countries and territories from 1990 to 2021. The modeling was based on 1000 iterations, with 95% uncertainty intervals (UIs) derived from the 2.5th-to 97.5 th-ranked values. Our analysis focused on IHD and IS among individuals aged 15–39 years, categorized as youths (15–19 years) and young adults (20–39 years), consistent with previous GBD-based publications [4,5,16]. We extracted data for five age-specific groups: 15–19, 20–24, 25–29, 30–34, and 35–39 years. For regional analyses, we selected the socio-demographic index (SDI) (high, high-middle, middle, low-middle, and low SDI), World Bank regions (East Asia & Pacific, Europe & Central Asia, Latin America & Caribbean, Middle East & North Africa, North America, South Asia, and sub-Saharan Africa), World Bank income levels (high, upper middle, lower middle, and low income), and health systems classifications (advanced, basic, limited, and minimal). All regional data were obtained using the GBD Results Tool (https://vizhub.healthdata.org/gbd-results/).

The SDI is a composite indicator that reflects a country’s level of social and economic development and is closely linked to population health outcomes [17]. It is based on three components: income per capita, educational attainment, and fertility under age 25. It ranges from 0 to 1, with higher values indicating higher levels of socioeconomic and health-related development. Countries and territories were grouped into quintiles according to SDI. Detailed descriptions of GBD methodologies and studies are available in previous publications [18].

### Outcomes

This study has three main outcomes: (1) age-standardized incidence, prevalence, mortality, and DALYs per 100,000 youth and young adults from 1990 to 2021. (2) Average annual percentage change (AAPC) for all metrics from 1990 to 2021. (3) Age-standardized proportions of DALYs attributable to risk factors.

### IHD and IS disease burden estimation framework of young population in GBD 2021

To estimate the disease burden of IHD and IS among young adults, we used a framework consistent with GBD methodologies. The incidence and prevalence estimate of IHD and IS were generated using the publicly available DisMod-MR 2.1 software, a Bayesian meta-regression modeling tool accessible via the GBD Results Tool [18]. This modeling approach synthesizes and standardizes data from diverse sources, including population surveys, cohort studies, health system administrative records, and disease registries, to provide comprehensive epidemiological estimates. DisMod-MR 2.1 addresses data heterogeneity by borrowing strength across geographies and time, applying covariates, and propagating uncertainty, which helps mitigate issues of limited or inconsistent data in low-resource settings.

Mortality rates were estimated using the cause-of-death ensemble model (CODEm), which systematically combines multiple cause-of-death estimation methods into an ensemble to optimize predictive validity. This model uses the GBD cause of death database, which integrates various data sources, including vital registration with correction of under-registration and garbage coding, verbal autopsy at national and subnational levels with correction of garbage coding, surveillance systems for specific causes such as maternal mortality, and other epidemiological studies. By adjusting for under-reporting, misclassification, and other biases, CODEm improves comparability of mortality estimates across countries, particularly where registration systems are incomplete. We classified mortality data based on the International Classification of Diseases (ICD) codes [19]. DALYs were calculated as the sum of years of life lost due to premature mortality and years lived with disability, with adjustments made for comorbidities [7]. This study followed the Guidelines for Accurate and Transparent Health Estimates Reporting (GATHER) [18].

### Attributable burden of IHD and IS due to risk factors

The risk framework in the GBD Comparative Risk Assessment (CRA) classifies risk factors into four levels. To evaluate the attributable burden of IHD or IS related to the available risk factors in GBD 2021 (including environmental/occupational risks at level 1, metabolic risks at level 1, air pollution, tobacco, dietary risks at level 2, and 27 specific risk factors for IHD and 23 for IS), we calculated the population attributable fractions (PAFs) of DALYs [20]. The difference in the number of risk factors was due to the GBD framework, which includes only those risk – outcome pairs with sufficient evidence of an associated or causal relationship. Thus, fewer risk factors are currently defined for IS than for IHD. And since the percent or number of DALYs are not mutually exclusive, the total PAFs of risk factors may exceed 100%. This is because many of these risk factors have overlapping effects, with some being mediated partially or entirely through other risk factors [21]. The specific risk factors analyzed included: (1) environmental/occupational risks and air pollution: ambient particulate matter pollution; household air pollution from solid fuels; lead exposure; low temperature; high temperature; (2) dietary risks: diet low in whole grains; diet low in fruits; diet low in fiber; diet low in vegetables; diet low in legumes (only for IHD); diet high in sodium; diet high in red meat; diet high in processed meat; diet low in nuts and seeds (only for IHD); diet high in trans fatty acids (only for IHD); diet high in sugar-sweetened beverages; diet low in seafood omega-3 fatty acids (only for IHD); diet low in omega-6 polyunsaturated fatty acids; (3) other behavioral risks: low physical activity; alcohol use; (4) tobacco: smoking; secondhand smoking; (5) metabolic risks: high fasting plasma glucose; high LDL cholesterol; high systolic blood pressure; high body-mass index (BMI); and kidney dysfunction. Definitions of the included risk factors are provided in Supplementary Table S1 [22]. The methodologies used in GBD 2021 for estimating IHD and IS burden and associated risk factors are consistent with those of the most recent GBD studies and have been detailed in other publications [22–24].

### Statistical analysis

Age-standardized rates for youth and young adults were calculated using the GBD 2021 global population as the reference, ensuring that differences in age structures did not affect comparisons between countries or regions. Specifically, for the age range of 15 to 39 years, we extracted age-specific weights from the GBD 2021 global reference population for each five-year age interval (15–19, 20–24, 25–29, 30–34, and 35–39 years). These original weights were then proportionally adjusted so that their total summed to 100%, representing only the targeted age groups. Each group’s incidence, prevalence, mortality, and DALYs rate were multiplied by its adjusted weight, and these products were then summed across all age groups within this range, standardizing the rate specifically to this 15–39 age group. We calculated the AAPC along with its 95% confidence interval (CI) for all metrics from 1990 to 2021 to assess the magnitude and direction of the temporal trends. The Joinpoint Regression Program (version 4.9.0.0, National Cancer Institute, USA) was employed to perform joinpoint regression analysis [25,26]. A Monte Carlo permutation method was used to determine the statistical significance of the trend changes. An AAPC greater than zero with a p-value less than 0.05, it indicated a significantly increasing age-standardized trend, whereas an AAPC less than zero with a p-value less than 0.05 indicated a significantly decreasing trend. If the p-value was greater than or equal to 0.05, the trend was considered to be stable. The AAPC was chosen for its ability to capture both linear and non-linear trends, providing a comprehensive summary of the changes throughout the entire study period. This approach facilitated the identification of segments with significant trend shifts, as represented by joinpoints. The Annual Percentage Change (APC) was computed for each segment between joinpoints, and the AAPC was derived by averaging the APCs, with weighting based on the duration of each segment to ensure that longer periods had greater influence. Additionally, the proportional contribution of each risk factor was determined by dividing the age-standardized DALYs rate attributable to a specific risk factor by the total age-standardized DALYs rate for IHD and IS.

All non-specific software analyses were conducted using R software (version 4.3.2, R Project for Statistical Computing), with statistical significance defined as a two-sided *p*-value of < 0.05.

## Results

In 2021, the global age-standardized incidence rate for youth and young adults (ages 15–39) of IHD was 36.86 per 100,000 population (95% UI 19.50–58.52), about three times higher than that of IS (12.84 per 100,000 [95% UI 7.29–20.57]) (Table 1). Age-standardized mortality rates were also significantly higher in the IHD group (6.73 vs 0.51 IS). The age-standardized DALYs rates were 394.56 for IHD versus 59.76 and IS. However, the prevalence rate was higher for IS (205.24 [178.00–233.75]) than for IHD (188.57 [142.52–244.91]). The detailed numbers are provided in Supplementary Table S2.

**Table 1.** Age standardized incidence, prevalence, mortality, DALYs rate and AAPC of ischemic heart disease vs ischemic stroke in youths and young adults (15–39 years) at global and regional level, 1990–2021, both sexes.

	Ischemic heart disease (Incidence rate, 95% UI)			Ischemic stroke (Incidence rate, 95% UI)		
	Age standardized rate in 1990 (per 100,000)	Age standardized rate in 2021 (per 100,000)	AAPC (95% CI)	Age standardized rate in 1990 (per 100,000)	Age standardized rate in 2021 (per 100,000)	AAPC (95% CI)
Global	35.03 (18.54 to 55.55)	36.86 (19.50 to 58.52)	0.25 (0.21 to 0.30)	13.15 (7.46 to 21.43)	12.84 (7.29 to 20.57)	−0.17 (−0.23 to −0.11)
Sex						
Male	42.97 (23.01 to 67.74)	44.33 (23.76 to 69.85)	0.23 (0.17 to 0.29)	11.91 (6.42 to 19.75)	12.23 (6.87 to 20.66)	0.08 (0.05 to 0.11)
Female	26.87 (13.94 to 43.26)	29.24 (15.22 to 46.84)	0.29 (0.25 to 0.34)	14.41 (7.98 to 23.55)	13.47 (7.52 to 21.89)	−0.39 (−0.48 to −0.30)
SDI						
High SDI	25.36 (13.11 to 40.68)	23.98 (12.34 to 38.38)	−0.28 (−0.35 to −0.21)	11.79 (6.19 to 19.65)	10.67 (5.79 to 17.49)	−0.31 (−0.37 to −0.25)
High-middle SDI	40.97 (22.47 to 64.02)	41.74 (22.75 to 65.54)	0.12 (0.07 to 0.17)	14.32 (8.19 to 23.01)	12.90 (7.30 to 20.73)	−0.35 (−0.37 to −0.32)
Middle SDI	34.70 (18.18 to 55.24)	37.30 (19.63 to 59.31)	0.33 (0.27 to 0.39)	12.59 (6.97 to 20.50)	12.56 (7.06 to 20.23)	−0.01 (−0.15 to 0.14)
Low-middle SDI	38.44 (20.16 to 61.51)	41.18 (21.77 to 64.80)	0.38 (0.30 to 0.46)	13.16 (7.23 to 21.43)	13.55 (7.81 to 21.55)	0.08 (0.02 to 0.15)
Low SDI	33.89 (17.50 to 54.67)	34.79 (17.98 to 56.03)	0.14 (0.08 to 0.20)	15.13 (8.33 to 24.66)	14.08 (8.10 to 22.47)	−0.23 (−0.25 to −0.21)
World Bank Region						
East Asia & Pacific	29.29 (15.38 to 46.67)	30.89 (16.27 to 49.13)	0.23 (0.10 to 0.36)	11.34 (6.12 to 18.70)	12.55 (6.98 to 20.25)	0.24 (0.07 to 0.42)
Europe & Central Asia	40.68 (22.90 to 62.59)	38.30 (21.20 to 59.54)	−0.17 (−0.21 to −0.14)	15.68 (9.12 to 24.81)	12.52 (7.23 to 19.83)	−0.68 (−0.82 to −0.54)
Latin America & Caribbean	32.39 (16.83 to 52.15)	30.95 (16.11 to 49.68)	−0.11 (−0.13 to −0.09)	13.03 (7.13 to 21.47)	9.22 (4.70 to 15.47)	−1.20 (−1.36 to −1.05)
Middle East & North Africa	55.68 (29.64 to 87.51)	60.13 (32.29 to 93.87)	0.29 (0.26 to 0.33)	20.06 (12.56 to 30.38)	18.26 (11.64 to 27.44)	−0.34 (−0.39 to −0.29)
North America	30.01 (13.81 to 51.25)	21.62 (10.87 to 34.88)	−1.58 (−1.78 to −1.37)	12.18 (5.93 to 21.02)	11.25 (5.92 to 18.79)	−0.24 (−0.29 to −0.18)
South Asia	41.46 (21.90 to 66.20)	44.69 (23.76 to 70.50)	0.41 (0.31 to 0.51)	11.37 (5.83 to 19.13)	11.59 (6.26 to 19.80)	0.08 (0.01 to 0.15)
Sub-Saharan Africa	30.69 (15.55 to 49.60)	31.47 (15.98 to 50.92)	0.07 (0.05 to 0.10)	17.84 (10.23 to 28.71)	16.13 (9.48 to 25.55)	−0.32 (−0.38 to −0.27)
World Bank Income						
World Bank High Income	24.69 (12.80 to 39.44)	22.62 (11.67 to 36.09)	−0.44 (−0.53 to −0.35)	11.95 (6.29 to 19.88)	10.21 (5.54 to 16.79)	−0.52 (−0.63 to −0.42)
World Bank Upper Middle Income	38.54 (20.77 to 60.60)	39.95 (21.41 to 63.01)	0.15 (0.08 to 0.23)	12.42 (6.91 to 20.17)	12.01 (6.66 to 19.47)	−0.10 (−0.15 to −0.05)
World Bank Lower Middle Income	37.40 (19.64 to 59.89)	39.81 (20.93 to 63.07)	0.35 (0.27 to 0.43)	14.06 (7.77 to 22.86)	13.71 (7.38 to 21.90)	−0.13 (−0.19 to −0.08)
World Bank Low Income	33.33 (17.24 to 53.25)	34.29 (17.81 to 54.85)	0.16 (0.09 to 0.23)	17.35 (10.13 to 27.46)	15.46 (9.23 to 24.16)	−0.38 (−0.43 to −0.34)
Health System						
Advanced Health System	33.77 (18.24 to 52.85)	31.83 (17.19 to 49.86)	−0.23 (−0.27 to −0.19)	13.81 (7.58 to 22.58)	11.63 (6.49 to 18.70)	−0.54 (−0.56 to −0.52)
Basic Health System	33.97 (17.86 to 53.86)	36.35 (19.16 to 57.66)	0.29 (0.21 to 0.36)	12.77 (7.20 to 20.61)	13.07 (7.49 to 20.82)	0.09 (0.04 to 0.14)
Limited Health System	38.59 (20.27 to 61.90)	40.74 (21.47 to 64.49)	0.31 (0.23 to 0.40)	12.78 (6.81 to 21.18)	12.85 (7.19 to 20.83)	0.02 (0.01 to 0.04)
Minimal Health System	29.54 (14.73 to 47.84)	31.40 (15.98 to 50.83)	0.21 (0.10 to 0.32)	17.20 (9.73 to 27.73)	16.08 (9.52 to 25.05)	−0.24 (−0.29 to −0.19)

	Ischemic heart disease (Prevalence rate, 95% UI)			Ischemic stroke (Prevalence rate, 95% UI)		
	Age standardized rate in 1990 (per 100,000)	Age standardized rate in 2021 (per 100,000)	AAPC (95% CI)	Age standardized rate in 1990 (per 100,000)	Age standardized rate in 2021 (per 100,000)	AAPC (95% CI)
Global	178.24 (139.25 to 222.88)	188.57 (142.52 to 244.91)	0.20 (0.18 to 0.22)	221.04 (187.79 to 257.30)	205.24 (178.00 to 233.75)	−0.28 (−0.31 to −0.26)
Sex						
Male	205.26 (161.10 to 256.07)	216.69 (164.53 to 280.25)	0.22 (0.20 to 0.24)	190.81 (162.00 to 221.83)	180.39 (156.54 to 206.06)	−0.20 (−0.22 to −0.18)
Female	150.45 (117.07 to 190.05)	159.89 (119.58 to 208.55)	0.17 (0.15 to 0.20)	251.98 (214.14 to 293.31)	230.80 (200.20 to 262.25)	−0.35 (−0.38 to −0.31)
SDI						
High SDI	136.12 (108.06 to 167.72)	141.72 (109.65 to 180.86)	0.10 (0.04 to 0.15)	225.85 (191.59 to 263.19)	212.93 (184.06 to 243.18)	−0.25 (−0.31 to −0.18)
High-middle SDI	221.80 (173.13 to 279.47)	224.11 (178.72 to 292.71)	0.03 (0.01 to 0.04)	239.96 (203.29 to 280.17)	206.94 (178.21 to 237.04)	−0.51 (−0.54 to −0.48)
Middle SDI	181.62 (139.58 to 230.37)	196.95 (146.82 to 259.28)	0.26 (0.22 to 0.30)	216.91 (182.14 to 254.86)	200.89 (171.21 to 231.87)	−0.29 (−0.32 to −0.26)
Low-middle SDI	175.14 (137.56 to 219.58)	194.43 (146.77 to 252.65)	0.41 (0.37 to 0.44)	198.93 (169.76 to 230.52)	198.38 (173.23 to 225.45)	−0.04 (−0.08 to 0.01)
Low SDI	147.47 (116.71 to 182.07)	158.00 (120.01 to 203.31)	0.24 (0.22 to 0.27)	235.01 (205.99 to 266.97)	220.17 (197.27 to 245.03)	−0.26 (−0.29 to −0.23)
World Bank Region						
East Asia & Pacific	166.16 (126.63 to 213.26)	174.93 (129.25 to 232.18)	0.16 (0.11 to 0.20)	215.87 (180.69 to 254.57)	202.29 (171.25 to 234.37)	−0.23 (−0.26 to −0.20)
Europe & Central Asia	217.18 (175.78 to 265.23)	209.67 (162.90 to 255.78)	−0.13 (−0.21 to −0.06)	225.49 (195.96 to 257.85)	192.41 (170.66 to 216.00)	−0.58 (−0.63 to −0.54)
Latin America & Caribbean	169.38 (134.75 to 206.79)	169.83 (130.29 to 215.15)	0.01 (−0.03 to 0.04)	188.50 (160.69 to 218.84)	152.99 (133.41 to 173.90)	−0.74 (−0.78 to −0.70)
Middle East & North Africa	262.69 (218.93 to 314.47)	296.94 (235.85 to 371.02)	0.43 (0.41 to 0.44)	335.88 (300.68 to 374.70)	301.25 (275.04 to 328.76)	−0.42 (−0.45 to −0.38)
North America	137.65 (103.03 to 181.29)	123.83 (90.10 to 165.16)	−0.44 (−0.56 to −0.32)	307.26 (252.48 to 367.39)	287.46 (241.00 to 336.22)	−0.32 (−0.53 to −0.10)
South Asia	184.12 (139.41 to 239.32)	206.49 (150.68 to 274.75)	0.44 (0.40 to 0.48)	162.83 (133.38 to 195.08)	160.91 (132.88 to 189.91)	−0.07 (−0.11 to −0.02)
Sub-Saharan Africa	134.35 (106.90 to 164.85)	145.25 (111.09 to 184.44)	0.25 (0.23 to 0.26)	288.49 (252.93 to 327.47)	264.39 (237.18 to 294.06)	−0.33 (−0.36 to −0.30)
World Bank Income						
World Bank High Income	136.00 (109.21 to 165.85)	135.17 (106.44 to 171.46)	−0.04 (−0.11 to 0.03)	217.92 (185.99 to 252.88)	203.38 (177.21 to 231.25)	−0.29 (−0.33 to −0.24)
World Bank Upper Middle Income	206.20 (158.74 to 262.43)	213.60 (159.65 to 280.00)	0.23 (0.18 to 0.28)	223.51 (188.04 to 262.51)	199.10 (169.81 to 228.90)	−0.44 (−0.47 to −0.41)
World Bank Lower Middle Income	175.42 (136.11 to 222.01)	192.96 (144.02 to 253.17)	0.37 (0.33 to 0.40)	213.24 (180.02 to 249.19)	203.91 (176.12 to 234.05)	−0.19 (−0.22 to −0.17)
World Bank Low Income	147.71 (121.96 to 176.18)	156.91 (122.88 to 196.33)	0.11 (0.10 to 0.12)	273.70 (246.94 to 303.55)	241.37 (220.97 to 263.89)	−0.41 (−0.43 to −0.38)
Health System						
Advanced Health System	178.39 (142.38 to 219.79)	177.81 (138.37 to 226.41)	−0.07 (−0.15 to 0.01)	232.76 (198.84 to 269.59)	213.16 (185.96 to 241.72)	−0.35 (−0.40 to −0.30)
Basic Health System	183.93 (142.47 to 232.24)	197.37 (149.12 to 255.66)	0.24 (0.22 to 0.27)	229.44 (193.61 to 268.63)	211.37 (181.71 to 241.67)	−0.30 (−0.32 to −0.27)
Limited Health System	172.48 (132.74 to 219.73)	189.82 (140.61 to 250.36)	0.37 (0.33 to 0.40)	192.01 (162.16 to 224.53)	191.25 (161.25 to 219.60)	−0.05 (−0.08 to −0.01)
Minimal Health System	125.80 (103.16 to 150.54)	138.54 (108.76 to 171.67)	0.30 (0.22 to 0.39)	273.35 (247.41 to 301.77)	250.47 (230.07 to 273.47)	−0.33 (−0.35 to −0.30)

	Ischemic heart disease (Mortality rate, 95% UI)			Ischemic stroke (Mortality rate, 95% UI)		
	Age standardized rate in 1990 (per 100,000)	Age standardized rate in 2021 (per 100,000)	AAPC (95% CI)	Age standardized rate in 1990 (per 100,000)	Age standardized rate in 2021 (per 100,000)	AAPC (95% CI)
Global	7.86 (7.40 to 8.35)	6.73 (6.28 to 7.16)	−0.56 (−0.64 to −0.48)	0.68 (0.62 to 0.76)	0.51 (0.45 to 0.58)	−1.07 (−1.16 to −0.99)
Sex						
Male	10.22 (9.49 to 10.97)	9.34 (8.66 to 10.05)	−0.34 (−0.44 to −0.25)	0.69 (0.60 to 0.81)	0.58 (0.49 to 0.67)	−0.63 (−0.73 to −0.54)
Female	5.44 (4.75 to 6.15)	4.06 (3.65 to 4.52)	−1.02 (−1.11 to −0.94)	0.68 (0.63 to 0.75)	0.43 (0.36 to 0.53)	−1.47 (−1.52 to −1.41)
SDI						
High SDI	4.08 (3.95 to 4.23)	2.43 (2.16 to 2.78)	−1.53 (−1.67 to −1.40)	0.40 (0.37 to 0.42)	0.24 (0.20 to 0.30)	−1.61 (−1.73 to −1.49)
High-middle SDI	7.53 (7.01 to 8.05)	4.87 (4.44 to 5.39)	−1.85 (−2.09 to −1.61)	0.85 (0.76 to 0.94)	0.52 (0.46 to 0.59)	−1.91 (−2.11 to −1.70)
Middle SDI	8.28 (7.78 to 8.78)	7.37 (6.82 to 7.96)	−0.32 (−0.38 to −0.25)	0.75 (0.67 to 0.84)	0.55 (0.49 to 0.62)	−1.01 (−1.12 to −0.90)
Low-middle SDI	11.45 (10.22 to 12.79)	9.77 (8.87 to 10.68)	−0.53 (−0.63 to −0.44)	0.72 (0.58 to 0.89)	0.58 (0.47 to 0.74)	−0.72 (−0.79 to −0.66)
Low SDI	6.31 (5.25 to 7.54)	5.78 (5.07 to 6.56)	−0.42 (−0.60 to −0.25)	0.52 (0.39 to 0.71)	0.49 (0.38 to 0.66)	−0.20 (−0.23 to −0.16)
World Bank Region						
East Asia & Pacific	6.33 (5.75 to 6.93)	6.06 (5.39 to 6.79)	−0.15 (−0.21 to −0.09)	0.70 (0.61 to 0.81)	0.60 (0.50 to 0.71)	−0.62 (−0.77 to −0.47)
Europe & Central Asia	7.66 (7.41 to 7.93)	3.84 (3.58 to 4.13)	−2.33 (−2.47 to −2.18)	0.77 (0.73 to 0.82)	0.39 (0.36 to 0.43)	−2.23 (−2.36 to −2.11)
Latin America & Caribbean	6.27 (6.04 to 6.49)	4.58 (4.28 to 4.91)	−0.84 (−1.19 to −0.48)	0.71 (0.68 to 0.75)	0.31 (0.28 to 0.34)	−2.55 (−2.67 to −2.42)
Middle East & North Africa	17.06 (15.30 to 19.06)	10.51 (8.91 to 12.32)	−1.48 (−1.56 to −1.41)	2.00 (1.60 to 2.56)	1.35 (1.09 to 1.65)	−1.25 (−1.31 to −1.19)
North America	3.68 (3.57 to 3.79)	2.48 (2.31 to 2.62)	−1.20 (−1.35 to −1.04)	0.21 (0.20 to 0.22)	0.15 (0.14 to 0.16)	−1.05 (−1.69 to −0.42)
South Asia	12.08 (10.58 to 13.69)	10.67 (9.60 to 11.71)	−0.38 (−0.62 to −0.14)	0.43 (0.30 to 0.60)	0.33 (0.24 to 0.51)	−0.78 (−0.92 to −0.63)
Sub-Saharan Africa	4.47 (3.89 to 5.13)	3.89 (3.30 to 4.50)	−0.48 (−0.52 to −0.43)	0.64 (0.52 to 0.85)	0.54 (0.42 to 0.69)	−0.58 (−0.77 to −0.40)
World Bank Income						
World Bank High Income	4.35 (4.24 to 4.48)	2.34 (2.10 to 2.66)	−1.95 (−2.08 to −1.82)	0.42 (0.40 to 0.45)	0.21 (0.18 to 0.26)	−2.08 (−2.17 to −1.98)
World Bank Upper Middle Income	6.42 (5.93 to 6.92)	4.78 (4.32 to 5.32)	−1.30 (−1.44 to −1.15)	0.75 (0.67 to 0.85)	0.51 (0.45 to 0.58)	−1.26 (−1.43 to −1.08)
World Bank Lower Middle Income	11.83 (10.80 to 12.92)	9.83 (9.05 to 10.59)	−0.52 (−0.63 to −0.41)	0.75 (0.62 to 0.89)	0.56 (0.47 to 0.68)	−0.97 (−1.07 to −0.88)
World Bank Low Income	6.82 (5.65 to 8.19)	5.40 (4.42 to 6.54)	−0.77 (−0.80 to −0.73)	0.78 (0.59 to 1.02)	0.67 (0.50 to 0.93)	−0.54 (−0.66 to −0.42)
Health System						
Advanced Health System	5.64 (5.47 to 5.82)	3.14 (2.90 to 3.43)	−1.91 (−2.01 to −1.81)	0.57 (0.53 to 0.60)	0.31 (0.28 to 0.36)	−2.19 (−2.38 to −2.00)
Basic Health System	7.58 (7.09 to 8.14)	6.58 (5.96 to 7.20)	−0.45 (−0.51 to −0.39)	0.85 (0.77 to 0.95)	0.67 (0.58 to 0.76)	−0.83 (−0.92 to −0.74)
Limited Health System	10.68 (9.53 to 11.97)	8.90 (8.10 to 9.68)	−0.59 (−0.80 to −0.39)	0.52 (0.40 to 0.69)	0.41 (0.32 to 0.56)	−0.75 (−0.82 to −0.68)
Minimal Health System	3.78 (3.01 to 4.86)	4.19 (3.27 to 5.27)	0.32 (0.10 to 0.55)	0.61 (0.43 to 0.85)	0.64 (0.46 to 0.92)	0.28 (0.23 to 0.34)

	Ischemic heart disease (Dalys rate, 95% UI)			Ischemic stroke (Dalys rate, 95% UI)		
	Age standardized rate in 1990 (per 100,000)	Age standardized rate in 2021 (per 100,000)	AAPC (95% CI)	Age standardized rate in 1990 (per 100,000)	Age standardized rate in 2021 (per 100,000)	AAPC (95% CI)
Global	458.81 (431.96 to 487.37)	394.56 (368.37 to 420.38)	−0.55 (−0.63 to −0.46)	73.83 (62.46 to 86.27)	59.76 (50.06 to 70.45)	−0.76 (−0.80 to −0.71)
Sex						
Male	590.75 (548.40 to 635.81)	543.54 (503.47 to 584.89)	−0.32 (−0.41 to −0.22)	66.35 (55.59 to 78.37)	57.90 (49.23 to 67.70)	−0.49 (−0.54 to −0.44)
Female	323.28 (282.09 to 365.90)	242.34 (218.21 to 269.44)	−0.93 (−0.99 to −0.86)	81.48 (67.80 to 96.74)	61.71 (50.32 to 74.78)	−0.99 (−1.04 to −0.94)
SDI						
High SDI	235.15 (227.16 to 243.62)	141.74 (126.31 to 162.51)	−1.48 (−1.60 to −1.35)	55.48 (45.50 to 66.69)	43.91 (34.86 to 54.85)	−0.72 (−0.81 to −0.63)
High-middle SDI	435.46 (404.83 to 465.85)	284.11 (259.30 to 314.28)	−1.80 (−2.03 to −1.56)	91.02 (76.82 to 106.96)	65.64 (54.07 to 78.43)	−1.07 (−1.18 to −0.97)
Middle SDI	484.20 (455.03 to 514.15)	433.07 (400.91 to 467.27)	−0.30 (−0.36 to −0.24)	78.08 (65.82 to 91.46)	63.11 (52.86 to 74.31)	−0.72 (−0.78 to −0.67)
Low-middle SDI	669.23 (596.79 to 748.48)	570.36 (517.46 to 623.25)	−0.46 (−0.58 to −0.34)	69.90 (57.16 to 83.62)	60.90 (49.87 to 73.94)	−0.46 (−0.48 to −0.43)
Low SDI	369.05 (306.80 to 441.60)	338.52 (297.13 to 384.49)	−0.21 (−0.37 to −0.06)	59.81 (48.47 to 74.01)	56.49 (45.75 to 69.35)	−0.27 (−0.33 to −0.21)
World Bank Region						
East Asia & Pacific	372.48 (338.32 to 407.44)	356.75 (318.38 to 399.73)	−0.14 (−0.20 to −0.08)	81.00 (66.96 to 97.24)	72.46 (59.22 to 87.22)	−0.41 (−0.48 to −0.35)
Europe & Central Asia	436.57 (421.55 to 451.91)	222.08 (207.10 to 238.73)	−2.69 (−3.07 to −2.31)	80.18 (68.86 to 92.04)	52.58 (43.34 to 62.27)	−1.39 (−1.48 to −1.31)
Latin America & Caribbean	365.20 (351.58 to 378.47)	271.79 (253.63 to 291.07)	−0.90 (−1.07 to −0.72)	58.43 (53.20 to 64.21)	31.76 (27.45 to 36.52)	−1.90 (−1.97 to −1.82)
Middle East & North Africa	1007.98 (904.37 to 1125.08)	626.95 (531.00 to 733.90)	−1.45 (−1.52 to −1.37)	163.46 (135.45 to 198.99)	120.71 (101.21 to 143.10)	−0.89 (−0.93 to −0.85)
North America	210.47 (203.90 to 217.50)	143.72 (133.88 to 152.13)	−1.14 (−1.30 to −0.98)	53.66 (40.79 to 68.44)	46.76 (35.35 to 59.54)	−0.52 (−0.61 to −0.43)
South Asia	703.82 (615.95 to 798.42)	620.38 (558.04 to 680.29)	−0.40 (−0.64 to −0.16)	47.39 (36.40 to 60.21)	41.55 (32.05 to 54.90)	−0.55 (−0.63 to −0.47)
Sub-Saharan Africa	261.73 (228.17 to 299.97)	229.75 (195.02 to 265.66)	−0.35 (−0.53 to −0.18)	73.04 (60.35 to 88.60)	64.31 (52.69 to 77.26)	−0.44 (−0.53 to −0.35)
World Bank Income						
World Bank High Income	250.46 (243.29 to 257.79)	136.92 (122.80 to 155.60)	−1.89 (−2.02 to −1.76)	55.21 (45.67 to 65.60)	40.27 (32.07 to 50.08)	−1.03 (−1.15 to −0.91)
World Bank Upper Middle Income	373.82 (344.91 to 402.89)	280.89 (254.22 to 312.34)	−1.24 (−1.38 to −1.10)	80.61 (67.43 to 95.34)	61.70 (50.78 to 73.39)	−1.00 (−1.09 to −0.92)
World Bank Lower Middle Income	690.68 (629.93 to 754.87)	573.48 (528.04 to 617.81)	−0.49 (−0.63 to −0.35)	75.36 (62.35 to 89.31)	62.09 (51.35 to 74.09)	−0.67 (−0.72 to −0.62)
World Bank Low Income	402.04 (333.38 to 482.54)	317.62 (260.03 to 384.03)	−0.77 (−0.81 to −0.74)	81.16 (66.46 to 99.54)	70.49 (56.60 to 87.90)	−0.53 (−0.60 to −0.46)
Health System						
Advanced Health System	322.53 (312.48 to 333.25)	181.98 (167.62 to 198.46)	−2.18 (−2.45 to −1.92)	67.35 (56.38 to 79.10)	48.68 (39.72 to 59.23)	−1.19 (−1.28 to −1.11)
Basic Health System	445.27 (416.59 to 477.61)	389.36 (353.04 to 425.85)	−0.42 (−0.48 to −0.36)	88.15 (74.49 to 102.73)	73.41 (61.36 to 86.49)	−0.62 (−0.67 to −0.57)
Limited Health System	622.52 (555.31 to 698.59)	517.82 (471.02 to 563.88)	−0.61 (−0.82 to −0.41)	56.07 (45.32 to 69.26)	49.50 (39.97 to 61.51)	−0.43 (−0.48 to −0.38)
Minimal Health System	221.84 (176.43 to 283.96)	246.98 (193.59 to 309.20)	0.44 (0.32 to 0.56)	69.08 (55.20 to 87.72)	68.77 (54.13 to 89.13)	−0.01 (−0.08 to 0.08)

After calculating the AAPC between 1990 and 2021, we observed a positive trend in age-standardized incidence (0.25% [95% CI, 0.21% to 0.30%], *p* < 0.001) and prevalence rates (0.20% [95% CI, 0.18% to 0.22%], *p* < 0.001) for IHD, while age-standardized mortality (−0.56% [−0.64% to −0.48%]) and DALY rates (−0.55% [−0.63% to −0.46%]) showed a decline. In IS, all age-standardized metrics exhibited a negative trend: incidence (−0.17% [−0.23% to −0.11%]), prevalence (−0.28% [−0.31% to −0.26%]), mortality (−1.07% [−1.16% to −0.99%]), and DALYs (−0.76% [−0.80% to −0.71%]), all with *p* < 0.001 (Table 1).

### Trends comparisons in incidence, prevalence, mortality, and DALYs by sex

In 2021, the age-standardized incidence rate for IHD among youth and young adults was higher in males than in females (44.33/100,000 [23.76–69.85] vs 29.24/100,000 [15.22–46.84]), whereas IS showed a higher rate in females than in males (13.47 vs 12.23 per 100,000) (Table 1). The AAPC in age-standardized IHD incidence for both sexes was positive from 1990 to 2021, with females experiencing a greater increase than males. Notably, while the AAPC of age-standardized incidence for IS in both sexes was negative overall from 1990 to 2021, males had a positive AAPC (0.08% [95% CI 0.05% to 0.11%], *p* < 0.001), whereas females had a negative AAPC (−0.39% [95% CI −0.48% to −0.30%], *p* < 0.001). For age-standardized mortality and DALYs rates from 1990 to 2021, both IHD and IS showed a decrease, with females showing a greater AAPC decline than males (IHD mortality: −1.02% vs. −0.34%, IHD DALYs: −0.93% vs. −0.32%; IS mortality: −1.47% vs. −0.63%, IS DALYs: −0.99% vs. −0.49%).

### Trends comparisons in incidence, prevalence, mortality, and DALYs by regions

In 2021, the highest youth and young adult burden of IHD (as measured by age-standardized incidence, prevalence, mortality, and DALYs rates) was observed in Europe and Central Asia, the Middle East and North Africa, and South Asia (Table 1). Conversely, the IS burden was highest in East Asia, the Pacific, the Middle East, North Africa, and sub-Saharan Africa. The IHD burden was predominant in the low-middle, middle, and high-middle SDI regions, whereas the IS burden was concentrated in the low- and low-middle SDI regions. The upper and lower incomes of the World Bank had a higher IHD burden, whereas low-income countries had a higher IS burden. The IHD burden was also concentrated in basic and limited health systems, whereas the IS burden was mostly in minimal health systems. High SDI, World Bank high-income, and advanced health systems consistently showed lower burdens for both IHD and IS.

**Figure 1.**
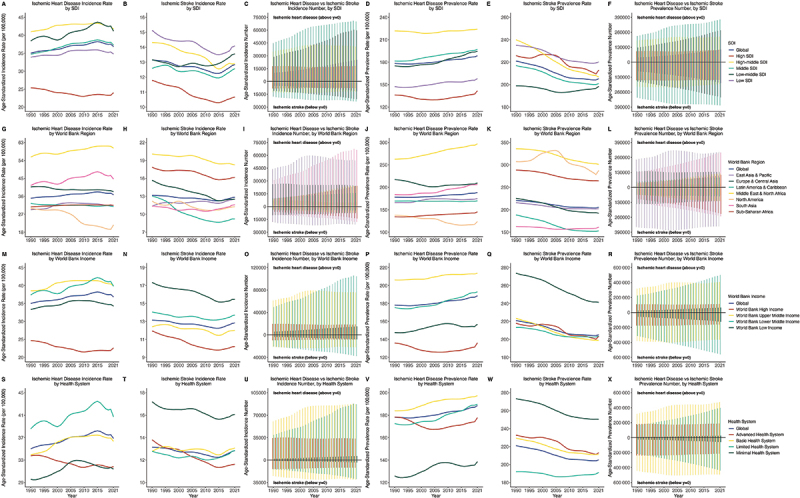
Trend of age standardized incidence, prevalence rate and number of ischemic heart disease vs ischemic stroke in youths and young adults (15–39years) at global and regional level, 1990–2021, both sexes.

From 1990 to 2021, age-standardized incidence rates of IHD and IS showed that high SDI regions consistently had the lowest rates, whereas low-middle SDI regions showed the largest increases (Figure 1). The most pronounced decline in IHD after 2000 was observed in North America, while the Middle East and North Africa consistently had the highest rates for both diseases, with increasing trends for IHD (AAPC, 0.29%) but declining trends for IS (−0.34%). The incidence of most SDI, World Bank income, and health system regions in IS showed some resurgence after 2015. All metrics showed higher age standardized numbers across lower SDI, South Asia, East Asia and Pacific, World Bank upper and lower middle income, and basic and limited health systems in both IHS and IS from 1990 to 2021.

**Figure 2.**
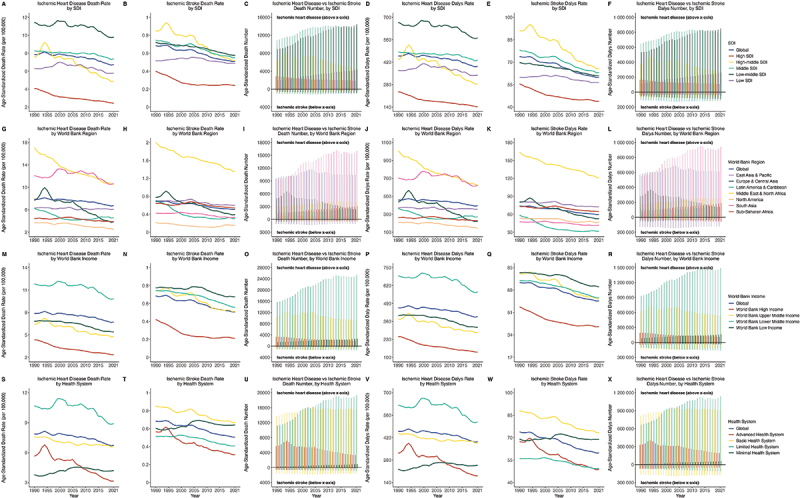
Trend of age standardized mortality, DALYs rate and number of ischemic heart disease vs ischemic stroke in youths and young adults (15–39years) at global and regional level, 1990–2021, both sexes.

For IHD, the AAPC of the age-standardized incidence rate was only negative in the high SDI region (−0.28%), while other SDI regions showed positive trends, with Low-middle SDI having the highest AAPC (0.38%) (Figure 1 and Table 1). In contrast, for IS, Low-middle SDI was the only region with a positive AAPC (0.08% [95%]), whereas other SDI regions showed negative trends. North America had the lowest AAPC for IHD (−1.58%), whereas South Asia had the highest (0.41%). For IS, Latin America and the Caribbean had the lowest AAPC (−1.20%) and East Asia and Pacific the highest (0.24%). The World Bank high income was the only income level with a negative AAPC for IHD (−0.44%), and all income levels had negative AAPC for IS. Advanced health system regions had notably lower AAPC for both IHD and IS than other health systems. Other metric results are shown in (Figure 2 and Supplementary Results).

### Trends comparisons in incidence, prevalence, mortality, and DALYs by country location

**Figure 3.**
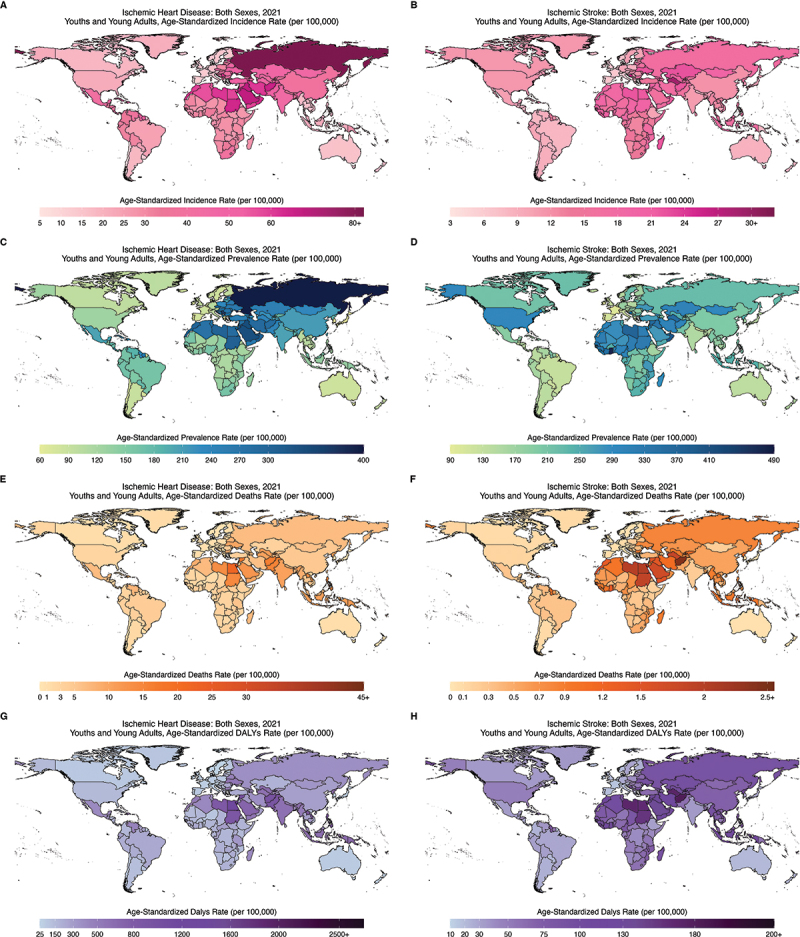
Age standardized incidence, prevalence, mortality, DALYs rate ischemic heart disease vs ischemic stroke in youths and young adults (15–39years) at country level, 2021, both sexes.

In 2021, the global age-standardized incidence, prevalence, mortality, and DALYs rates and numbers for youth and young adults showed notable geographic differences (Figure 3 and Supplementary Table S3-S10). For IHD incidence, the lowest rate was observed in Portugal (7.45 per 100,000 [2.77–13.68]) and the highest in Russia (81.90 per 100,000 [45.75–127.05]), whereas for IS, the lowest rate was in Cyprus (5.08 [2.02–9.63]) and the highest in Ghana (32.95 [22.31–47.80]). Regarding prevalence, IHD was lowest in South Korea (67.01 [51.28–85.32]) and highest in Russia (397.07 [286.03–536.07]), whereas IS was the lowest in France (97.90 [88.28–108.60]) and highest in Ghana (479.59 [444.62–515.70]). For mortality, IHD was lowest in Sweden (0.39 [0.33–0.47]) and highest in Nauru (46.49 [32.81–65.78]), while IS was lowest in Israel (0.02 [0.02–0.03]) and highest in Nauru (2.53 [1.44–4.21]). Lastly, DALY rates for IHD were lowest in Sweden (25.90 [21.58–30.96]) and highest in Nauru (2638.67 [1859.14–3745.41]), while for IS, lowest in Switzerland (14.81 [10.02–20.75]) and highest in Nauru (233.36 [164.08–336.93]).

### Risk factors to ischemic heart disease and ischemic stroke related DALYs

**Figure 4.**
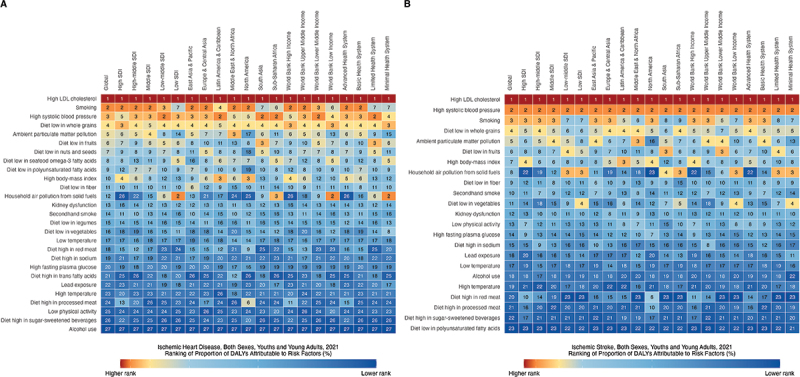
Age standardized proportion of DALYs attributable to five level 1 and 2 risk factors of ischemic heart disease vs ischemic stroke in youths and young adults (15–39 years) at global and regional level, 1990 vs 2021, both sexes.

Figure 4 shows the change in the age-standardized proportions of DALYs attributable to the five main risk factors for IHD and IS from 1990 to 2021. Globally, metabolic risks increased for both IHD and IS, whereas other risk factors decreased over time. In 2021, dietary risks contributed the most to IHD (66.2%), whereas metabolic risks were the largest contributors to IS (60.5%). Tobacco and dietary risks were more significant for IHD than for IS.

In 2021, regions with higher SDI had greater contributions of metabolic risks and tobacco to IHD-related DALYs, while environmental and occupational risks, dietary risks, and air pollution contributed more prominently to IHD-related DALYs in regions with lower SDI (Figure 4). Similar patterns were observed for the IS. For both IHD and IS, environmental and occupational risks and air pollution contributed more to lower income DALYs in the World Bank, whereas metabolic risks and tobacco had a larger impact on higher income in the World Bank. Dietary risks contributed more to the World Bank’s high- and low-income and limited and minimal health systems.

**Figure 5.**
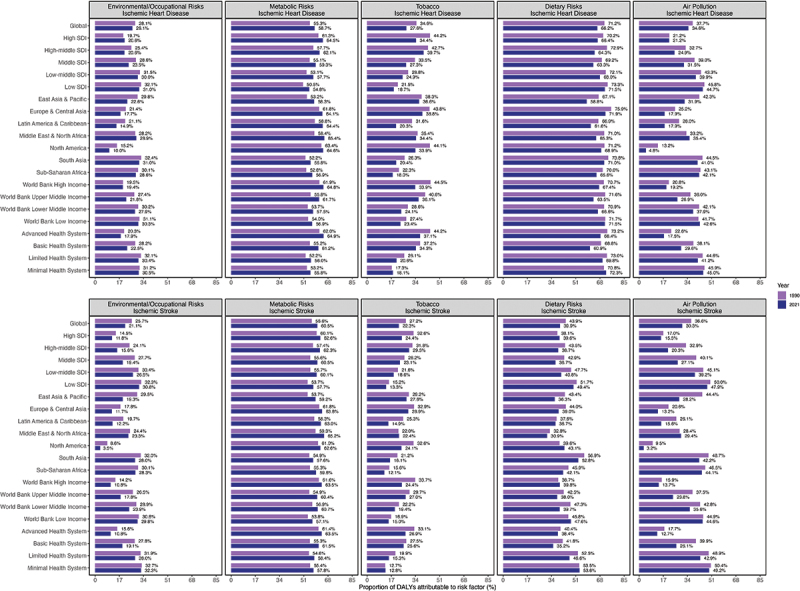
Age standardized proportion ranking of DALYs attributable specific risk factors of ischemic heart disease vs ischemic stroke in youths and young adults (15–39 years) at global and regional level, 2021, both sexes.

We ranked the age-standardized proportion of DALYs attributable to 27 risk factors for IHD and 23 risk factors for IS (Figure 5). High LDL cholesterol, smoking, high systolic blood pressure, diets low in whole grains, ambient particulate matter pollution, and diets low in fruits were the top risk factors for both IHD and IS across most regions. A diet high in processed meat in IHD and a diet high in red meat in IS were two high risks in North America. Household air pollution from solid fuels was a major risk factor in low- and low-middle SDI, sub-Saharan Africa, World Bank low-income, and limited and minimal health system regions for both IHD and IS. High BMI was a significant risk factor for both IHD and IS in countries with higher SDI, higher income, and advanced and basic health systems. For IS, a diet low in vegetables was at high risk for low SDI, sub-Saharan Africa, World Bank low income, and minimal health system.

## Discussion

This systematic analysis of GBD data from 1990 to 2021 highlights key trends and disparities in IHD and IS between youth and young adults aged 15–39 years. Significant differences were found in the regional distribution, temporal trends, sex differences, and associated risk factors between IHD and IS. These insights are critical for developing targeted public health interventions to mitigate the IHD and IS burdens in the young population.

Our findings are consistent with previous studies that used GBD data from 1990 to 2019, which also reported increasing incidence and decreasing mortality and DALYs of IHD and IS in young populations [4–6,15,27]. Previous studies have emphasized that risk factors vary considerably across regions according to income levels [28,29], with high systolic blood pressure, high BMI, and smoking identified as the main contributors to IHD and IS risk [9,11–13,21,30]. Regarding sex differences, several studies have reported a higher incidence of IHD among young males than among females, which aligns with our findings [4,10].

This study contributes to the existing literature by comparing the global burden and trends of IHD and IS among youth and young adults, thereby revealing key regional and socioeconomic disparities. By leveraging the GBD data and extending the analysis to 2021, we offer the most current evident on regional IHD and IS trends, addressing gaps in previous studies and informing timely public health strategies. We identified specific risk factors contributing to the disease burden, offering practical insights into proactive interventions and public health policies. Our findings highlight the importance of tailored strategies that address distinct risk profiles and challenges for young populations across different regions and socioeconomic contexts.

### Differences in disease burden and trends

In 2021, the global age-standardized incidence rate of IHD among youth and young adults was approximately three times higher than that of IS (36.86 vs 12.84). Age-standardized mortality and DALY rates were also significantly higher in the IHD group, indicating a greater burden of fatality and disability. Conversely, IS had a higher age-standardized prevalence rate than IHD (205.24 vs 188.57), suggesting a longer duration or lower case-fatality rate for IS.

Over the past three decades, the global age-standardized incidence and prevalence of IHD among youth and young adults have increased [4,9,15,30], while mortality and DALYs have declined, suggesting that advances in medical care have improved survival and reduced disease severity [10,30]. In contrast, all age-standardized metrics for IS have declined, indicating effective prevention strategies [5,15]. However, the resurgence in IS incidence rates post-2015 in several regions raises concerns about emerging risk factors or more health system disparities. Improvements in case detection and reporting systems, particularly in low- and middle-income countries, may have contributed to an increase in incidence by reducing underdiagnosis. Disparities in healthcare access and preventive services could also play a role, with regions facing resource constraints showing a slower decline or even reversal in IS incidence trends. This resurgence trend warrants closer monitoring. Further research should assess the statistical significance across regions and clarify whether these patterns reflect true epidemiological changes or improvements in surveillance and reporting.

### Regional disparities

Our analysis revealed significant regional disparities in IHD and IS burden among youth and young adults. For IHD, higher burdens were observed in low-middle to high-middle SDI regions, notably in Europe and Central Asia, the Middle East and North Africa, and South Asia. The persistent increase in IHD incidence in the Middle East and North Africa (AAPC of 0.29%) may be due to rapid urbanization, lifestyle changes, and inadequate risk factor management [31,32]. In particular, the interaction between uneven socioeconomic development, as captured by an unevenly distributed SDI, and region-specific cultural norms may exacerbate IHD risk. Specifically, rapid socioeconomic improvements have led to increased urbanization and associated lifestyle changes, such as reduced physical activity and dietary shifts. In the Middle East and North Africa, when these socioeconomic transitions interact with local cultural norms, such as the widespread use of tobacco products and dietary habits rich in saturated fats among younger populations, they may further intensify the IHD risk associated with rapid urbanization. Conversely, North America saw the most notable decline, likely due to effective public health initiatives and improved healthcare access [8,33,34]. For IS, higher burdens were observed in low- and low-middle SDI regions, such as sub-Saharan Africa, which may be influenced by limited healthcare infrastructure, lack of preventive services, and higher exposure to some risks [30,35].

In low-income regions, limited healthcare resources may hinder effective screening and management of risk factors, leading to higher mortality and DALYs rates for IS [14,35,36]. Higher exposure to environmental hazards, including air pollution and unsafe working conditions, may exacerbate the risk of IS-related diseases [11,21,35]. The higher IHD burden in the upper and lower-middle income and middle- and high-middle SDI regions could reflect a transition phase where rapid economic development leads to lifestyle changes that increase IHD-related risk, but healthcare systems have not yet fully adapted to manage the resulting disease burden [32].

### Sex differences in disease burden and trends

Sex-specific analyses in youths and young adults revealed that males had higher age-standardized incidence rates of IHD than females (44.33 vs 29.24 per 100,000), which may be associated with higher cardiovascular-related risk behaviors such as smoking, alcohol use, and occupational exposure among males. Hormonal differences may also play a role, as estrogen is thought to provide cardiovascular-related protection for premenopausal females [36–38].

Conversely, despite the overall decline, females had higher age-standardized incidence rates of IS than males (13.47 vs 12.23 per 100,000), warranting further investigation [39,40]. Possible explanations may include pregnancy-related complications (e.g. preeclampsia and gestational hypertension) and autoimmune disorders, although these remain hypotheses and require further empirical validation in young populations. Positive AAPC in IS incidence among males suggests a need for intensified management, while negative AAPC in females may indicate better effectiveness of current prevention strategies in the young female population [27,30].

Age-standardized mortality and DALYs rates decreased in both diseases, with females showing a much lower AAPC. However, the higher incidence in males for IHD and in females for IS shows the importance of sex-specific strategies in prevention and management [41–43]. Although our study collected data by 5-year age groups, we primarily reported aggregated findings for the 15–39 year population. It is important to note that risk profiles may differ substantially between adolescents (15–19 years), who may be more affected by early-life exposures, and young adults approaching middle age (35–39 years), where cumulative effects of diet, physical inactivity, and metabolic risks are more pronounced. Future studies should further examine these narrower age bands to improve the translational value of findings.

### Risk factor contributions

Risk factor analysis highlights the substantial impact of modifiable risks on IHD and IS burden among youth and young adults [5,9,11,30]. Globally, metabolic risks were the leading contributors to IS-related DALYs and the second-largest for IHD, requiring more concentration [11,21,44]. We found that metabolic risks increased for both IHD and IS, while environmental, air pollution, tobacco, and dietary risks decreased globally over time. These downward trends are consistent with the impact of targeted interventions. At the policy level, smoking prevalence has partly declined due to comprehensive tobacco control policies, including taxation, while reductions in household air pollution in several low- and middle-income countries were associated with increased adoption of cleaner fuels [22,23]. These examples showed that targeted interventions on major modifiable risks can contribute to reductions in cardiovascular disease burden. For IHD, dietary risks contributed the most to DALYs, accounting for 66.2% in 2021 and decreasing from 71.2% in 1990. In regions with high SDI, tobacco use, dietary risks, and metabolic risks were major contributors to IHD-related DALYs, highlighting the influence of lifestyle factors in developed regions.

High LDL cholesterol, smoking, high systolic blood pressure, ambient particulate matter pollution, and a diet low in whole grains and fruits were the top risk factors for both IHD and IS across most regions [11,13,21]. High BMI also ranked among the top risk factors for both diseases, particularly in World Bank high-income countries, advanced health systems, and high- and high-middle SDI regions such as North America. The increasing prevalence of obesity in these regions is a critical concern, contributing to early metabolic syndrome and cardiovascular-related diseases in youth and young adults [5,9,14,30]. These findings align with those of previous studies conducted in both the young adult population and broader age ranges, emphasizing that obesity, hypertension, and poor dietary habits consistently emerge as significant contributors to cardiovascular disease burdens [4–7,33]. Previous research in all age populations similarly underscored metabolic and lifestyle factors, including smoking and dietary risks, as key determinants of IHD and IS burdens [7,9,11,33], highlighting their universal relevance across age groups.

In North America, a diet high in processed meat was the sixth risk factor for IHD, and a diet high in red meat ranked similarly for IS, emphasizing the impact of Western dietary patterns rich in red and processed meat [45]. This raises the need for dietary interventions promoting healthier eating habits among youth and young adults. In contrast, a diet low in vegetables was a significant risk factor for IS in low SDI, sub-Saharan Africa, World Bank low-income, and minimal health system areas, reflecting issues with access to nutritious foods [46,47]. Addressing food security and promoting vegetable consumption could be components of IS prevention in these regions.

In regions with lower SDI, household air pollution from solid fuels significantly contributed to both IHD and IS-related DALYs. Household air pollution is ranked as the sixth risk factor for IHD in low-middle SDI and limited health systems and was higher in low SDI, sub-Saharan Africa, and World Bank low-income countries [4]. For IS was the third or fourth leading risk factor in low- and low-middle SDI, World Bank low-income, South Asia, and Sub-Saharan Africa, reflecting the reliance on solid fuels for cooking and heating, which increases IS-related risks through prolonged exposure to particulate matter and other pollutants [11,35]. While our analysis quantified the attributable burden of individual risk factors, it is important to acknowledge that these risks often act through overlapping and mediated pathways. A formal causal framework was beyond the scope of this GBD-based study, but future research integrating pathway models or directed acyclic graphs could help clarify these multistep relationships.

### Differences and similarities in suggestion for IHD and IS interventions

The differences in burden, trends, and risk factors between IHD and IS call for shared and distinct intervention strategies for youth and young adults. Addressing metabolic risks through lifestyle modifications, such as promoting healthy diets, reducing processed and red meat intake [45], and encouraging physical activity to control BMI, is crucial for both diseases. In high SDI regions and high-income countries, interventions should focus on reducing smoking, managing high LDL cholesterol, and controlling hypertension [11,28,29,44], while in low-income and lower SDI regions, strategies should also address environmental/occupational risks such as household air pollution by encouraging clean fuels and cooking technologies [14,35]. In addition, apart from the similar dietary risk factors in both IHD and IS, different from the risk factors in IHD, the impact of low vegetable intake contributes more to IS-related DALYs, suggesting that healthy dietary interventions may contribute more to IS prevention in some direction [46,47].

Our findings highlight the importance of tailoring intervention strategies according to the specific needs of different populations. For IHD, youth and young adults in middle-income and transitioning economies may benefit from interventions that address the rapid lifestyle changes associated with urbanization and economic development [4,9,32]. For IS, improving access to healthcare and preventive services in low-income regions is essential to reducing the burden [36,48].

Given the higher IS burden among females, which may be related to pregnancy complications and contraceptive use, healthcare providers may monitor and manage more IS risk factors in females of reproductive age [39,42,49]. For males, given the increasing incidence of IS despite overall decrease, and the higher burden of males in IHD, more targeted interventions addressing modifiable factors such as smoking and unhealthy lifestyles are urgently needed [41].

### Potential influence of the COVID-19 pandemic on IHD and IS trends

The COVID-19 pandemic may have notably influenced recent trends in IHD and IS among youth and young adults. The direct cardiovascular impacts of SARS-CoV-2 infection, including heightened inflammation and endothelial dysfunction, might have contributed to the increased risk of acute cardiovascular events, including IHD and IS, even among younger populations [50,51]. Furthermore, pandemic-related disruptions to healthcare systems could have delayed the diagnosis and management of cardiovascular risk factors, exacerbating the disease burden [52]. Additionally, changes in lifestyle behaviors, such as increased sedentary behavior, unhealthy dietary habits, and heightened mental health stressors during lockdowns, might have also amplified existing cardiovascular risks. Future studies should aim to quantify these pandemic-related impacts and incorporate these findings into public health strategies that target youth and young adults.

### Future projections and trend of IHD and IS in youths and young adults

Given that the GBD 2021 study has provided projections extending to future years, it is also critical to consider future trends in IHD and IS among youth and young adults. Recent projections suggest a continued increase in the global burden of IHD among youth and young adults, indicating rising incidence, prevalence, mortality, and DALYs rates through 2050 [10]. Similarly, stroke burden projections suggest an increase in incidence, while predicting a decrease in mortality and DALYs rates, but still underscoring a continuing public health challenge that requires sustained attention and interventions [53]. These projections emphasize the importance of proactively addressing modifiable risk factors and strengthening healthcare systems to mitigate anticipated increases in cardiovascular disease burdens among youth and young adults.

### Strengths and limitations

The primary strength of this study lies in its comprehensive analysis and comparison of global trends in IHD and IS among youth and young adults from 1990 to 2021 using GBD data, which provides accurate estimates of disease burden and risk factors across diverse regions and countries. By focusing on the 15–39 years age group, this study fills a critical gap in cardiovascular-related research, which has often been underrepresented in previous studies. Identifying regional and sex disparities and key risk factors offers practical insights into targeted interventions to address the unique needs of the young population.

2This study has several limitations. The reliance on GBD data implies that our findings are contingent on the quality and availability of data across different countries and regions. In low-income regions, where healthcare infrastructure and reporting systems may be less developed, under-reporting or misclassification of cases is possible, potentially affecting the accuracy of our estimates. Second, the GBD methodology, although comprehensive, may not fully account for all potential confounding factors or emerging risk factors in youth and young adults, such as substance abuse or mental health disorders. Additionally, the use of DisMod-MR 2.1 includes inherent limitations, particularly its underlying smoothness assumption, might not accurately capture abrupt changes or localized variations in disease trends. The inability to analyze variations in disease burden by race or ethnicity within countries is another limitation, as it may mask important disparities in incidence, risk factors, and health outcomes among different population groups. The calculation of AAPC assumes a consistent trend in age-standardized rates over time. However, AAPC estimates may be affected by sudden changes in factors, such as national health policies, access to preventive healthcare services, advancements in medical technology, and the introduction of new medications. Future research with more granular data and longitudinal designs is needed. Finally, although we discussed the potential impact of the COVID-19 pandemic on IHD and IS, we could not accurately assess this issue as we lack direct evidence in this study. Future updates will be needed to clarify these associations.

## Conclusion

In conclusion, this study highlights the rising incidence and prevalence of IHD among youth and young adults, as well as the resurgence of IS in regions with lower SDI and limited health systems. These trends indicate a persistent burden that remains inadequately addressed by current prevention circumstances, such as the limited access to preventive screening and affordable healthcare services in low-income settings. Our findings underscore the urgent need for region-specific, proactive interventions targeting modifiable risk factors and expanding access to preventive healthcare. Future research should further investigate the drivers of these trends, including sex-specific and socioeconomic determinants, to inform policies that can reduce the long-term burden of IHD and IS on younger populations.

## Supplementary Material

Supplementary file.docx

GATHER checklist.docx

## Data Availability

Data will be made available on reasonable request. The datasets used in this study can also be found in an online database. The names of the databases are as follows: https://vizhub.healthdata.org/gbd-results/.
