# Supplementary material for: Global, regional, and national burden of ischemic heart disease and ischemic stroke and their risk factors in youths and young adults aged 15–39 years (1990–2021): a comparative analysis of risk factors from global burden of disease study 2021
Source: Glob Health Action. 2025 Oct 16;18(1):2560711. doi: 10.1080/16549716.2025.2560711 (PMC12724116; doi:10.1080/16549716.2025.2560711)
Supplement: Supplementary file.docx [file ZGHA_A_2560711_SM4922.docx]

**Supplementary Materials**

**Global, regional, and national burden of ischemic heart disease and ischemic stroke and their risk factors in youths and young adults aged 15–39 years (1990–2021): a comparative analysis of risks factors from Global Burden of Disease Study 2021**

Siwei Xie, Long Zhang, Yan Zhang, Zhi-Jie Zheng, Jianping Li, Shuduo Zhou

**Supplementary Results**

**Supplementary Table 1.** Definitions of the included risk factors

**Supplementary Table 2.** Age standardized incidence, prevalence, mortality, DALYs number and AAPC of ischemic heart disease vs ischemic stroke in youths and young Adults (15-39 years) at global and regional level, 1990-2021, both sexes

**Supplementary Table 3.** Age standardized incidence rate of ischemic heart disease vs ischemic stroke in youths and young Adults (15-39 years) at country level, 1990-2021, both sexes

**Supplementary Table 4.** Age standardized prevalence rate of ischemic heart disease vs ischemic stroke in youths and young Adults (15-39 years) at country level, 1990-2021, both sexes

**Supplementary Table 5.** Age standardized mortality rate of ischemic heart disease vs ischemic stroke in youths and young Adults (15-39 years) at country level, 1990-2021, both sexes

**Supplementary Table 6.** Age standardized DALYs rate of ischemic heart disease vs ischemic stroke in youths and young Adults (15-39 years) at country level, 1990-2021, both sexes

**Supplementary Table 7.** Age standardized incidence number of ischemic heart disease vs ischemic stroke in youths and young Adults (15-39 years) at country level, 1990-2021, both sexes

**Supplementary Table 8.** Age standardized prevalence number of ischemic heart disease vs ischemic stroke in youths and young Adults (15-39 years) at country level, 1990-2021, both sexes

**Supplementary Table 9.** Age standardized mortality number of ischemic heart disease vs ischemic stroke in youths and young Adults (15-39 years) at country level, 1990-2021, both sexes

**Supplementary Table 10.** Age standardized DALYs number of ischemic heart disease vs ischemic stroke in youths and young Adults (15-39 years) at country level, 1990-2021, both sexes

**Supplementary Results**

For each region types (SDI, World Bank region, World Bank income, health system), the AAPC of youths and young adults age standardized prevalence rates for ischemic heart disease were lowest in the following regions: High-middle SDI (0.03% [95% CI 0.01% to 0.04%]), North America (-0.44% [95% CI -0.56% to -0.32%]), World Bank high income (-0.04% [95% CI -0.11% to 0.03%]), advanced health system (-0.07% [95% CI -0.15% to 0.01%]); highest in the following regions: Low-middle SDI (0.41% [95% CI 0.37% to 0.44%]), South Asia (0.44% [95% CI 0.40% to 0.48%]), World Bank lower middle income (0.37% [95% CI 0.33% to 0.40%]), limited health system (0.37% [95% CI 0.33% to 0.40%]) (**Table 1**). As comparisons, the AAPC of youths and young adults age standardized prevalence rates for ischemic stroke were lowest in the following regions: High-middle SDI (-0.51% [95% CI -0.54% to -0.48%]), Latin America & Caribbean (-0.74% [95% CI -0.78% to -0.70%]), World Bank upper middle income (-0.44% [95% CI -0.47% to -0.41%]), advanced health system (-0.35% [95% CI -0.40% to 0.30%]); highest in the following regions: Low-middle SDI (-0.04% [95% CI -0.08% to 0.01%]), South Asia (-0.07% [95% CI -0.11% to -0.02%]), World Bank lower middle income (-0.19% [95% CI -0.22% to -0.17%]), limited health system (-0.05% [95% CI -0.08% to -0.01%]).

The AAPC of youths and young adults age standardized mortality rates for ischemic heart disease were lowest in the following regions: High-middle SDI (-1.85% [95% CI -2.09% to -1.61%]), Europe & Central Asia (-2.33% [95% CI -2.47% to -2.18%]), World Bank high income (-1.95% [95% CI -2.08% to -1.82%]), advanced health system (-1.91% [95% CI -2.01% to -1.81%]); highest in the following regions: Middle SDI (-0.32% [95% CI -0.38% to -0.25%]), East Asia & Pacific (-0.15% [95% CI -0.21% to -0.09%]), World Bank lower middle income (-0.52% [95% CI -0.63% to -0.41%]), minimal health system (0.32% [95% CI 0.10% to 0.55%]). As comparisons, the AAPC of youths and young adults age standardized mortality rates for ischemic stroke were lowest in the following regions: High-middle SDI (-1.91% [95% CI -2.11% to -1.70%]), Latin America & Caribbean (-2.55% [95% CI -2.67% to -2.42%]), World Bank high income (-2.08% [95% CI -2.17% to -1.98%]), advanced health system (-2.19% [95% CI -2.38% to -2.00%]); highest in the following regions: Low SDI (-0.20% [95% CI -0.23% to -0.16%]), Sub-Saharan Africa (-0.58% [95% CI -0.77% to -0.40%]), World Bank low income (-0.54% [95% CI -0.66% to -0.42%]), minimal health system (0.28% [95% CI 0.23% to 0.34%]).

The AAPC of youths and young adults age standardized DALYs rates for ischemic heart disease were lowest in the following regions: High-middle SDI (-1.80% [95% CI -2.03% to -1.56%]), Europe & Central Asia (-2.69% [95% CI -3.07% to -2.31%]), World Bank high income (-1.89% [95% CI -2.02% to -1.76%]), advanced health system (-2.18% [95% CI -2.45% to -1.92%]); highest in the following regions: Low SDI (-0.21% [95% CI -0.37% to -0.06%]), East Asia & Pacific (-0.14% [95% CI -0.20% to -0.08%]), World Bank lower middle income (-0.49% [95% CI -0.63% to -0.35%]), minimal health system (0.44% [95% CI 0.32% to 0.56%]). As comparisons, the AAPC of youths and young adults age standardized DALYs rates for ischemic stroke were lowest in the following regions: High-middle SDI (-1.07% [95% CI -1.18% to -0.97%]), Latin America & Caribbean (-1.90% [95% CI -1.97% to -1.82%]), World Bank high income (-1.03% [95% CI -1.15% to -0.91%]), advanced health system (-1.19% [95% CI -1.28% to -1.11%]); highest in the following regions: Low SDI (-0.27% [95% CI -0.33% to -0.21%]), East Asia & Pacific (-0.41% [95% CI -0.48% to -0.35%]), World Bank low income (-0.53% [95% CI -0.60% to -0.46%]), minimal health system (-0.01% [95% CI -0.08% to 0.08%]).

| Supplementary Table 1. Definitions of the included risk factors | | |
| --- | --- | --- |
| Risk Factor Category | Specific Risk Factor | Definition / Description |
| Environmental/Occupational Risks and Air Pollution | Ambient particulate matter pollution | Population-weighted annual average concentration of particulate matter smaller than 2.5 microns (PM2.5) in ambient air |
| Environmental/Occupational Risks and Air Pollution | Household air pollution from solid fuels | Exposure to indoor smoke from cooking or heating using solid fuels such as coal, wood, charcoal, and agricultural residues |
| Environmental/Occupational Risks and Air Pollution | Lead exposure | Population blood lead levels exceeding safe thresholds, causing cardiovascular harm |
| Environmental/Occupational Risks and Air Pollution | Low temperature | Daily mean temperature colder than the theoretical minimum risk exposure level (TMREL), defined as the temperature with the minimum mortality for all included causes. The population-weighted mean TMREL is 25.6°C |
| Environmental/Occupational Risks and Air Pollution | High temperature | Daily mean temperature warmer than the TMREL, the temperature with the minimum mortality  for all included causes. The population-weighted mean TMREL is 25.6°C, with a range of 21.3-26.6°C |
| Dietary Risks | Diet low in whole grains | Average daily consumption below recommended levels of whole grains (140-160g/day) |
| Dietary Risks | Diet low in fruits | Average daily consumption below recommended levels of fruits (340-350g/day) |
| Dietary Risks | Diet low in fiber | Average daily dietary fiber intake below recommended level (22-25g/day) |
| Dietary Risks | Diet low in vegetables | Average daily consumption below recommended levels of vegetables (280-320g/day) |
| Dietary Risks | Diet low in legumes (only for IHD) | Average daily consumption below recommended levels of legumes (90-100g/day) |
| Dietary Risks | Diet high in sodium | Average daily sodium intake above recommended levels (3g/day) |
| Dietary Risks | Diet high in red meat | Average daily consumption above recommended levels (23g/day) |
| Dietary Risks | Diet high in processed meat | Any intake (g/day) of meat preserved by smoking, curing, salting, or addition of chemical preservatives |
| Dietary Risks | Diet low in nuts and seeds (only for IHD) | Average daily consumption below recommended levels of nuts and seeds (19-24g/day) |
| Dietary Risks | Diet high in trans fatty acids (only for IHD) | Any intake (g/day) of trans fat from all sources, mainly partially hydrogenated vegetable oils and ruminant products |
| Dietary Risks | Diet high in sugar-sweetened beverages | Any intake (g/day) of beverages with at least 50 kcal per 226·8-g serving, including carbonated beverages, sodas, energy drinks, and fruit drinks, but excluding 100% fruit and vegetable juices |
| Dietary Risks | Diet low in seafood omega-3 fatty acids (only for IHD) | Average daily intake below recommended levels (430-470mg/day) |
| Dietary Risks | Diet low in omega-6 polyunsaturated fatty acids | Average daily intake below optimal levels (9-10% of total energy intake) |
| Other Behavioral Risks | Low physical activity | Objectively measured, total physical activity less than 3600 to 4400 MET-minutes per week. We use frequency, duration, and intensity of activity to calculate total metabolic equivalent (MET)-minutes per week. MET is the ratio of the working metabolic rate to the resting metabolic rate |
| Other Behavioral Risks | Alcohol use | Alcohol consumption in excess of the region-, age-, sex-, and year-specific TMREL |
| Tobacco | Smoking | Individuals who currently use any smoked tobacco product on a daily or occasional basis |
| Tobacco | \| Secondhand smoking \| \| --- \|  \|  \| \| --- \| | Current exposure to secondhand tobacco smoke at home, at work, or in other public places |
| Metabolic Risks | High fasting plasma glucose | Any level above the TMREL, which is 4.9-5.3 mmol/L |
| Metabolic Risks | High LDL cholesterol | LDL cholesterol concentration that exceeds the TMREL, which is 1.3 mmol/L (50 mg/dL) |
| Metabolic Risks | High systolic blood pressure | Systolic blood pressure levels above TMREL (≥110-115 mmHg) |
| Metabolic Risks | High body-mass index (BMI) | High body-mass index (BMI) for adults (ages 20+) is defined as BMI greater than 20-23 kg/m² |
| Metabolic Risks | Kidney dysfunction | A state of decreased kidney function |

| **Supplementary Table 2. Age standardized incidence, prevalence, mortality, DALYs number and AAPC of ischemic heart disease vs ischemic stroke in youths and young Adults (15-39 years) at global and regional level, 1990-2021, both sexes** | | | | | |
| --- | --- | --- | --- | --- | --- |
|  | **Ischemic heart disease (Incidence number, 95% UI)** | |  | **Ischemic stroke (Incidence number, 95% UI)** | |
|  | **Age standardized number in 1990** | **Age standardized number in 2021** |  | **Age standardized number in 1990** | **Age standardized number in 2021** |
| Global | 136338.9 (71476.3 to 217601.5) | 214130.0 (113226.4 to 339946.3) |  | 54840.9 (30025.2 to 89806.8) | 75625.7 (42885.8 to 121188.5) |
| Sex |  |  |  |  |  |
| Male | 84099.2 (44638.6 to 133321.0) | 130005.4 (69633.6 to 204858.9) |  | 24979.1 (13344.2 to 41547.9) | 39175.6 (21874.4 to 63699.5) |
| Female | 52239.7 (26794.2 to 84734.6) | 84124.6 (43789.9 to 134783.3) |  | 29861.9 (16392.2 to 49026.0) | 36450.1 (20482.1 to 58591.2) |
| SDI |  |  |  |  |  |
| High SDI | 17638.5 (9113.6 to 28260.8) | 18193.7 (9429.9 to 28996.8) |  | 8195.5 (4310.5 to 13651.1) | 7809.7 (4270.2 to 12737.2) |
| High-middle SDI | 35049.7 (19139.3 to 54922.4) | 40800.5 (22452.2 to 63618.0) |  | 12633.0 (7197.2 to 20333.4) | 11901.7 (6816.02 to 19022.5) |
| Middle SDI | 44644.1 (23036.2 to 71787.8) | 70153.7 (37031.2 to 111302.7) |  | 17843.1 (9751.8 to 29237.1) | 23442.4 (13205.2 to 37694.8) |
| Low-middle SDI | 28771.3 (14874.8 to 46468.0) | 59610.7 (31302.3 to 94213.8) |  | 11017.1 (5961.9 to 18068.5) | 20812.5 (11929.5 to 33197.8) |
| Low SDI | 10091.2 (5110.2 to 16476.1) | 25204.5 (12788.2 to 41079.4) |  | 5099.0 (2773.7 to 8363.0) | 11603.3 (6595.3 to 18624.5) |
| World Bank Region |  |  |  |  |  |
| East Asia & Pacific | 43728.3 (22670.5 to 70329.3) | 53924.0 (28654.2 to 85166.9) |  | 18129.6 (9667.4 to 30035.0) | 21072.4 (11823.9 to 33858.2) |
| Europe & Central Asia | 26057.1 (14650.9 to 40073.6) | 24297.3 (13517.1 to 37635.3) |  | 10108.6 (5877.7 to 15990.4) | 7654.0 (4457.6 to 12081.1) |
| Latin America & Caribbean | 9990.0 (5112.0 to 16230.3) | 15719.1 (8165.9 to 25258.2) |  | 4336.3 (2338.8 to 7183.7) | 4737.4 (2412.2 to 7957.1) |
| Middle East & North Africa | 9046.5 (4755.0 to 14323.1) | 24145.4 (12992.2 to 37657.6) |  | 3814.7 (2378.9 to 5807.5) | 7194.9 (4588.4 to 10809.4) |
| North America | 6986.7 (3223.2 to 11895.2) | 5374.4 (2708.6 to 8657.5) |  | 2792.4 (1366.0 to 4809.1) | 2785.5 (1468.1 to 4646.7) |
| South Asia | 30883.2 (16101.6 to 49681.6) | 66197.4 (34991.2 to 104766.7) |  | 9358.2 (4738.9 to 15876.5) | 18042.8 (9677.1 to 29789.2) |
| Sub-Saharan Africa | 9435.5 (4675.3 to 15461.0) | 24235.4 (12070.0 to 39688.6) |  | 6219.8 (3524.5 to 10072.0) | 14059.1 (8179.2 to 22365.6( |
| World Bank Income |  |  |  |  |  |
| World Bank High Income | 19545.1 (10116.1 to 31212.3) | 18776.1 (7744.9 to 24619.7) |  | 9519.6 (5014.4 to 15844.8) | 8222.9 (4484.8 to 13469.8) |
| World Bank Upper Middle Income | 61077.5 (32547.5 to 96753.0) | 75809.1 (40969.4 to 118845.1) |  | 21006.1 (11551.6 to 34282.2) | 21911.4 (12268.5 to 35370.6) |
| World Bank Lower Middle Income | 49550.7 (25668.7 to 79977.1) | 104188.1 (54505.9 to 165603.5) |  | 20680.2 (11318.5 to 33835.7) | 37622.0 (21511.3 to 60214.5) |
| World Bank Low Income | 6021.0 (3050.7 to 9755.1) | 15189.3 (7744.9 to 24619.7) |  | 3581.7 (2065.8 to 5700.8) | 7813.1 (4610.9 to 12287.5) |
| Health System |  |  |  |  |  |
| Advanced Health System | 34356.3 (18562.5 to 53730.4) | 33495.3 (18177.9 to 52290.5) |  | 14096.7 (7749.3 to 23047.2) | 11795.5 (6628.5 to 18897.0) |
| Basic Health System | 60386.1 (31303.4 to 96664.4) | 87030.8 (46141.5 to 137434.3) |  | 24698.4 (13751.1 to 40048.8) | 30474.1 (17555.3 to 48416.9) |
| Limited Health System | 39163.0 (20283.6 to 63376.4) | 87026.1 (45490.8 to 138489.9) |  | 14480.9 (7632.3 to 24208.6) | 29521.0 (16386.9 to 48006.9) |
| Minimal Health System | 2289.3 (1114.5 to 3765.5) | 6410.9 (3192.4 to 10535.6) |  | 1511.7 (843.8 to 2453.8) | 3779.1 (2215.6 to 5922.3) |
|  | **Ischemic heart disease (Prevalence number, 95% UI)** | |  | **Ischemic stroke (Prevalence number, 95% UI)** | |
|  | **Age standardized number in 1990** | **Age standardized number in 2021** |  | **Age standardized number in 1990** | **Age standardized number in 2021** |
| Global | 679549.5 (529615.4 to 852121.5) | 1090591.9 (823498.4 to 1417237.8) |  | 925388.8 (784572.9 to 1079391) | 1209343.3 (1048467.5 to 1377981.6) |
| Sex |  |  |  |  |  |
| Male | 394657.2 (309127.9 to 493514.3) | 632539.5 (479852.2 to 818473.3) |  | 403083.4 (341495.6 to 469459.7) | 537907.5 (466558 to 614722.1) |
| Female | 284892.2 (221082.9 to 360993.3) | 458052.5 (342222.7 to 597970.6) |  | 522305.4 (442931 to 609137.8) | 671435.8 (582294.4 to 763272.6) |
| SDI |  |  |  |  |  |
| High SDI | 94586.2 (75060.7 to 116586) | 108435.3 (84023.6 to 138168.9) |  | 157232.7 (133401.4 to 183208.4) | 154741.6 (134031.1 to 176495.2) |
| High-middle SDI | 186965.1 (145687.6 to 235988) | 222367.3 (167570 to 290094.6) |  | 212135.5 (179562.7 to 247922.3) | 191252.2 (164901.5 to 218821.9) |
| Middle SDI | 226893.8 (173919.4 to 288799) | 370617.3 (276151.4 to 488026.7) |  | 307809.1 (257728.8 to 362670.2) | 375147.1 (319809.7 to 433013.3) |
| Low-middle SDI | 127920.1 (100133.6 to 160874.3) | 277570.8 (209069.3 to 361274.6) |  | 167428.7 (142413.8 to 194532.9) | 305249.4 (266104.1 to 347398.3) |
| Low SDI | 42400.7 (33371.1 to 52653) | 110665 (83621.9 to 143093.1) |  | 79965.6 (69817.7 to 91130.6) | 182038.5 (162453.1 to 203313.9) |
| World Bank Region |  |  |  |  |  |
| East Asia & Pacific | 241119.9 (183159.9 to 310619.2) | 308172.3 (227751.3 to 408839) |  | 344493.1 (287769.6 to 407197.9) | 340218.2 (288317 to 393913) |
| Europe & Central Asia | 138251.8 (111777.8 to 168998.3) | 134839.5 (104918.8 to 170668.7) |  | 145577.2 (126484.9 to 166517.4) | 117448.5 (104333.3 to 131699.4) |
| Latin America & Caribbean | 51000.5 (40436.8 to 62555.8) | 85784.9 (65764.5 to 108770.2) |  | 64086.4 (54466.2 to 74646.4) | 78810.5 (68699.1 to 89627.5) |
| Middle East & North Africa | 42273.2 (34939.8 to 50946.1) | 119558.1 (95029.9 to 149281.9) |  | 63335.5 (56433.1 to 70893.1) | 118717.4 (108437.2 to 129527.8) |
| North America | 32063.4 (24009 to 42223.8) | 30824.4 (22425.6 to 41116) |  | 70352.3 (57847.6 to 84057.8) | 71105.9 (59630 to 83150) |
| South Asia | 134030 (101278.8 to 174547.1) | 302102.3 (220243.3 to 402564.1) |  | 134513.7 (109932.8 to 161548.9) | 251310.4 (210427 to 296899) |
| Sub-Saharan Africa | 39596.2 (31262.4 to 48916.4) | 107889.4 (81986.7 to 137896) |  | 101794.0 (88862.5 to 115986.4) | 230447.8 (205957 to 257162.2) |
| World Bank Income |  |  |  |  |  |
| World Bank High Income | 107417.5 (86228.5 to 131069.1) | 113889.6 (89152.6 to 143194.5) |  | 174059.3 (148537.2 to 202028.8) | 162877.6 (142177.7 to 184988.6) |
| World Bank Upper Middle Income | 318728.9 (244634.1 to 406994.6) | 409216.4 (305960.7 to 536045.5) |  | 378805.4 (318149.9 to 445816.7) | 363605.8 (310318.3 to 417765.3) |
| World Bank Lower Middle Income | 226804.6 (175509.8 to 287856) | 499317.6 (372181.2 to 655871.1) |  | 314694.8 (264917.9 to 368658.7) | 559856.3 (483016.5 to 643290.5) |
| World Bank Low Income | 25812.5 (21197.6 to 30918.6) | 67229.9 (52324.7 to 84548.4) |  | 57007.5 (51149.4 to 63459.4) | 122085.3 (111224.7 to 133984.8) |
| Health System |  |  |  |  |  |
| Advanced Health System | 180929.9 (144317.7 to 223045.1) | 189072.7 (147371.6 to 240386.7) |  | 238024.1 (203323.3 to 275751.4) | 215013.4 (187912.2 to 243485.6) |
| Basic Health System | 318016.7 (245613.6 to 402875.8) | 474826 (358709.3 to 615048.4) |  | 443255.6 (373148.8 to 520279) | 493638.7 (424577.8 to 564209.5) |
| Limited Health System | 170456 (130825.4 to 217778.7) | 398628.3 (294808 to 526745.8) |  | 218950 (184382.4 to 256640.6) | 440482.5 (379997.7 to 506466.2) |
| Minimal Health System | 9363.3 (7631.4 to 11265) | 27128.6 (21140.1 to 33878.8) |  | 24341.9 (21913.6 to 26988.3) | 59294.2 (54177.6 to 65028.4) |
|  | **Ischemic heart disease (Mortality number, 95% UI)** | |  | **Ischemic stroke (Mortality number, 95% UI)** | |
|  | **Age standardized number in 1990** | **Age standardized number in 2021** |  | **Age standardized number in 1990** | **Age standardized number in 2021** |
| Global | 30996.9 (29153 to 32976.6) | 39243.8 (36609.7 to 41781.0) |  | 2725.1 (2457.9 to 3048.0) | 2955.2 (2602.1 to 3382.1) |
| Sex |  |  |  |  |  |
| Male | 20165.8 (18718.5 to 21694.3) | 27513.4 (25496.1 to 29604.5) |  | 1372.0 (1172.8 to 1673.0) | 1708.4 (1458.1 to 1977.3) |
| Female | 10831.1 (9433.3 to 12269.1) | 11730.4 (10554.9 to 13057.7) |  | 1353.2 (1160.4 to 1563.2) | 1246.8 (1051.3 to 1523.4) |
| SDI |  |  |  |  |  |
| High SDI | 2828.5 (2736.4 to 2929.9) | 1850.3 (1649.3 to 2120.7) |  | 276.3 (258.6 to 295.3) | 180.8 (150.3 to 223.3) |
| High-middle SDI | 6407.5 (5956.2 to 6859.8) | 4741.0 (4329.5 to 5241.3) |  | 726.8 (655.3 to 808.0) | 503.9 (444.7 to 569.3) |
| Middle SDI | 10841.3 (10185.2 to 11512.6) | 13846.6 (12804.6 to 14939.6) |  | 983.4 (882.2 to 1102.2) | 1031.7 (913.0 to 1156.6) |
| Low-middle SDI | 8929.0 (7951.5 to 10006.1) | 14421.2 (13072.1 to 15771.8) |  | 571.8 (453.9 to 711.3) | 857.5 (693.5 to 1097.0) |
| Low SDI | 1957.2 (1619.0 to 2349.9) | 4355.3 (3811.3 to 4947.0) |  | 163.8 (122.4 to 223.7) | 378.6 (291.0 to 513.6) |
| World Bank Region |  |  |  |  |  |
| East Asia & Pacific | 9569.9 (8682.8 to 10485.6) | 10445.4 (9305.2 to 11711.3) |  | 1052.8 (915.0 to 1216.7) | 1029.6 (855.3 to 1217.2) |
| Europe & Central Asia | 4865.8 (4701.4 to 5034.9) | 2458.1 (2292.9 to 2637.9) |  | 495.0 (464.7 to 527.3) | 248.6 (228.7 to 270.3) |
| Latin America & Caribbean | 1947.8 (1875.2 to 2018.3) | 2336.3 (2179.3 to 2501.4) |  | 228.3 (216.5 to 240.2) | 157.3 (143.4 to 172.4) |
| Middle East & North Africa | 2994.5 (2678.6 to 3351.5) | 4177.7 (3541.6 to 4895.3) |  | 353.6 (282.0 to 453.6) | 536.8 (432.8 to 656.4) |
| North America | 855.5 (829.9 to 880.7) | 615.9 (573.9 to 650.9) |  | 49.0 (46.9 to 51.2) | 36.9 (34.5 to 39.5) |
| South Asia | 9296.7 (8121.7 to 10571.5) | 16047.8 (14416.8 to 17616.5) |  | 332.9 (237.2 to 465.4) | 500.7 (362.0 to 779.3) |
| Sub-Saharan Africa | 1422.1 (1232.8 to 1637.1) | 3128.5 (2642.7 to 3623.1) |  | 209.0 (166.8 to 276.4) | 442.1 (341.6 to 567.6) |
| World Bank Income |  |  |  |  |  |
| World Bank High Income | 3352.5 (3264.1 to 3450.4) | 1922.6 (1725.1 to 2185.9) |  | 326.6 (309.2 to 345.5) | 171.9 (142.2 to 212.3) |
| World Bank Upper Middle Income | 10522.9 (9710.1 to 11353.6) | 9341.9 (8440.2 to 10394.4) |  | 1228.1 (1095.8 to 1410.1) | 997.8 (873.6 to 1131.3) |
| World Bank Lower Middle Income | 15681.9 (14295.6 to 17173.7) | 25298.7 (23288.8 to 27265.3) |  | 1005.5 (834.3 to 1203.4) | 1445.5 (1212.5 to 1752.3) |
| World Bank Low Income | 1405.9 (1162.3 to 1692.5) | 2651.1 (2169.7 to 3209.0) |  | 161.9 (124.3 to 213.4) | 337.3 (248.2 to 463.7) |
| Health System |  |  |  |  |  |
| Advanced Health System | 5709 (5532.9 to 5890.9) | 3333.6 (3076.0 to 3635.8) |  | 574.8 (543.2 to 610.5) | 327.3 (291.8 to 373.3) |
| Basic Health System | 13724.2 (12825.7 to 14733.8) | 15610.4 (14143.7 to 17066.4) |  | 1545.5 (1387.5 to 1726.1) | 1580.7 (1364.3 to 1808.9) |
| Limited Health System | 11224.1 (9999.2 to 12633.6) | 19371.2 (17617.3 to 21087.8) |  | 551.4 (430.6 to 735.5) | 901.9 (716.3 to 1247.2) |
| Minimal Health System | 306.2 (242.0 to 394.3) | 899.2 (700.5 to 1128.6) |  | 50.5 (36.1 to 71.0) | 142.7 (101.2 to 204.8) |
|  | **Ischemic heart disease (Dalys number, 95% UI)** | |  | **Ischemic stroke (Dalys number, 95% UI)** | |
|  | **Age standardized number in 1990** | **Age standardized number in 2021** |  | **Age standardized number in 1990** | **Age standardized number in 2021** |
| Global | 1833339.9 (1723603.8 to 1951330.5) | 2307599.5 (2153805.4 to 2458762.2) |  | 303562.4 (256019.7 to 355663.3) | 351005.2 (293885.7 to 413950.2) |
| Sex |  |  |  |  |  |
| Male | 1180308 (1094191.7 to 1272198.5) | 1605257.7 (1486540.6 to 1727586.5) |  | 136828.9 (114302.1 to 161907.5) | 171798.4 (145972.9 to 200964) |
| Female | 653031.9 (568337.6 to 740883.7) | 702341.8 (632365.8 to 780911.6) |  | 166733.6 (138437 to 198612.8) | 179206.8 (146079.7 to 217268.2) |
| SDI |  |  |  |  |  |
| High SDI | 163240.3 (157666.4 to 169134) | 107602.3 (95881.8 to 123388.8) |  | 38601.5 (31657.3 to 46405.6) | 32246.1 (25661.2 to 40212.9) |
| High-middle SDI | 373163.9 (346561.8 to 399571.6) | 274289 (250681.5 to 303028.8) |  | 79529.2 (66987 to 93618.2) | 61777 (51048.5 to 73612.4) |
| Middle SDI | 645519.4 (606078.3 to 686146.7) | 812379.4 (752095.3 to 876380.2) |  | 107656.4 (90352.1 to 126674.2) | 118028.5 (98893.5 to 138963.2) |
| Low-middle SDI | 532318 (473803.4 to 597236.5) | 850635.2 (770712.3 to 930273.7) |  | 57541 (46927.6 to 68957.9) | 92737.5 (75829.6 to 112622.4) |
| Low SDI | 117158.2 (96829.1 to 140929.6) | 260968.9 (228297.1 to 296608.3) |  | 19936.1 (16145.4 to 24651.6) | 45925.7 (37157.9 to 56350.6) |
| World Bank Region |  |  |  |  |  |
| East Asia & Pacific | 570809.1 (517653.5 to 625158.2) | 611335.7 (545813.1 to 684628.6) |  | 126288.9 (103963.4 to 152187.8) | 122975.4 (100656.7 to 147869.8) |
| Europe & Central Asia | 277939.6 (268308.5 to 287767.5) | 141213 (131789.9 to 151659.9) |  | 51596.5 (44293.4 to 59259.5) | 32533.2 (26894 to 38434.9) |
| Latin America & Caribbean | 115435.5 (111100.6 to 119701.2) | 138956.7 (129659.5 to 148810.3) |  | 19356 (17573.9 to 21340.6) | 16321.2 (14096.8 to 18770.1) |
| Middle East & North Africa | 181509.3 (162313.3 to 202952.2) | 248419.5 (210488.6 to 290785.9) |  | 30011.9 (24827.2 to 36569.6) | 47712.9 (40026 to 56537.8) |
| North America | 48953.1 (47421.4 to 50590.5) | 35757.3 (33307.1 to 37851) |  | 12303.7 (9355.9 to 15679.4) | 11576.5 (8754.3 to 14736.5) |
| South Asia | 550683.7 (480854 to 626815.5) | 940931.6 (845589.8 to 1032724.8) |  | 38456.2 (29504.2 to 48833.2) | 64399.7 (49613.2 to 84920.9) |
| Sub-Saharan Africa | 85430.3 (74090.2 to 98421.3) | 188981.9 (159985.3 to 218816) |  | 25084 (20649.9 to 30471.8) | 55094.3 (45045.5 to 66310) |
| World Bank Income |  |  |  |  |  |
| World Bank High Income | 193247.4 (187682.5 to 198949.8) | 111815.2 (100279.3 to 127092.6) |  | 42977.1 (35541.7 to 51079.8) | 31923.4 (25480.2 to 39645.8) |
| World Bank Upper Middle Income | 620464 (571507 to 669602.1) | 545745.3 (494170.2 to 606722.9) |  | 137673.8 (114688.2 to 163389.9) | 118408.2 (97587.8 to 140626.6) |
| World Bank Lower Middle Income | 932493.6 (849308 to 1022101.2) | 1488405.5 (1369477 to 1604334.7) |  | 105264.8 (86875.3 to 124967.6) | 163739.1 (135202.3 to 195554.5) |
| World Bank Low Income | 85132.9 (70460 to 102464.7) | 159851.9 (130873.4 to 193011.4) |  | 17751.2 (14529.4 to 21768.2) | 36888.3 (29649.3 to 45871.5) |
| Health System |  |  |  |  |  |
| Advanced Health System | 327050.3 (316807.4 to 337968.3) | 191951.2 (176871.9 to 209208.7) |  | 68749.1 (57542.4 to 80762.3) | 49731.5 (40701 to 60370) |
| Basic Health System | 819264.3 (765608.1 to 879516.5) | 919112.6 (833679.7 to 1004861.6) |  | 165906.2 (139675.8 to 194170) | 172329.3 (144148.4 to 202915) |
| Limited Health System | 666614.2 (593363.2 to 751273) | 1140394.6 (1036412 to 1242866) |  | 62565.3 (50451 to 77237.4) | 112644.5 (90795 to 139811.8) |
| Minimal Health System | 18471.1 (14587.5 to 23709.9) | 54416.3 (42543.3 to 68104.1) |  | 6043.7 (4829.6 to 7664.2) | 16009.6 (12602.5 to 20285.3) |

| **Supplementary Table 3. Age standardized incidence rate of ischemic heart disease vs ischemic stroke in youths and young Adults (15-39 years) at country level, 1990-2021, both sexes** | | | | | |
| --- | --- | --- | --- | --- | --- |
|  | **Ischemic heart disease (Incidence rate, 95% UI)** | |  | **Ischemic stroke (Incidence rate, 95% UI)** | |
|  | **Age standardized rate in 1990 (per 100,000)** | **Age standardized rate in 2021 (per 100,000)** |  | **Age standardized rate in 1990 (per 100,000)** | **Age standardized rate in 2021 (per 100,000)** |
| Afghanistan | 59.3 (30.7 to 94.1) | 65.2 (35.2 to 101.6) |  | 22.3 (14 to 33.7) | 21.8 (14.4 to 31.6) |
| Albania | 41.3 (22.2 to 66) | 36.9 (19.5 to 58.6) |  | 13 (7 to 21.6) | 11.9 (6.5 to 19.5) |
| Algeria | 55.9 (30.4 to 87.1) | 56.4 (29.9 to 89.7) |  | 21.7 (13.6 to 33.3) | 18.3 (11.5 to 27.1) |
| American Samoa | 28.6 (14.3 to 46.8) | 30.6 (15.5 to 49.3) |  | 13.8 (7.7 to 22.3) | 13.8 (8 to 21.7) |
| Andorra | 13.3 (6.1 to 22.9) | 11.4 (5 to 19.8) |  | 8.2 (3.7 to 14.6) | 6.9 (3 to 12.6) |
| Angola | 30.1 (15 to 49) | 28.8 (14.2 to 47.1) |  | 16.1 (8.8 to 26.4) | 14.8 (8.1 to 24.2) |
| Antigua and Barbuda | 46.2 (24.8 to 73.3) | 46.9 (25.2 to 73.6) |  | 11.2 (5.7 to 19.2) | 9.9 (4.9 to 17.2) |
| Argentina | 19.9 (9.7 to 33.4) | 18.5 (8.8 to 30.8) |  | 11.2 (5.7 to 19.3) | 9 (4.4 to 15.5) |
| Armenia | 38.7 (20 to 61.7) | 42.6 (22.4 to 68.5) |  | 23.1 (14.1 to 35.6) | 16.8 (9.5 to 26.5) |
| Australia | 19.7 (10 to 32) | 15.7 (7.1 to 27) |  | 10.4 (6.2 to 15.8) | 8.6 (5.1 to 13) |
| Austria | 14.3 (6.7 to 24.5) | 13.3 (6 to 22.7) |  | 10.2 (5.4 to 17.2) | 8.7 (4.1 to 15.2) |
| Azerbaijan | 41.1 (21.5 to 66.3) | 42.3 (22.2 to 67.6) |  | 18.9 (10.6 to 30.5) | 15.1 (8.2 to 24.7) |
| Bahrain | 65.7 (34.6 to 103.3) | 62.6 (33.6 to 97.5) |  | 15.7 (8.8 to 25.2) | 13.2 (7.1 to 21.8) |
| Bangladesh | 34.5 (16.9 to 56.2) | 35.3 (18.2 to 57.5) |  | 11.4 (5.9 to 19) | 11.4 (6.1 to 18.9) |
| Barbados | 45.9 (24.6 to 71.8) | 49 (26.4 to 76.9) |  | 12.2 (6.6 to 20.1) | 10.8 (5.7 to 17.8) |
| Belarus | 48.7 (26.4 to 77.4) | 48 (25.8 to 76) |  | 19.5 (11 to 32) | 16.3 (9 to 26.9) |
| Belgium | 18.9 (10.1 to 29.4) | 12.5 (5.7 to 21.5) |  | 10.4 (5.1 to 17.8) | 6.8 (3.1 to 11.7) |
| Belize | 44.1 (23.5 to 70) | 47.1 (24.7 to 74.1) |  | 9 (4.3 to 15.8) | 9.2 (4.7 to 15.7) |
| Benin | 27.2 (13.4 to 44.5) | 28.7 (14.4 to 47.1) |  | 18.9 (10.7 to 30.5) | 17.2 (9.9 to 27.4) |
| Bermuda | 45.6 (24 to 72.9) | 46.6 (24.6 to 73.6) |  | 11.1 (5.5 to 19.2) | 9.7 (4.6 to 16.7) |
| Bhutan | 36.5 (18.7 to 58.9) | 36.5 (18.6 to 58.7) |  | 9.9 (4.9 to 17.1) | 9.7 (4.7 to 16.9) |
| Bolivia | 29.8 (15.2 to 48.5) | 30.8 (15.7 to 50.2) |  | 11.8 (6.4 to 19.3) | 9.1 (4.5 to 15.5) |
| Bosnia and Herzegovina | 41.3 (22.1 to 65.5) | 41.7 (22.3 to 66.4) |  | 25.5 (16.9 to 37.8) | 24.9 (16.5 to 36.4) |
| Botswana | 30.5 (15.1 to 49.6) | 33.2 (16.7 to 54.6) |  | 14.8 (7.9 to 24.4) | 14.2 (7.8 to 23.3) |
| Brazil | 29.1 (14.9 to 47.2) | 24.6 (12.4 to 39.9) |  | 13.8 (7.6 to 22.6) | 8.2 (4 to 14.1) |
| Brunei | 10.6 (4.5 to 18.7) | 9.9 (4.1 to 17.6) |  | 16.2 (8.7 to 26.5) | 10.4 (5.1 to 17.9) |
| Bulgaria | 38.3 (20.5 to 61) | 39.7 (20.6 to 62.9) |  | 14.9 (8.6 to 23.4) | 16.3 (9.9 to 24.9) |
| Burkina Faso | 25.7 (12.6 to 42.1) | 25.9 (12.8 to 42.4) |  | 16.4 (8.9 to 27.3) | 14.5 (8 to 23.4) |
| Burundi | 25.8 (12.6 to 42.3) | 26.5 (12.9 to 43.4) |  | 20.2 (12 to 31.8) | 14.5 (8.1 to 23.4) |
| Cabo Verde | 27.4 (13.6 to 45) | 29.7 (14.9 to 48.7) |  | 20.6 (12 to 32.6) | 18.6 (10.5 to 30) |
| Cambodia | 17 (7.8 to 28.6) | 16.9 (8 to 28.4) |  | 10.8 (5.5 to 18.7) | 10.5 (5.6 to 17.3) |
| Cameroon | 23.7 (11.6 to 38.7) | 26.6 (13.1 to 43.6) |  | 16.3 (9.1 to 26.8) | 18.1 (10.9 to 28.3) |
| Canada | 22.6 (11 to 37.3) | 18.8 (9.3 to 31.1) |  | 13.4 (6.7 to 22.8) | 11.3 (7.8 to 15.8) |
| Central African Republic | 30.2 (14.8 to 49.6) | 30.7 (15.2 to 50) |  | 14.5 (7.7 to 24.2) | 13.7 (7.6 to 22) |
| Chad | 28.8 (14.3 to 47.4) | 29.4 (14.7 to 47.8) |  | 19.5 (11.1 to 31.2) | 19.1 (11.6 to 29.3) |
| Chile | 9.3 (3.7 to 16.9) | 9.7 (4.3 to 16.7) |  | 11.3 (5.5 to 19.2) | 9.5 (4.5 to 16.4) |
| China | 35.1 (18.6 to 55.2) | 39 (20.8 to 61.6) |  | 9.9 (5.3 to 16.5) | 11.7 (6.4 to 19.2) |
| Colombia | 35.2 (18.6 to 56.7) | 33.8 (17.6 to 54.3) |  | 12.1 (6.4 to 19.9) | 8.1 (3.9 to 13.9) |
| Comoros | 25.9 (12.9 to 42.9) | 27.1 (13.5 to 44.3) |  | 19.4 (11 to 30.8) | 15.8 (8.7 to 25.8) |
| Cook Islands | 26.5 (12.9 to 43.3) | 28.8 (14.6 to 46.7) |  | 13.7 (7.7 to 22.3) | 15.5 (9.1 to 24.3) |
| Costa Rica | 40.5 (21.6 to 64.1) | 39.6 (20.8 to 63.1) |  | 11.5 (5.8 to 19.5) | 9.6 (4.7 to 16.5) |
| Croatia | 34.4 (18.3 to 54.9) | 31.9 (16.3 to 50.3) |  | 12.4 (6.4 to 20.8) | 9.2 (4.6 to 15.8) |
| Cuba | 47.7 (25.6 to 75.7) | 45.4 (23.9 to 71.4) |  | 11.7 (6.1 to 19.8) | 9.8 (4.9 to 16.4) |
| Cyprus | 8.7 (3.6 to 15.8) | 7.8 (3 to 14.6) |  | 6.9 (3 to 12.6) | 5.1 (2 to 9.6) |
| Czechia | 52.8 (29.8 to 81.8) | 49.8 (27.3 to 78.9) |  | 15.9 (8.9 to 26) | 10.8 (5.3 to 18.6) |
| Democratic Republic of the Congo | 29 (14.5 to 47.6) | 28.6 (14.1 to 47.3) |  | 14.5 (7.6 to 24.3) | 12.7 (6.9 to 21.1) |
| Denmark | 5.8 (3.1 to 9.6) | 9.2 (3.8 to 16.3) |  | 7.6 (4.7 to 11.7) | 7.1 (3.1 to 12.5) |
| Djibouti | 27 (13.4 to 44.4) | 28.8 (14.2 to 47.6) |  | 16.6 (9 to 27.3) | 16.3 (9 to 26.3) |
| Dominica | 47.7 (25.4 to 75.3) | 47.2 (24.8 to 73.2) |  | 11.1 (5.9 to 18.9) | 9.6 (5 to 16.4) |
| Dominican Republic | 44 (23.6 to 69.7) | 46.7 (24.8 to 73.2) |  | 12.1 (6.7 to 19.7) | 13.4 (7.9 to 20.7) |
| East Timor | 17.2 (8.1 to 28.6) | 17.1 (8 to 28.5) |  | 10.7 (5.2 to 18.5) | 11.4 (6.2 to 18.8) |
| Ecuador | 29.9 (15.2 to 48.4) | 32.3 (16.6 to 52.4) |  | 13.9 (8 to 22.3) | 11.2 (6.2 to 18.1) |
| Egypt | 51.9 (27.4 to 81.6) | 58.5 (31.4 to 91.1) |  | 19 (12.1 to 28) | 21.7 (14.2 to 31.9) |
| El Salvador | 32.1 (16.3 to 51.5) | 34.1 (17.9 to 54.4) |  | 10.4 (5.3 to 17.5) | 8.1 (3.9 to 14) |
| Equatorial Guinea | 29.4 (14.5 to 47.9) | 30.4 (15 to 49.8) |  | 16.4 (9 to 26.8) | 13.2 (6.7 to 22.1) |
| Eritrea | 25.4 (12.3 to 41.6) | 25.9 (12.7 to 42.6) |  | 16.2 (8.8 to 26.6) | 15.2 (8.5 to 24.4) |
| Estonia | 60.7 (40.1 to 86.8) | 60.1 (32.8 to 94.9) |  | 18 (11.1 to 27.3) | 14 (6.9 to 23.7) |
| eSwatini | 29.3 (14.5 to 47.7) | 31.7 (15.9 to 51.8) |  | 12.7 (6.2 to 22.1) | 12.3 (6.4 to 20.4) |
| Ethiopia | 27.8 (13.5 to 45) | 26.9 (13.2 to 44) |  | 15.2 (8.1 to 25) | 10.3 (5.1 to 17.5) |
| Federated States of Micronesia | 29.6 (14.8 to 48) | 29.8 (14.8 to 48.9) |  | 18.2 (10.9 to 28.2) | 19.6 (12.6 to 29) |
| Fiji | 31.5 (16 to 51) | 32.3 (16.5 to 52.2) |  | 18 (10.8 to 28.2) | 17.8 (11.1 to 27.3) |
| Finland | 30.4 (18.3 to 45.3) | 14.4 (6.7 to 24.4) |  | 11.5 (6.8 to 17.8) | 9.9 (5.5 to 15.8) |
| France | 18.7 (11 to 28.3) | 13.3 (6.3 to 22.9) |  | 6.8 (4.1 to 10.3) | 6.5 (4 to 9.9) |
| Gabon | 27.4 (13.7 to 44.6) | 28.3 (13.9 to 46.1) |  | 15.5 (8.2 to 26) | 14.3 (7.7 to 23.8) |
| Gambia | 29.9 (14.7 to 49) | 31.2 (15.7 to 50.4) |  | 22.4 (13.3 to 35.6) | 21.1 (13 to 32.3) |
| Georgia | 43.3 (23 to 68.5) | 45.7 (24.2 to 73.1) |  | 22.2 (12.9 to 35.3) | 20.4 (12.4 to 31.8) |
| Germany | 24.3 (15.1 to 35.8) | 16.3 (8 to 27.8) |  | 14.5 (8.6 to 22.6) | 10 (5.2 to 16.8) |
| Ghana | 29.1 (14.6 to 47.3) | 30.7 (15.4 to 50.2) |  | 31.9 (20.8 to 47.1) | 32.9 (22.3 to 47.8) |
| Greece | 9.9 (4.2 to 17.8) | 8.7 (3.5 to 15.6) |  | 10 (4.7 to 17.1) | 7.6 (3.5 to 13.3) |
| Greenland | 24.6 (12.2 to 40.2) | 21.2 (10.4 to 34.3) |  | 16.6 (8.8 to 27.5) | 11.3 (5.6 to 19.5) |
| Grenada | 47.6 (25.1 to 75.2) | 48.4 (25.9 to 76.5) |  | 16.1 (9.8 to 24.8) | 11.3 (6.1 to 18.3) |
| Guam | 24.2 (11.8 to 39.9) | 29 (14.6 to 47.2) |  | 11.7 (6.3 to 19.4) | 16.6 (10.1 to 25.8) |
| Guatemala | 29.5 (14.9 to 47.9) | 32.5 (16.6 to 52.4) |  | 10 (5.2 to 16.4) | 8.4 (4.3 to 14) |
| Guinea | 26.1 (12.8 to 42.1) | 28 (13.9 to 45.9) |  | 19.1 (11.3 to 30.5) | 20.7 (12.6 to 31.5) |
| Guinea-Bissau | 28.7 (14.4 to 46.9) | 30.2 (15 to 49.1) |  | 25 (15.6 to 38.3) | 23.3 (14.9 to 34.2) |
| Guyana | 49.6 (26.3 to 78.8) | 48.7 (25.9 to 76.5) |  | 16.4 (10.3 to 24.6) | 13.8 (8.7 to 20.7) |
| Haiti | 49.7 (26.5 to 79.2) | 51.7 (27.5 to 81.7) |  | 12.8 (7.3 to 20.3) | 11.5 (6.9 to 17.6) |
| Honduras | 36.7 (19 to 58.4) | 36.9 (19.4 to 60.1) |  | 10.4 (5.3 to 17.3) | 8.5 (4.1 to 15) |
| Hungary | 52.2 (28.9 to 81.6) | 39.8 (21.4 to 62.6) |  | 22.3 (14.4 to 33) | 12.3 (6.7 to 20.4) |
| Iceland | 18 (9 to 30.2) | 13.8 (6.3 to 23.6) |  | 10.4 (5 to 17.9) | 7.5 (3.3 to 13.4) |
| India | 42 (22.3 to 67.2) | 44.9 (23.9 to 71.2) |  | 11 (5.5 to 18.7) | 10.8 (5.6 to 18.1) |
| Indonesia | 21.3 (10.3 to 35) | 21.8 (10.6 to 35.6) |  | 18.8 (10.9 to 30.3) | 16.3 (9.5 to 25.5) |
| Iran | 59.7 (31.9 to 93.4) | 60.7 (32.5 to 94.8) |  | 23 (14.1 to 35.3) | 17 (10.6 to 26) |
| Iraq | 62.5 (33.9 to 99.3) | 67.7 (36.5 to 106.7) |  | 22.4 (14.1 to 33.7) | 18.4 (11.3 to 28.3) |
| Ireland | 14 (6.3 to 23.9) | 11.1 (4.7 to 19.5) |  | 9.5 (4.5 to 16.4) | 6 (2.5 to 11.2) |
| Israel | 12.7 (5.8 to 21.7) | 11 (4.7 to 19.2) |  | 10.7 (5.1 to 18.3) | 7.6 (3.4 to 13.8) |
| Italy | 19.5 (9.2 to 32.6) | 16.1 (7.7 to 26.8) |  | 10.5 (4.7 to 19.1) | 6 (2.6 to 10.8) |
| Ivory Coast | 29.4 (15 to 48.7) | 31.2 (15.7 to 50.7) |  | 23.3 (14.2 to 35.9) | 20.6 (12.8 to 31.6) |
| Jamaica | 45.4 (24.1 to 72.1) | 48.2 (25.8 to 76.1) |  | 10.5 (5.4 to 17.4) | 9.6 (5 to 16.4) |
| Japan | 9.9 (4 to 17.6) | 9.3 (3.8 to 16.8) |  | 9.9 (4.4 to 17.8) | 10.4 (5 to 18.1) |
| Jordan | 61.2 (33 to 95.7) | 64.5 (34.2 to 101.4) |  | 22.7 (13.6 to 36.4) | 18 (10.2 to 29.2) |
| Kazakhstan | 46.7 (25.1 to 74.7) | 47.7 (25.7 to 76.5) |  | 30.5 (19.8 to 44.8) | 22.2 (13.8 to 34.5) |
| Kenya | 30 (15 to 48.7) | 30.5 (15.2 to 49.5) |  | 15.4 (8.1 to 25.8) | 13.6 (7.3 to 22.7) |
| Kiribati | 32.3 (16.5 to 52.4) | 32.3 (16.5 to 52.1) |  | 27 (18.3 to 39.4) | 28.1 (19.9 to 39.3) |
| Kuwait | 67.4 (36.2 to 106.4) | 64.9 (34.5 to 101.6) |  | 18.2 (10.4 to 29.8) | 17.4 (9.7 to 28.6) |
| Kyrgyzstan | 40.7 (21.3 to 64.9) | 36.8 (19.3 to 59) |  | 25 (15.7 to 38.3) | 15.2 (8.7 to 24) |
| Laos | 19.4 (9.1 to 32.1) | 17.9 (8.5 to 29.8) |  | 13.7 (7.5 to 22.4) | 13.1 (7.5 to 21) |
| Latvia | 35.6 (18.5 to 57) | 34.4 (18 to 54.5) |  | 17 (9.5 to 27.4) | 12.9 (6.8 to 21.6) |
| Lebanon | 53.3 (28.1 to 85.7) | 61.9 (32.7 to 96.8) |  | 19.6 (12.1 to 30.2) | 20.5 (13 to 31) |
| Lesotho | 26.8 (13.1 to 44.2) | 29.5 (14.7 to 48.1) |  | 11.4 (5.5 to 19.9) | 10.9 (5.7 to 18) |
| Liberia | 29 (14.6 to 47.7) | 30.8 (15.4 to 50.1) |  | 21 (12.4 to 33.4) | 18.9 (11.3 to 29.9) |
| Libya | 56.4 (30.3 to 89.1) | 61.2 (32.3 to 95.5) |  | 17.4 (10.8 to 26.4) | 22 (14.8 to 31.6) |
| Lithuania | 39.5 (21.7 to 60.8) | 34.3 (18 to 54.4) |  | 20.4 (11.6 to 32.5) | 16.2 (8.8 to 26.7) |
| Luxembourg | 9.6 (4 to 17) | 8.6 (3.4 to 15.6) |  | 10.4 (5.4 to 17.6) | 6 (2.6 to 10.8) |
| Madagascar | 26.1 (12.8 to 42.9) | 27.6 (13.6 to 45.5) |  | 23.7 (14.6 to 36.5) | 21.7 (13.4 to 33.2) |
| Malawi | 28.5 (14.2 to 46.8) | 29.9 (15.1 to 48.7) |  | 15.8 (8.6 to 26.3) | 15.2 (8.6 to 24.5) |
| Malaysia | 19.7 (9.4 to 32.8) | 21.6 (10.6 to 35.6) |  | 15.1 (8.2 to 24.9) | 14.8 (8.2 to 24) |
| Maldives | 18.9 (8.9 to 31.4) | 18.8 (9 to 31.2) |  | 18.9 (11.2 to 29.6) | 11.2 (5.7 to 18.9) |
| Mali | 26.9 (13.2 to 43.9) | 27.9 (13.7 to 45.5) |  | 18.9 (10.9 to 30.3) | 16 (9.2 to 25.8) |
| Malta | 10.2 (4.2 to 17.7) | 8.7 (3.5 to 15.7) |  | 9.5 (4.5 to 16.5) | 6.3 (2.9 to 11.4) |
| Marshall Islands | 27.6 (13.8 to 45.2) | 28.7 (14.2 to 47.3) |  | 16.7 (10.2 to 25.6) | 19.9 (13.1 to 29.2) |
| Mauritania | 30.1 (15.1 to 49.4) | 30.8 (15.5 to 50.3) |  | 26.5 (16.1 to 41.4) | 19.1 (11.1 to 30.7) |
| Mauritius | 19.1 (9.1 to 32) | 18.4 (8.8 to 30.6) |  | 17.1 (10.1 to 27) | 11.6 (6.2 to 19.4) |
| Mexico | 40.8 (21.4 to 64.7) | 40.8 (21.7 to 64.6) |  | 15.1 (8.3 to 24.7) | 10.5 (5.4 to 17.7) |
| Moldova | 42.6 (22.8 to 68.9) | 45.6 (24.3 to 73) |  | 15.9 (8.7 to 25.9) | 15.1 (8.5 to 24.5) |
| Monaco | 12.7 (5.8 to 21.7) | 11.1 (4.9 to 19.4) |  | 10.6 (5.3 to 18.1) | 8.6 (4.2 to 15) |
| Mongolia | 47 (24.8 to 75.9) | 46.4 (24.6 to 75.1) |  | 17.5 (9.9 to 28.6) | 19.3 (11.5 to 30.7) |
| Montenegro | 46.1 (24.5 to 73.1) | 45.5 (24.8 to 72.5) |  | 11.5 (5.7 to 19.8) | 11.2 (5.5 to 19.2) |
| Morocco | 58.7 (31.4 to 92.7) | 59.3 (31.8 to 92.9) |  | 19.1 (11.7 to 29.4) | 18 (11.3 to 27.3) |
| Mozambique | 28.8 (14.4 to 47.1) | 29.8 (15.1 to 48.7) |  | 15.7 (8.3 to 25.8) | 17.1 (10.2 to 27.2) |
| Myanmar | 19.5 (9.3 to 32.1) | 17.6 (8.4 to 29.3) |  | 14 (7.7 to 22.6) | 12.8 (7.2 to 20.6) |
| Namibia | 31.2 (15.6 to 51) | 29.6 (14.9 to 48.5) |  | 14.7 (7.6 to 24.7) | 12.2 (6.2 to 20.6) |
| Nauru | 29.5 (14.9 to 48.1) | 31.9 (16.4 to 52) |  | 26.1 (16.9 to 38.9) | 30.2 (21.1 to 42.7) |
| Nepal | 35.9 (18.6 to 57.7) | 33.8 (17.2 to 54.5) |  | 9.9 (4.9 to 17.2) | 8.8 (4.1 to 15.3) |
| Netherlands | 17.7 (8.7 to 30) | 14 (6.5 to 23.8) |  | 12.1 (6.1 to 20.7) | 8.3 (3.9 to 14.9) |
| New Zealand | 29.8 (14.6 to 49.8) | 17 (9.2 to 27.2) |  | 9.8 (4.5 to 17.6) | 8.1 (3.8 to 14.4) |
| Nicaragua | 35.2 (18.3 to 56.8) | 34.4 (17.9 to 55.2) |  | 11.2 (5.7 to 19.1) | 8.4 (4 to 14.6) |
| Niger | 26.8 (13.2 to 43.3) | 27.8 (13.6 to 45.3) |  | 19.7 (11.3 to 31.1) | 17 (9.8 to 27) |
| Nigeria | 30.2 (15.2 to 49.2) | 32.9 (16.8 to 53) |  | 17.1 (9.3 to 28.4) | 16.4 (9.2 to 26.6) |
| Niue | 28.6 (14.6 to 46.1) | 29.6 (15 to 47.8) |  | 18.6 (11.3 to 28.7) | 17 (10.4 to 25.7) |
| North Korea | 34.6 (18 to 55) | 35.3 (18.3 to 56.5) |  | 14 (7.9 to 22.3) | 15 (9 to 23.3) |
| North Macedonia | 43.6 (23.5 to 69.2) | 42 (22.3 to 66.8) |  | 17.9 (10.6 to 28) | 14.5 (8.4 to 23) |
| Northern Mariana Islands | 27.9 (13.7 to 46.1) | 30.1 (15.2 to 48.9) |  | 12.3 (6.5 to 20.7) | 11.6 (6.2 to 19.1) |
| Norway | 18 (8.8 to 29.9) | 14.4 (6.6 to 24.4) |  | 13.1 (6.1 to 23.3) | 7.2 (3.2 to 12.8) |
| Oman | 52.5 (28 to 83.1) | 62.4 (33.5 to 97.7) |  | 18.8 (11.7 to 28.8) | 21.2 (13.8 to 31.3) |
| Pakistan | 48.7 (25.9 to 76.3) | 52.7 (28.5 to 82.1) |  | 14 (7.5 to 23.3) | 15.9 (9.1 to 25.4) |
| Palau | 26.1 (12.9 to 42.6) | 30.5 (15.3 to 49.6) |  | 18.6 (11.4 to 28.4) | 22.9 (15.1 to 33.7) |
| Palestine | 57.7 (30.4 to 90.6) | 57.6 (30.5 to 91.2) |  | 16.7 (9.8 to 26.8) | 14.5 (8.5 to 23.4) |
| Panama | 34.3 (17.7 to 55.7) | 35.3 (18.4 to 57.2) |  | 10.1 (5.1 to 17.2) | 8.2 (3.9 to 14.2) |
| Papua New Guinea | 27.1 (13.4 to 44) | 28.1 (13.8 to 45.6) |  | 8.8 (4.1 to 15.7) | 8 (3.7 to 14.1) |
| Paraguay | 24.1 (12 to 40) | 24.5 (12.3 to 40.1) |  | 9.3 (4.4 to 16.1) | 8.2 (3.8 to 14.5) |
| Peru | 28.3 (14.2 to 45.8) | 31.5 (16.2 to 51.4) |  | 10.9 (5.7 to 18.2) | 10.1 (5.1 to 17) |
| Philippines | 19.6 (9.4 to 32.4) | 18.9 (9.1 to 31.3) |  | 10.6 (5.1 to 18.4) | 14.2 (8.2 to 22.5) |
| Poland | 53.1 (27.1 to 86.3) | 30.3 (15.7 to 48.3) |  | 13.8 (7.4 to 22.8) | 8.8 (4.3 to 15.1) |
| Portugal | 8 (3.1 to 14.6) | 7.5 (2.8 to 13.7) |  | 10.4 (5.6 to 17.2) | 5.4 (2.2 to 10) |
| Puerto Rico | 48.2 (25.4 to 75.6) | 49.2 (26.1 to 76.9) |  | 9.5 (4.6 to 16.5) | 8.8 (4.1 to 15.6) |
| Qatar | 63.5 (33.4 to 100.1) | 61.4 (32.5 to 97.1) |  | 19.8 (11.9 to 30.4) | 15.7 (8.4 to 26.2) |
| Republic of Serbia | 60.5 (35.1 to 92.2) | 59.9 (32.9 to 93.5) |  | 15.8 (9.1 to 25.1) | 11.5 (6.2 to 18.9) |
| Republic of the Congo | 30 (14.9 to 48.9) | 30.4 (15.3 to 49.5) |  | 18.4 (10.4 to 29.9) | 15.4 (8.5 to 24.9) |
| Romania | 42.7 (22.8 to 67.6) | 42 (22.5 to 66.6) |  | 15.4 (8.6 to 24.8) | 11.9 (6.4 to 19.7) |
| Russia | 76 (42.3 to 118.2) | 81.9 (45.7 to 127.1) |  | 20.3 (11.8 to 32.1) | 16.9 (9.9 to 26.1) |
| Rwanda | 24.6 (12.1 to 40.8) | 24.8 (11.9 to 41) |  | 19.9 (11.5 to 31.7) | 13.1 (6.9 to 22) |
| Saint Kitts and Nevis | 48.2 (25.3 to 75.8) | 47.6 (25.4 to 74.7) |  | 22.9 (15 to 33.3) | 10.9 (5.8 to 18.1) |
| Saint Lucia | 50.6 (26.9 to 80.7) | 48.7 (26 to 77.3) |  | 14.6 (8.3 to 23.4) | 10.6 (5.6 to 17.6) |
| Saint Vincent and the Grenadines | 46.6 (24.9 to 73.9) | 45.7 (24.5 to 72.8) |  | 16.5 (10.1 to 25) | 10.3 (5.8 to 16.9) |
| Samoa | 30.4 (15.3 to 49.4) | 31.2 (15.7 to 50.3) |  | 15.3 (8.5 to 24.6) | 18.8 (11.9 to 28.3) |
| San Marino | 12.7 (5.8 to 21.8) | 11.2 (4.9 to 19.8) |  | 8.6 (4 to 15) | 7.6 (3.5 to 13.5) |
| São Tomé and Principe | 27.8 (13.8 to 45.3) | 29.3 (14.6 to 48) |  | 25.3 (15.5 to 39.6) | 29.1 (18.6 to 42.6) |
| Saudi Arabia | 56.2 (29.9 to 88) | 67.5 (36.6 to 106.4) |  | 15 (8.7 to 23.7) | 16 (10.1 to 24) |
| Senegal | 31.8 (16.2 to 50.9) | 32 (16.4 to 51.7) |  | 24.9 (14.8 to 39.3) | 21 (12.4 to 32.4) |
| Seychelles | 18.3 (8.7 to 31) | 18.9 (9 to 31.4) |  | 16.3 (9.4 to 25.6) | 14.2 (8.1 to 22.2) |
| Sierra Leone | 31.6 (16.2 to 50.9) | 30.3 (15.3 to 49.8) |  | 23.2 (13.8 to 36.3) | 22.1 (14 to 33.5) |
| Singapore | 16.6 (7.6 to 28.3) | 12.9 (5.8 to 22.4) |  | 13.8 (7 to 23.7) | 9 (4 to 16.2) |
| Slovakia | 40.9 (21.8 to 65.2) | 35.6 (18.8 to 55.8) |  | 16.5 (9.4 to 26.6) | 13.3 (7.2 to 21.6) |
| Slovenia | 42.4 (23.2 to 67) | 38.8 (20.7 to 61.1) |  | 11.5 (5.8 to 19.9) | 7.9 (3.6 to 14) |
| Solomon Islands | 29.1 (14.7 to 48) | 30.4 (15.2 to 49.3) |  | 16.5 (9.8 to 25.6) | 18.9 (12 to 27.9) |
| Somalia | 28.1 (13.9 to 46.4) | 29.5 (14.8 to 47.7) |  | 17.3 (9.7 to 28.5) | 15.8 (9 to 25.5) |
| South Africa | 41.6 (21.9 to 66.6) | 39.1 (20.5 to 62.6) |  | 20.9 (12.1 to 33.2) | 14 (7.8 to 22.7) |
| South Korea | 9.2 (3.7 to 16.3) | 7.7 (2.8 to 14.3) |  | 17.5 (9.6 to 28.2) | 11.6 (5.6 to 20.1) |
| South Sudan | 25.2 (12.3 to 41.3) | 26.5 (13 to 43.5) |  | 14.7 (7.7 to 24.5) | 14 (7.3 to 23.8) |
| Spain | 15 (7.5 to 24.5) | 12 (5.3 to 20.8) |  | 9.8 (5.2 to 16.3) | 6.4 (3.4 to 10.6) |
| Sri Lanka | 18.2 (8.6 to 30.5) | 17.8 (8.4 to 29.5) |  | 13.7 (7.5 to 22.4) | 12.6 (6.9 to 20.7) |
| Sudan | 59.8 (32 to 94.4) | 64 (34.8 to 99.6) |  | 18.4 (11.4 to 28.1) | 19.7 (12.8 to 28.7) |
| Suriname | 50 (26.4 to 79.6) | 50.5 (26.8 to 79.9) |  | 12.6 (7.1 to 20.4) | 11.6 (6.6 to 18.3) |
| Sweden | 12.1 (8.6 to 16) | 14.8 (6.6 to 25.3) |  | 9.2 (4.4 to 16.3) | 10.3 (4.9 to 18) |
| Switzerland | 14.9 (8 to 23.7) | 10.2 (4.4 to 18) |  | 8.5 (4.1 to 14.7) | 5.6 (2.3 to 10.3) |
| Syria | 55 (28.9 to 86.8) | 54.9 (29.7 to 86.8) |  | 27.5 (18.7 to 39.2) | 20.2 (13.3 to 29.3) |
| Tajikistan | 41.6 (21.8 to 66.4) | 41.4 (21.7 to 66.2) |  | 18.8 (10.6 to 30.1) | 15.1 (8.4 to 24.1) |
| Thailand | 16.8 (7.9 to 27.9) | 16.5 (7.8 to 27.3) |  | 12.6 (6.5 to 21.1) | 12.4 (7 to 19.9) |
| The Bahamas | 48.5 (25.8 to 76.5) | 50.3 (27.1 to 79.6) |  | 11.8 (6.3 to 19.2) | 10.8 (6 to 17.6) |
| Togo | 29.1 (14.6 to 47.7) | 29.3 (14.7 to 47.8) |  | 22.9 (13.7 to 36.4) | 21.1 (13.1 to 32) |
| Tokelau | 24.5 (12.1 to 40) | 28.3 (14 to 46.3) |  | 13.4 (7.9 to 21) | 15.6 (9.6 to 23.8) |
| Tonga | 29.1 (14.5 to 47.9) | 30.4 (15.4 to 48.8) |  | 11.9 (6.2 to 20.2) | 12.3 (6.8 to 20) |
| Trinidad and Tobago | 54.8 (29.4 to 87.6) | 55.4 (29.7 to 87.1) |  | 18.7 (11.9 to 28.2) | 13.1 (7.4 to 21) |
| Tunisia | 51.8 (27.9 to 82.9) | 54.1 (28.6 to 85.9) |  | 14 (7.7 to 22.7) | 15.4 (9.1 to 24.2) |
| Turkey | 58.6 (31.5 to 92.5) | 51.9 (27.6 to 82.3) |  | 20.2 (12.1 to 32.2) | 14.4 (7.9 to 23.8) |
| Turkmenistan | 41.2 (21.5 to 65.5) | 46.3 (24.8 to 73.4) |  | 20.9 (12.4 to 32.4) | 29.1 (19.6 to 42) |
| Tuvalu | 26.4 (13.1 to 43.5) | 29.6 (14.7 to 48.5) |  | 15.5 (9.5 to 24) | 18.1 (11.5 to 26.6) |
| Uganda | 26.2 (12.9 to 42.4) | 25.7 (12.5 to 42.3) |  | 16 (8.4 to 27.2) | 14.2 (7.5 to 23.4) |
| Ukraine | 51.4 (27.8 to 80.1) | 53 (28.9 to 82.7) |  | 21.4 (12.1 to 34.8) | 22.1 (13.6 to 34) |
| United Arab Emirates | 66.4 (34.8 to 105.2) | 66.1 (34.9 to 105.2) |  | 24.3 (15.1 to 37.6) | 22.2 (14.1 to 33) |
| United Kingdom | 16.9 (7.7 to 29.2) | 15.4 (7.3 to 25.8) |  | 9.2 (4.4 to 15.9) | 6.8 (3.4 to 11.6) |
| United Republic of Tanzania | 25.1 (12.4 to 41.4) | 29.4 (14.8 to 48) |  | 14.8 (7.7 to 24.9) | 17.1 (9.7 to 27.5) |
| United States of America | 30.8 (14 to 52.8) | 21.9 (11 to 35.4) |  | 12 (5.8 to 20.9) | 11.2 (5.6 to 19.2) |
| United States Virgin Islands | 44.3 (23.3 to 70.1) | 47.1 (25.2 to 74.5) |  | 9.8 (4.8 to 16.9) | 9.2 (4.5 to 16.1) |
| Uruguay | 13.2 (5.9 to 22.4) | 12.9 (5.8 to 22.3) |  | 17.4 (9.9 to 27.6) | 9.1 (4.4 to 15.6) |
| Uzbekistan | 38.5 (20.3 to 61.6) | 42.9 (22.8 to 68.5) |  | 22.5 (13.4 to 34.9) | 20 (12.2 to 30.6) |
| Vanuatu | 32 (16.2 to 52) | 34.2 (17.5 to 54.8) |  | 19.7 (11.9 to 30.2) | 24.2 (15.9 to 35.1) |
| Venezuela | 39.7 (21 to 63.4) | 37.3 (19.5 to 59.4) |  | 10.4 (5 to 17.9) | 9 (4.4 to 15.5) |
| Vietnam | 17.2 (8.1 to 28.6) | 17.4 (8.1 to 28.9) |  | 12.3 (6.3 to 20.8) | 13.1 (7.3 to 21.1) |
| Yemen | 57.4 (30.8 to 91.6) | 58.5 (31.1 to 91.2) |  | 16.1 (9.5 to 25.1) | 16.6 (10.5 to 24.9) |
| Zambia | 26.4 (12.7 to 43.5) | 26.4 (12.9 to 42.8) |  | 15.4 (8.4 to 25.2) | 13.9 (7.7 to 22.5) |
| Zimbabwe | 30.6 (15.2 to 50.2) | 33.1 (16.6 to 53.9) |  | 12.8 (6.8 to 21.1) | 14.8 (8.5 to 23.6) |

| **Supplementary Table 4. Age standardized prevalence rate of ischemic heart disease vs ischemic stroke in youths and young Adults (15-39 years) at country level, 1990-2021, both sexes** | | | | | |
| --- | --- | --- | --- | --- | --- |
|  | **Ischemic heart disease (Prevalence rate, 95% UI)** | |  | **Ischemic stroke (Prevalence rate, 95% UI)** | |
|  | **Age standardized rate in 1990 (per 100,000)** | **Age standardized rate in 2021 (per 100,000)** |  | **Age standardized rate in 1990 (per 100,000)** | **Age standardized rate in 2021 (per 100,000)** |
| Afghanistan | 244.8 (204.4 to 288.8) | 285.4 (228.5 to 354.4) |  | 341.9 (308.4 to 376.4) | 301.9 (277.7 to 327.4) |
| Albania | 253.6 (214.2 to 298.9) | 232 (184.8 to 285.8) |  | 214.1 (192.8 to 237.2) | 195.3 (177.8 to 215.1) |
| Algeria | 265.9 (225 to 314.7) | 276.2 (219 to 345.2) |  | 333.9 (302.1 to 367.6) | 285.3 (261.6 to 309.7) |
| American Samoa | 156.2 (128.1 to 186.5) | 166.4 (131.6 to 205.9) |  | 277.4 (249.3 to 306.6) | 255.9 (233.8 to 281.2) |
| Andorra | 89.2 (70.6 to 111) | 85.7 (66.1 to 107.1) |  | 137.3 (119.9 to 156.3) | 126.4 (111.4 to 143) |
| Angola | 122.4 (99.6 to 147) | 120.6 (94.8 to 151.7) |  | 256.2 (229.7 to 284.9) | 231.6 (210.9 to 255.3) |
| Antigua and Barbuda | 236.4 (198.8 to 281.2) | 249.1 (198.8 to 304.9) |  | 188.8 (168.8 to 210.4) | 174.8 (157.4 to 192.8) |
| Argentina | 96.7 (78.5 to 117.9) | 90.3 (70.4 to 113) |  | 163.1 (143.8 to 184.3) | 139.5 (124.5 to 155.8) |
| Armenia | 209.4 (176.8 to 246.2) | 234.2 (186.7 to 286.8) |  | 265.2 (238.3 to 294.4) | 224 (203.4 to 247.9) |
| Australia | 98.9 (81.1 to 119) | 84.8 (65.8 to 108.3) |  | 145 (129.7 to 160.9) | 137.7 (125.8 to 150.1) |
| Austria | 95.2 (75.5 to 118.4) | 104.5 (82.1 to 131.4) |  | 192.3 (169.4 to 217.2) | 160.8 (142.4 to 181.5) |
| Azerbaijan | 214.1 (181.6 to 254.7) | 222.5 (175.9 to 274.1) |  | 225.7 (201 to 252.7) | 202.7 (182.9 to 224.6) |
| Bahrain | 333.9 (282 to 394.3) | 322.3 (258.6 to 397.3) |  | 304.5 (273.3 to 339.9) | 245.2 (222.3 to 269.9) |
| Bangladesh | 164.3 (135.8 to 196.6) | 179.5 (141.9 to 220.6) |  | 153.4 (135.3 to 173) | 154.1 (138.2 to 170.6) |
| Barbados | 230.7 (193.8 to 272.5) | 256.4 (203.6 to 314.6) |  | 197.7 (173.5 to 223.6) | 188.8 (170.8 to 208.4) |
| Belarus | 261.4 (220.7 to 307.3) | 259.5 (206.3 to 320.4) |  | 244.1 (215.8 to 275.6) | 230 (208.7 to 253.6) |
| Belgium | 97.8 (80.1 to 118.3) | 87.7 (67.3 to 111.9) |  | 158.5 (133.9 to 185) | 126.3 (110.5 to 142.4) |
| Belize | 204.8 (173 to 243.6) | 229.3 (182.7 to 282.9) |  | 158.3 (140.2 to 177) | 156.6 (141.6 to 173.3) |
| Benin | 123.5 (101.1 to 148.7) | 137.9 (107.3 to 173.6) |  | 326.3 (295.9 to 359.7) | 296.8 (272 to 324.2) |
| Bermuda | 241.8 (204 to 287.8) | 255.1 (205.6 to 312.2) |  | 185.1 (163.7 to 208.2) | 171.6 (154.4 to 190.7) |
| Bhutan | 172.7 (143.2 to 207.2) | 183.2 (145.5 to 227.7) |  | 148.6 (130.7 to 168.9) | 142.1 (126.5 to 158.7) |
| Bolivia | 146.6 (120.4 to 176.3) | 154.2 (121.1 to 192.3) |  | 183.1 (164 to 203.7) | 148.3 (133.5 to 164.9) |
| Bosnia and Herzegovina | 257.5 (216.7 to 304.2) | 277.9 (221.7 to 339.3) |  | 320.8 (292.1 to 352.5) | 340.5 (314.6 to 367.8) |
| Botswana | 126.1 (103.1 to 151.7) | 147.3 (113.4 to 183.2) |  | 242.5 (215.2 to 270.5) | 234.5 (213.4 to 258) |
| Brazil | 169.6 (128.4 to 217.3) | 162.6 (120.5 to 213.1) |  | 169.6 (137.1 to 206.1) | 128.9 (106.6 to 153.2) |
| Brunei | 79.7 (63.2 to 98.6) | 72.9 (56.1 to 92.5) |  | 195.3 (173.1 to 221) | 150.4 (133.9 to 167.2) |
| Bulgaria | 238.4 (200 to 281.9) | 259.3 (205.4 to 318.9) |  | 222 (192.2 to 254.8) | 226.8 (198.2 to 257.4) |
| Burkina Faso | 115.6 (93.4 to 139.5) | 122.5 (95.2 to 150.9) |  | 272.6 (244.6 to 301.9) | 243.5 (222.5 to 268.1) |
| Burundi | 113.1 (91.5 to 136.5) | 119.1 (92.8 to 148.8) |  | 268.5 (242.7 to 298.1) | 216.5 (196.9 to 238.5) |
| Cabo Verde | 134.2 (110.2 to 160.1) | 156.1 (122.3 to 193.1) |  | 345.2 (312.4 to 382.3) | 317.8 (291.1 to 346.4) |
| Cambodia | 97 (78.4 to 118.3) | 103.5 (80.4 to 130.2) |  | 173.1 (153.4 to 194) | 162.3 (146.1 to 179.9) |
| Cameroon | 103.4 (83.5 to 125.1) | 121.5 (95 to 150.5) |  | 295.7 (267.2 to 327.1) | 298.1 (273.5 to 323.3) |
| Canada | 114.7 (92.9 to 140.7) | 98.8 (77.2 to 124.1) |  | 234.7 (210.8 to 262) | 213.8 (195.3 to 233) |
| Central African Republic | 109.3 (88.8 to 131.1) | 114.2 (87.6 to 144.9) |  | 235.4 (211 to 262.1) | 219 (199.1 to 241.1) |
| Chad | 132.4 (108 to 159.3) | 143.1 (111 to 179.3) |  | 336 (303.9 to 371.5) | 316.6 (291.5 to 342.7) |
| Chile | 78.4 (61.6 to 97.5) | 86.3 (66.7 to 109) |  | 164.1 (144.9 to 185.4) | 155 (138.4 to 172.1) |
| China | 194.5 (145 to 255.8) | 211 (152.1 to 286.4) |  | 214.3 (176.6 to 255.6) | 200.6 (166.3 to 236.6) |
| Colombia | 167.6 (139.5 to 200.1) | 163.3 (129.7 to 204.4) |  | 185.7 (165.7 to 207.6) | 140.8 (126.3 to 157.1) |
| Comoros | 129.9 (105.2 to 156.3) | 140.4 (108.6 to 173.7) |  | 270.8 (243.9 to 301.5) | 232.1 (210.6 to 255.5) |
| Cook Islands | 145.6 (120.6 to 174.5) | 162.2 (128.2 to 201.3) |  | 263.2 (238.1 to 291.3) | 272.2 (248.2 to 298.1) |
| Costa Rica | 211.1 (176.6 to 250.9) | 214.5 (170.6 to 264.8) |  | 190.9 (168.7 to 214.7) | 167.8 (150.7 to 186.6) |
| Croatia | 244 (203.3 to 288.5) | 244 (193.1 to 300.2) |  | 176.9 (149.2 to 206.7) | 149.3 (133.2 to 165.8) |
| Cuba | 242.9 (204.7 to 289.4) | 235.2 (186.5 to 291.7) |  | 184.9 (163.9 to 207.3) | 166.4 (150.4 to 183.9) |
| Cyprus | 81.5 (63.7 to 102.7) | 82.1 (63.3 to 103.8) |  | 118.2 (100.6 to 137.3) | 105.4 (89.8 to 121.8) |
| Czechia | 320.7 (272.5 to 376.7) | 295.1 (235.6 to 360) |  | 211.5 (184.9 to 239.8) | 167.9 (150.5 to 187) |
| Democratic Republic of the Congo | 116.6 (94.8 to 140.1) | 114.8 (88.7 to 144.6) |  | 230.9 (205.9 to 257.9) | 195.6 (175.8 to 217.1) |
| Denmark | 81.3 (62.4 to 107.1) | 75.6 (58.4 to 96.6) |  | 154.5 (135.5 to 174.8) | 122.7 (108.2 to 138.2) |
| Djibouti | 131.9 (107 to 160.5) | 147.9 (114.5 to 185.5) |  | 254.5 (228.1 to 283.9) | 248.8 (226.5 to 274) |
| Dominica | 229.3 (191.5 to 273.3) | 233.9 (185.3 to 288.1) |  | 180.3 (160.6 to 201.6) | 161.5 (145.5 to 178.4) |
| Dominican Republic | 209.5 (175 to 248.1) | 231.4 (184.7 to 287.5) |  | 192.1 (172.8 to 213.5) | 190.8 (173.5 to 209.2) |
| East Timor | 103.3 (83.9 to 126.4) | 111 (87.2 to 140.5) |  | 174.2 (154.3 to 196.1) | 174.6 (158 to 192) |
| Ecuador | 148.8 (123.1 to 179) | 164.5 (129.7 to 206) |  | 202.8 (182 to 226.6) | 176.3 (159.8 to 193.4) |
| Egypt | 239.1 (200.6 to 284.1) | 286.7 (228.9 to 358.2) |  | 299.8 (267.7 to 335.6) | 315.6 (290.1 to 341.9) |
| El Salvador | 148.6 (122.3 to 179.1) | 161.2 (127.4 to 201.7) |  | 170.9 (152.1 to 191) | 141.8 (126.9 to 157.8) |
| Equatorial Guinea | 116.2 (95.5 to 139.1) | 132.7 (103.5 to 166.1) |  | 250.8 (225.4 to 279.2) | 227.6 (205.8 to 251.3) |
| Eritrea | 110.4 (89.3 to 133.4) | 118.2 (91.8 to 147.3) |  | 227.5 (203.1 to 254.4) | 212.9 (193.1 to 233.4) |
| Estonia | 302.2 (260.4 to 349.2) | 293.8 (234.1 to 360.9) |  | 203.4 (173.7 to 236.9) | 180 (161.3 to 200.2) |
| eSwatini | 119.4 (96.7 to 145.3) | 137.6 (107.2 to 173.8) |  | 212.4 (188.8 to 238.1) | 204.8 (185.7 to 225.9) |
| Ethiopia | 118.2 (87.4 to 155) | 121.3 (86.2 to 164.3) |  | 213.2 (171.9 to 258.7) | 165.1 (136.9 to 196.7) |
| Federated States of Micronesia | 145.2 (119.5 to 174.2) | 146.8 (114.7 to 183.3) |  | 304.6 (275.6 to 335.4) | 289 (266.1 to 313.1) |
| Fiji | 172.4 (143.3 to 206.1) | 178.1 (141.5 to 219.1) |  | 333.3 (301.4 to 367.6) | 311.6 (286.5 to 338.9) |
| Finland | 140.7 (112.2 to 174.2) | 96.4 (74.4 to 122.5) |  | 180.5 (161.8 to 201.2) | 167.5 (149.6 to 186.8) |
| France | 101.9 (83.5 to 123.7) | 86.3 (66.6 to 108.4) |  | 120.7 (104.6 to 137.4) | 97.9 (88.3 to 108.6) |
| Gabon | 115.5 (94 to 138.3) | 122.4 (95 to 154.2) |  | 252.1 (225.5 to 282.1) | 236.1 (214.4 to 260.1) |
| Gambia | 141 (115 to 170.6) | 154.8 (119.6 to 193.1) |  | 386.2 (351.8 to 423.3) | 339.7 (312.3 to 369.1) |
| Georgia | 231.6 (195.4 to 274.3) | 243.5 (193.3 to 300.3) |  | 313.7 (280.2 to 350.2) | 279.8 (254.2 to 307.7) |
| Germany | 131.3 (108.5 to 158) | 104.8 (81.4 to 130.9) |  | 213.7 (190.3 to 239.2) | 175.6 (155.3 to 196.8) |
| Ghana | 129.2 (105 to 155) | 147.9 (114.6 to 183.7) |  | 471.5 (431.7 to 513.3) | 479.6 (444.6 to 515.7) |
| Greece | 84.3 (65.5 to 106.1) | 78.9 (60.8 to 100.6) |  | 173.7 (150.4 to 199.2) | 139.4 (123 to 157.6) |
| Greenland | 104.4 (85.8 to 126.1) | 101.4 (79.2 to 128.2) |  | 303.5 (272.6 to 336.7) | 221 (199.4 to 244.2) |
| Grenada | 230.6 (193.9 to 273) | 246.9 (197.3 to 305.1) |  | 220.3 (192 to 252) | 189.7 (165 to 216.2) |
| Guam | 129 (106.4 to 155.3) | 158.1 (124.9 to 196.8) |  | 255.3 (229.8 to 283.5) | 304 (278.1 to 330) |
| Guatemala | 132.3 (105.3 to 161.7) | 143.2 (112.9 to 179.8) |  | 159.7 (143 to 179.2) | 141.7 (127.2 to 157.2) |
| Guinea | 114 (93.4 to 136.6) | 129.4 (100.4 to 161.4) |  | 320.4 (290.9 to 354.1) | 322.4 (296.4 to 349.8) |
| Guinea-Bissau | 121.9 (98.9 to 147.2) | 136.5 (106.5 to 169.6) |  | 400.2 (365.9 to 437.4) | 356.6 (329.4 to 385.2) |
| Guyana | 218.8 (182.4 to 258) | 228.1 (182.2 to 281.4) |  | 253.5 (228.4 to 281.8) | 204.7 (187.2 to 223.5) |
| Haiti | 214.2 (178.6 to 256) | 233.6 (184.2 to 288.1) |  | 193.7 (175.2 to 214.1) | 176.4 (161.2 to 192.7) |
| Honduras | 170.9 (142 to 202.7) | 177.6 (140.8 to 218.9) |  | 186.1 (165.7 to 208) | 154.4 (137.8 to 172.1) |
| Hungary | 346.7 (294.4 to 407.2) | 271.7 (216.7 to 332.8) |  | 254.2 (229.3 to 282.4) | 192.9 (173.5 to 213.7) |
| Iceland | 102.9 (82.5 to 127) | 90.9 (70.1 to 115) |  | 165.5 (144.3 to 188.8) | 140 (124.2 to 158.1) |
| India | 183.4 (136.4 to 243) | 205.1 (146.3 to 277) |  | 156.6 (125.6 to 189.9) | 151.4 (124.9 to 180.9) |
| Indonesia | 126.5 (94.9 to 164.2) | 135.5 (98.8 to 181) |  | 290.7 (237.2 to 351.2) | 241.1 (199.9 to 286.5) |
| Iran | 272.4 (203.8 to 358.5) | 290.4 (207.8 to 395.8) |  | 401 (330.7 to 476.5) | 303.9 (255.4 to 356.9) |
| Iraq | 294.5 (249 to 349.1) | 339.7 (271.5 to 416.1) |  | 372.8 (340.3 to 408.3) | 338.1 (310.2 to 367) |
| Ireland | 98.6 (79.2 to 122.2) | 84.2 (65 to 106.5) |  | 149 (128 to 171.4) | 112 (97.7 to 127.5) |
| Israel | 101.9 (80.1 to 128.3) | 91.7 (70.3 to 115.4) |  | 148.5 (130.5 to 167.9) | 126 (111.1 to 141.6) |
| Italy | 118.6 (91 to 151.4) | 102.6 (75.5 to 136.4) |  | 172.9 (139.2 to 211.9) | 129.4 (106.6 to 153.9) |
| Ivory Coast | 132.9 (108.4 to 159.6) | 148.5 (115.8 to 186.4) |  | 406 (370.5 to 441.7) | 351.1 (323.8 to 381.2) |
| Jamaica | 229.7 (191.3 to 274.5) | 252.8 (201.2 to 309.9) |  | 183 (162.2 to 205.5) | 163.9 (147.9 to 181.3) |
| Japan | 82.4 (61.7 to 107.1) | 70.6 (51.9 to 92.8) |  | 153.2 (123 to 186.9) | 152 (125.6 to 180.3) |
| Jordan | 306.8 (259.4 to 364.8) | 332.4 (265.7 to 411.9) |  | 384.9 (346.7 to 424.2) | 329.2 (298.9 to 359.8) |
| Kazakhstan | 239.7 (202.2 to 283.3) | 251 (199.3 to 308) |  | 405.6 (370.2 to 443.6) | 304.6 (277.8 to 333.4) |
| Kenya | 145.1 (108.8 to 188) | 151.9 (111.3 to 201.4) |  | 261.4 (212.3 to 315.9) | 227.3 (189 to 269.7) |
| Kiribati | 160.1 (131.2 to 191) | 158.9 (125.1 to 196.7) |  | 415.5 (382.6 to 452) | 376 (348.5 to 403.4) |
| Kuwait | 356.4 (299.9 to 422.3) | 355 (286.4 to 438.2) |  | 348.5 (315.1 to 384.2) | 320.7 (292.2 to 351.5) |
| Kyrgyzstan | 204.2 (171.6 to 243.4) | 176.1 (137.1 to 219.1) |  | 292.5 (263.8 to 323.5) | 201.6 (183.3 to 222.3) |
| Laos | 109.9 (90 to 132.7) | 109.7 (84.5 to 138.4) |  | 208.5 (185.8 to 233.8) | 198 (179.7 to 217.9) |
| Latvia | 217.3 (182.3 to 258.5) | 216.5 (170.8 to 270.2) |  | 218 (185.2 to 252.5) | 183.1 (163.9 to 203.8) |
| Lebanon | 259.6 (218.8 to 309.8) | 318.8 (254.9 to 391.9) |  | 317.6 (287.6 to 350) | 345.8 (316.7 to 375.5) |
| Lesotho | 97.4 (78.8 to 118) | 114.7 (89 to 145.2) |  | 186 (164.3 to 209.6) | 175.7 (158.3 to 193.9) |
| Liberia | 132.6 (108.2 to 160.1) | 147.7 (114.8 to 185.1) |  | 346.1 (313.9 to 382.7) | 300.3 (275 to 327) |
| Libya | 276.4 (231 to 330.1) | 304 (241.5 to 380.4) |  | 363.5 (331.9 to 397) | 374.2 (346.9 to 402.1) |
| Lithuania | 215.4 (182.7 to 254.3) | 203.9 (160.8 to 252.7) |  | 258.5 (206 to 318.3) | 224.5 (188.2 to 264.7) |
| Luxembourg | 83 (64.7 to 104) | 81.7 (63 to 103.6) |  | 158.3 (128.8 to 192.9) | 119.8 (105.5 to 135.7) |
| Madagascar | 122.1 (99.2 to 147.9) | 134.3 (103.4 to 167.3) |  | 303.2 (274.4 to 334.5) | 284.9 (260.5 to 311.6) |
| Malawi | 127.5 (103.6 to 154.1) | 142.4 (111 to 177.7) |  | 258.5 (232.3 to 288.6) | 243.5 (222 to 267.9) |
| Malaysia | 135.2 (110.8 to 163.1) | 162.9 (128.6 to 203.5) |  | 219.3 (195.5 to 246.1) | 228.4 (207.2 to 251.1) |
| Maldives | 128.9 (106.2 to 156.1) | 135.5 (107.1 to 170.1) |  | 241.9 (217.2 to 270) | 170.2 (153.5 to 188) |
| Mali | 114 (92.9 to 136.4) | 125.5 (97.2 to 158.5) |  | 308.9 (277.5 to 343.1) | 279.7 (255.8 to 305.4) |
| Malta | 91.9 (71.9 to 115.8) | 85.4 (65.5 to 109) |  | 148.3 (125.9 to 172.1) | 120.4 (105.5 to 136.7) |
| Marshall Islands | 135 (110.6 to 162.4) | 138.7 (108.6 to 172.6) |  | 288.7 (261.5 to 319) | 281.1 (258.3 to 305.4) |
| Mauritania | 146.6 (120.7 to 175.7) | 159.3 (124.8 to 197.3) |  | 397.4 (361.4 to 437.5) | 316 (288.4 to 346.8) |
| Mauritius | 132.7 (108.1 to 161.5) | 134.2 (105.3 to 167.8) |  | 253.3 (228.2 to 281.6) | 187 (169.4 to 207.2) |
| Mexico | 200.2 (150.9 to 260.8) | 204.3 (147.8 to 275.7) |  | 245.6 (199.6 to 296.9) | 190.9 (158 to 225.4) |
| Moldova | 224.4 (188.3 to 267.2) | 239 (187.2 to 295.4) |  | 204.1 (180.8 to 230.7) | 201.2 (182.4 to 221.8) |
| Monaco | 85.2 (67.2 to 107.4) | 85.1 (65.8 to 107.4) |  | 163.3 (142.2 to 186) | 150.2 (133.3 to 168.7) |
| Mongolia | 230.1 (191.9 to 272.5) | 233.4 (184.2 to 288.2) |  | 311.7 (283.3 to 340.7) | 293.3 (267.8 to 319.1) |
| Montenegro | 302.3 (255.7 to 354.5) | 314.5 (250.1 to 383.3) |  | 173.7 (153.5 to 195) | 174.2 (156.4 to 193.4) |
| Morocco | 288.3 (240.6 to 344.8) | 302.2 (240.7 to 372.5) |  | 331.6 (300.8 to 366.1) | 305.4 (279.4 to 333.1) |
| Mozambique | 123 (99.3 to 150.8) | 137.4 (106.2 to 171.1) |  | 253.3 (226.7 to 283.3) | 259.5 (236.5 to 284.1) |
| Myanmar | 106.2 (87 to 128) | 103.2 (80.1 to 130.8) |  | 218.9 (195.3 to 243.8) | 200.7 (182.8 to 219.5) |
| Namibia | 135.7 (111.6 to 163.5) | 131.8 (102.6 to 164.7) |  | 233.4 (206.6 to 262.2) | 199 (179.2 to 220.2) |
| Nauru | 150.2 (123.8 to 180.9) | 163.4 (128.7 to 203.4) |  | 453.3 (415.9 to 491.9) | 467.4 (436.1 to 501.2) |
| Nepal | 165.4 (137.7 to 197.6) | 162 (128 to 203) |  | 143.6 (126.5 to 162.8) | 130.6 (116 to 145.8) |
| Netherlands | 116.3 (94 to 142.9) | 93.2 (71.4 to 116.7) |  | 196.2 (172.4 to 221.3) | 151.6 (133.7 to 170.3) |
| New Zealand | 147.3 (109.4 to 194.5) | 102 (72.3 to 138.9) |  | 169.1 (135.9 to 206.7) | 156.4 (128.8 to 185.6) |
| Nicaragua | 173 (144.8 to 206) | 171.1 (135 to 214) |  | 177.8 (157.8 to 201.2) | 147.9 (132.1 to 164.4) |
| Niger | 118.7 (95.8 to 142.6) | 128.8 (100.6 to 161.6) |  | 319.2 (289.7 to 351.9) | 278.1 (253.3 to 305.4) |
| Nigeria | 139.6 (103.3 to 182.3) | 160.5 (115.8 to 215.3) |  | 311.5 (254.4 to 372.9) | 301.8 (251.4 to 355) |
| Niue | 154.3 (127.2 to 185.1) | 160.1 (125.8 to 200.2) |  | 340 (308.7 to 373.6) | 337.1 (309.9 to 364.1) |
| North Korea | 175.7 (146.5 to 209.5) | 180.7 (142.3 to 224.4) |  | 260.2 (234.5 to 287.6) | 238.8 (218 to 261.2) |
| North Macedonia | 283.9 (238.2 to 336.1) | 283.5 (224.9 to 348.8) |  | 266.9 (227.4 to 310.2) | 227.4 (193.2 to 264.1) |
| Northern Mariana Islands | 158.9 (131.4 to 190.7) | 170.4 (134.3 to 211.3) |  | 251.8 (224.8 to 280.4) | 239 (217 to 263.8) |
| Norway | 110.6 (84.4 to 142.8) | 92.6 (67.2 to 123.4) |  | 202.6 (163.7 to 249.4) | 147.6 (121.8 to 175.3) |
| Oman | 244.1 (205.4 to 289.5) | 309.2 (248.8 to 381.1) |  | 342.2 (311.3 to 374.7) | 401.5 (370.7 to 431.9) |
| Pakistan | 226.4 (170.3 to 294.4) | 242.9 (175.8 to 327.3) |  | 207.4 (168.1 to 251.6) | 211.7 (175.4 to 254.4) |
| Palau | 134.2 (109.8 to 161.6) | 157.6 (124.5 to 195.4) |  | 322.1 (292.6 to 355) | 333.4 (306.6 to 360.7) |
| Palestine | 282.9 (238.3 to 336.9) | 291.7 (232.7 to 360.5) |  | 294 (264 to 326.4) | 260.4 (236.3 to 285.8) |
| Panama | 166.7 (139.7 to 198.9) | 179 (142.3 to 222.7) |  | 184.6 (164.9 to 206.1) | 151.2 (135.5 to 167.9) |
| Papua New Guinea | 135.7 (111.9 to 163.7) | 140.6 (109.3 to 176.9) |  | 202.3 (179.9 to 226.3) | 187.2 (169 to 205.9) |
| Paraguay | 167.1 (136.6 to 201.9) | 176.5 (140.1 to 218.8) |  | 137.5 (121.2 to 155.7) | 122.3 (109 to 136.9) |
| Peru | 145.3 (119.5 to 174.9) | 166.6 (131.3 to 208.7) |  | 175.8 (157.4 to 196.3) | 162.5 (146.5 to 179.6) |
| Philippines | 124.7 (94.2 to 161) | 131.1 (96.9 to 173.4) |  | 193.2 (156.4 to 232.6) | 208.2 (172.7 to 246.7) |
| Poland | 261.3 (199.6 to 337.2) | 232 (171.5 to 307.2) |  | 181.1 (147 to 218.8) | 148.9 (123.4 to 177) |
| Portugal | 68.4 (53.2 to 86.4) | 73.4 (55.3 to 95) |  | 151.2 (125.5 to 180) | 108.2 (94.3 to 123.7) |
| Puerto Rico | 244.7 (207.1 to 290.7) | 260.4 (209.3 to 320) |  | 172.9 (153.2 to 193.5) | 166.7 (149.5 to 185.4) |
| Qatar | 327 (276.8 to 386.8) | 313.9 (251.9 to 387) |  | 356.5 (323.5 to 391.5) | 292.3 (263.7 to 321.6) |
| Republic of Serbia | 360.7 (306.8 to 425.1) | 364.8 (292.3 to 442.9) |  | 234.7 (200.1 to 273.7) | 189.6 (161.6 to 220.3) |
| Republic of the Congo | 120.3 (98.1 to 144.3) | 127.7 (99 to 160.8) |  | 276.7 (247.3 to 307.3) | 246.1 (223.6 to 270.4) |
| Romania | 271.9 (229.1 to 319.7) | 270.5 (214.9 to 330.7) |  | 218.3 (188.8 to 249.3) | 184.1 (165 to 204.7) |
| Russia | 366.2 (274 to 479.6) | 397.1 (286 to 536.1) |  | 259.9 (212.5 to 313.8) | 225.6 (188.5 to 267.3) |
| Rwanda | 108.9 (88.5 to 132.3) | 113.8 (87.9 to 143.2) |  | 263.6 (236.6 to 293) | 203.5 (184.3 to 224.3) |
| Saint Kitts and Nevis | 234.9 (195.9 to 279.3) | 241 (192 to 296.7) |  | 270.8 (236.2 to 310) | 187.1 (169.6 to 205.9) |
| Saint Lucia | 250.3 (210.4 to 295.7) | 247.4 (196.7 to 304.3) |  | 224.1 (193.7 to 256.7) | 182.2 (164.7 to 201.5) |
| Saint Vincent and the Grenadines | 218.9 (183.2 to 260.5) | 223.8 (178.3 to 277.5) |  | 230.7 (204.5 to 258.6) | 170.2 (154.6 to 187.5) |
| Samoa | 163.2 (135 to 195.4) | 168.2 (133.5 to 209.7) |  | 298.2 (268.6 to 330.1) | 302.6 (277.8 to 328.9) |
| San Marino | 85.1 (67.7 to 106.4) | 85.9 (66.2 to 108.5) |  | 145.1 (126.4 to 165.6) | 136.9 (121.1 to 154.5) |
| São Tomé and Principe | 137.3 (112.6 to 165.4) | 153.7 (120.2 to 191.5) |  | 420.4 (383.1 to 460.6) | 425.7 (392.9 to 459.2) |
| Saudi Arabia | 263.4 (223.4 to 314.4) | 336.5 (268.9 to 411.7) |  | 263.7 (237.3 to 292.3) | 272.9 (250.3 to 297.1) |
| Senegal | 154.1 (125.9 to 185.4) | 162.6 (127.2 to 204) |  | 405.2 (367.7 to 446.5) | 349.7 (321 to 381.7) |
| Seychelles | 119.7 (97.1 to 145.4) | 129.2 (101.8 to 162.6) |  | 225.9 (202.4 to 251.3) | 209.2 (190.5 to 229.7) |
| Sierra Leone | 145.5 (119.3 to 173.7) | 145.3 (114.8 to 181.8) |  | 398.7 (363.8 to 438.4) | 357.5 (330.6 to 386.2) |
| Singapore | 103 (82.2 to 126.2) | 82.4 (66.8 to 100.8) |  | 174.8 (154.1 to 197.4) | 133.1 (118.2 to 149.5) |
| Slovakia | 264 (221.9 to 311.2) | 240.4 (191.2 to 295.5) |  | 219.6 (196.2 to 246.1) | 198.7 (179.6 to 219.8) |
| Slovenia | 286.7 (241.1 to 339.9) | 266.1 (211.1 to 327.5) |  | 167.3 (139.9 to 197.9) | 126.2 (111.5 to 141.4) |
| Solomon Islands | 139.5 (115.2 to 167.7) | 147.1 (115.2 to 184.8) |  | 301.9 (273.4 to 331.9) | 297.7 (273.4 to 322.9) |
| Somalia | 121.4 (98.3 to 147.2) | 132.9 (102.5 to 166) |  | 248 (222.9 to 276.6) | 232.3 (211.2 to 255.8) |
| South Africa | 167.1 (124 to 218.9) | 163.1 (116.5 to 219.8) |  | 328.7 (266.4 to 395.9) | 240.9 (199.5 to 284.2) |
| South Korea | 65.6 (51.3 to 81.8) | 67 (51.3 to 85.3) |  | 226.8 (202.2 to 252.7) | 170.8 (152.8 to 189.7) |
| South Sudan | 116.9 (94.4 to 140.9) | 126.5 (97.8 to 157.9) |  | 226.1 (201.6 to 254.7) | 217.6 (196.9 to 240.1) |
| Spain | 90.4 (73 to 111.1) | 80.3 (61.5 to 101) |  | 144.8 (123.7 to 167.4) | 110.8 (99.1 to 124) |
| Sri Lanka | 116.2 (94.7 to 141.6) | 124 (97.2 to 156.2) |  | 228.7 (204.6 to 255.6) | 199.1 (179.6 to 220.4) |
| Sudan | 267.2 (224.2 to 317.1) | 307.4 (245 to 383.5) |  | 320.3 (290.1 to 353.6) | 323.8 (298.4 to 350.6) |
| Suriname | 241.2 (202.3 to 287.9) | 255.7 (203.9 to 313.1) |  | 210.1 (189.5 to 232.5) | 185.6 (169.2 to 203.5) |
| Sweden | 84.3 (63.3 to 109.2) | 84.3 (61.8 to 111.8) |  | 168.3 (135.4 to 206.5) | 221.5 (182.4 to 264.1) |
| Switzerland | 89.9 (72.4 to 111.9) | 75.8 (57.6 to 96.7) |  | 135.3 (116.3 to 155.7) | 103.9 (91.3 to 118.4) |
| Syria | 269.7 (224.4 to 321.4) | 276.8 (220.2 to 347.7) |  | 466.1 (429.7 to 507) | 353.4 (325.1 to 382.9) |
| Tajikistan | 216.9 (181.4 to 258.5) | 206.5 (161.1 to 256.8) |  | 227.8 (203.1 to 254.8) | 202.9 (183.7 to 224.4) |
| Thailand | 102 (82.4 to 123.7) | 106.3 (83.5 to 133.2) |  | 194.5 (173.6 to 218.2) | 181.3 (164.1 to 200.3) |
| The Bahamas | 235 (197 to 279.9) | 253.5 (201.1 to 314.5) |  | 194.5 (173.7 to 216.4) | 184.5 (166.9 to 203.1) |
| Togo | 130.9 (106.4 to 157.8) | 137.8 (107 to 173.2) |  | 378.5 (344.7 to 416.2) | 328 (302.7 to 354) |
| Tokelau | 124.6 (101.8 to 149.9) | 146.9 (115.1 to 183.9) |  | 249.4 (224.6 to 275.4) | 261.1 (238.3 to 285.6) |
| Tonga | 162.5 (133.7 to 195.5) | 170.6 (135.4 to 213.3) |  | 256.1 (229.3 to 284.5) | 249.8 (227.2 to 274) |
| Trinidad and Tobago | 278.2 (235.3 to 328.9) | 298.7 (237.4 to 364.9) |  | 273.5 (247.3 to 302.4) | 221 (201.6 to 242.5) |
| Tunisia | 248.9 (208.4 to 296.4) | 265.5 (212 to 326.3) |  | 243.8 (218.7 to 272) | 250.8 (229.1 to 275.6) |
| Turkey | 281.8 (238.4 to 333.8) | 252.5 (200.7 to 313.7) |  | 365.9 (328.8 to 405) | 269.8 (244.4 to 297) |
| Turkmenistan | 205.6 (173.1 to 243.9) | 237.3 (187.8 to 293.2) |  | 290.6 (263.2 to 319.4) | 354.9 (328.3 to 381.8) |
| Tuvalu | 130.9 (108.1 to 157.2) | 148.4 (116.6 to 185.5) |  | 275 (248.8 to 302.6) | 263.4 (240.8 to 286) |
| Uganda | 113.5 (91.5 to 137.2) | 115.7 (89.1 to 146) |  | 266.1 (238.7 to 297.7) | 231.8 (210.9 to 255.4) |
| Ukraine | 262.7 (197.1 to 343.5) | 263.1 (188.1 to 355.5) |  | 279.5 (227.1 to 338.1) | 273.6 (228 to 325.9) |
| United Arab Emirates | 322 (271.5 to 380.6) | 332 (267.3 to 407.6) |  | 429.6 (389 to 472.5) | 351.9 (321.8 to 383.6) |
| United Kingdom | 126.1 (96.8 to 160) | 98.5 (73.2 to 128.2) |  | 180.7 (149.7 to 214.8) | 134.8 (113.5 to 158) |
| United Republic of Tanzania | 107.5 (87.3 to 129.4) | 137 (105.8 to 171.8) |  | 236.8 (211.1 to 265.5) | 273.6 (250.4 to 299.6) |
| United States of America | 140.2 (103.2 to 186.9) | 126.6 (91.5 to 170.5) |  | 315.2 (257 to 379.9) | 295.5 (245.7 to 348.5) |
| United States Virgin Islands | 218.6 (183.5 to 259.3) | 243.2 (194 to 296.5) |  | 168.6 (149 to 190.5) | 161.4 (145.1 to 179.7) |
| Uruguay | 83.8 (66.5 to 103.9) | 80.7 (62.7 to 101.9) |  | 206.4 (184.5 to 229.4) | 143.9 (128.6 to 160) |
| Uzbekistan | 197.5 (162.4 to 238.7) | 221.8 (175.5 to 273.6) |  | 257.1 (228.8 to 287.6) | 248.2 (225.4 to 272.5) |
| Vanuatu | 164.7 (135.5 to 196.6) | 178.1 (140.7 to 221.5) |  | 363.2 (328.9 to 399.3) | 376.2 (347.4 to 407.2) |
| Venezuela | 194.6 (162.9 to 230.7) | 187.7 (148.9 to 231.9) |  | 179.7 (158.9 to 202.2) | 160.1 (143.7 to 177.3) |
| Vietnam | 107.3 (87.4 to 130.1) | 117.2 (91.4 to 147) |  | 185.5 (163.7 to 209.3) | 185.8 (167.5 to 205.1) |
| Yemen | 251.7 (211.7 to 300.9) | 265.2 (211.6 to 328.9) |  | 268.6 (241.8 to 297.7) | 250.1 (229 to 271.5) |
| Zambia | 108.5 (87.8 to 131.3) | 113.8 (87.3 to 142.6) |  | 227.3 (203.5 to 252.9) | 210.6 (190.8 to 232.3) |
| Zimbabwe | 128.8 (103.8 to 157.1) | 142.9 (112 to 177.9) |  | 218.9 (194.9 to 244.2) | 230.5 (208.6 to 253) |

| **Supplementary Table 5. Age standardized mortality rate of ischemic heart disease vs ischemic stroke in youths and young Adults (15-39 years) at country level, 1990-2021, both sexes** | | | | | |
| --- | --- | --- | --- | --- | --- |
|  | **Ischemic heart disease (Mortality rate, 95% UI)** | |  | **Ischemic stroke (Mortality rate, 95% UI)** | |
|  | **Age standardized rate in 1990 (per 100,000)** | **Age standardized rate in 2021 (per 100,000)** |  | **Age standardized rate in 1990 (per 100,000)** | **Age standardized rate in 2021 (per 100,000)** |
| Afghanistan | 20.3 (13 to 29.7) | 16.3 (10.8 to 24.5) |  | 2.4 (1.3 to 4.1) | 2.4 (1.3 to 4.3) |
| Albania | 4.6 (3.6 to 5.9) | 3.4 (2.5 to 4.5) |  | 0.2 (0.1 to 0.3) | 0.1 (0.1 to 0.2) |
| Algeria | 14.7 (10.6 to 19.9) | 6.9 (4.8 to 9.5) |  | 1.6 (1 to 2.5) | 1 (0.6 to 1.6) |
| American Samoa | 11.8 (8.4 to 16.2) | 14.4 (9.9 to 19.4) |  | 0.7 (0.4 to 1) | 0.6 (0.4 to 0.9) |
| Andorra | 2 (1.4 to 3) | 0.9 (0.5 to 1.3) |  | 0.2 (0.1 to 0.3) | 0.1 (0 to 0.1) |
| Angola | 3.4 (2.1 to 5) | 3.5 (2.2 to 5.2) |  | 0.4 (0.3 to 0.7) | 0.4 (0.2 to 0.6) |
| Antigua and Barbuda | 4.6 (3.9 to 5.4) | 1 (0.7 to 1.2) |  | 0.5 (0.4 to 0.6) | 0.2 (0.1 to 0.2) |
| Argentina | 6.2 (5.4 to 7) | 2.1 (1.8 to 2.4) |  | 0.4 (0.3 to 0.5) | 0.1 (0.1 to 0.1) |
| Armenia | 9.7 (8.5 to 10.9) | 5.1 (4.4 to 5.9) |  | 0.7 (0.5 to 0.9) | 0.3 (0.2 to 0.3) |
| Australia | 3 (2.6 to 3.4) | 1.1 (0.9 to 1.3) |  | 0.2 (0.1 to 0.2) | 0 (0 to 0) |
| Austria | 3.3 (2.9 to 3.8) | 0.9 (0.8 to 1) |  | 0.4 (0.3 to 0.5) | 0 (0 to 0.1) |
| Azerbaijan | 15.5 (13 to 18.2) | 7.3 (5.6 to 9.1) |  | 0.6 (0.4 to 0.9) | 0.2 (0.1 to 0.4) |
| Bahrain | 11.7 (9.4 to 14.2) | 5.9 (4.4 to 7.5) |  | 0.6 (0.4 to 0.9) | 0.5 (0.3 to 0.7) |
| Bangladesh | 9 (6.5 to 12.1) | 6.6 (4.5 to 9.1) |  | 0.8 (0.5 to 1.4) | 0.6 (0.3 to 1.1) |
| Barbados | 3.7 (3.1 to 4.4) | 1.4 (1.1 to 1.9) |  | 0.6 (0.5 to 0.8) | 0.3 (0.2 to 0.4) |
| Belarus | 10.6 (9 to 12.4) | 8.1 (6.4 to 10) |  | 0.8 (0.6 to 1) | 0.4 (0.3 to 0.5) |
| Belgium | 3.7 (3.2 to 4.2) | 0.7 (0.6 to 0.9) |  | 0.4 (0.4 to 0.5) | 0 (0 to 0.1) |
| Belize | 4.4 (3.7 to 5.2) | 2.3 (1.9 to 2.8) |  | 0.3 (0.3 to 0.4) | 0.3 (0.2 to 0.3) |
| Benin | 2 (1.3 to 2.9) | 2.2 (1.4 to 3.3) |  | 0.5 (0.3 to 0.8) | 0.5 (0.3 to 0.8) |
| Bermuda | 5.1 (4.3 to 6) | 1.3 (1 to 1.7) |  | 0.4 (0.3 to 0.5) | 0.1 (0.1 to 0.1) |
| Bhutan | 7.8 (4.3 to 11.9) | 6.1 (3.6 to 9.4) |  | 0.3 (0.2 to 0.6) | 0.2 (0.1 to 0.4) |
| Bolivia | 8.4 (5.7 to 12.3) | 3.7 (2.3 to 5.6) |  | 0.9 (0.5 to 1.5) | 0.3 (0.2 to 0.5) |
| Bosnia and Herzegovina | 6.6 (5.2 to 8.1) | 2.7 (1.8 to 3.8) |  | 0.7 (0.5 to 1.2) | 0.4 (0.3 to 0.6) |
| Botswana | 2.9 (1.4 to 4.8) | 2.2 (1.1 to 3.5) |  | 0.4 (0.2 to 0.8) | 0.3 (0.1 to 0.4) |
| Brazil | 6.9 (6.5 to 7.3) | 4.1 (3.9 to 4.4) |  | 0.9 (0.8 to 0.9) | 0.3 (0.3 to 0.3) |
| Brunei | 11.5 (8.6 to 14.8) | 6.7 (5 to 8.5) |  | 0.7 (0.5 to 1.1) | 0.2 (0.2 to 0.3) |
| Bulgaria | 12.4 (11.1 to 13.7) | 8.7 (7.2 to 10.4) |  | 1.1 (1 to 1.3) | 0.9 (0.7 to 1.1) |
| Burkina Faso | 1.8 (1.2 to 2.7) | 2 (1.2 to 3.1) |  | 0.3 (0.2 to 0.6) | 0.4 (0.2 to 0.6) |
| Burundi | 5.7 (3.6 to 8.7) | 4.9 (3.2 to 6.9) |  | 0.9 (0.5 to 1.5) | 0.4 (0.2 to 0.7) |
| Cabo Verde | 4.3 (2.9 to 6) | 3 (1.9 to 4.5) |  | 1.2 (0.7 to 1.9) | 0.6 (0.4 to 1.1) |
| Cambodia | 7.8 (5.6 to 10.7) | 5.6 (3.5 to 8.6) |  | 0.7 (0.4 to 1.1) | 0.4 (0.2 to 0.6) |
| Cameroon | 2.2 (1.4 to 3.2) | 3.3 (2 to 5.1) |  | 0.6 (0.3 to 0.9) | 0.7 (0.4 to 1.2) |
| Canada | 2.7 (2.4 to 3.1) | 1.3 (1.1 to 1.6) |  | 0.2 (0.1 to 0.2) | 0.1 (0.1 to 0.1) |
| Central African Republic | 4.6 (2.7 to 7.5) | 4.9 (2.7 to 8) |  | 0.5 (0.3 to 0.9) | 0.5 (0.2 to 0.8) |
| Chad | 2.3 (1.4 to 3.5) | 3.1 (1.9 to 4.6) |  | 0.6 (0.3 to 1.2) | 0.7 (0.4 to 1.3) |
| Chile | 2.4 (2.1 to 2.8) | 1.8 (1.5 to 2) |  | 0.4 (0.3 to 0.4) | 0.1 (0.1 to 0.2) |
| China | 5.6 (4.9 to 6.4) | 4.9 (4 to 5.9) |  | 0.7 (0.6 to 0.9) | 0.6 (0.5 to 0.7) |
| Colombia | 6 (5.2 to 6.8) | 2.8 (2.2 to 3.3) |  | 0.6 (0.5 to 0.8) | 0.2 (0.2 to 0.3) |
| Comoros | 4 (1.8 to 6.5) | 4 (2.6 to 5.8) |  | 0.6 (0.3 to 1.2) | 0.4 (0.2 to 0.6) |
| Cook Islands | 10.6 (7 to 15.5) | 7.7 (5 to 11.4) |  | 0.7 (0.4 to 1.1) | 0.4 (0.2 to 0.7) |
| Costa Rica | 4.1 (3.5 to 4.6) | 3.3 (2.8 to 3.9) |  | 0.3 (0.3 to 0.4) | 0.2 (0.1 to 0.2) |
| Croatia | 6.4 (5.6 to 7.1) | 1.6 (1.4 to 1.9) |  | 0.5 (0.4 to 0.6) | 0.1 (0.1 to 0.1) |
| Cuba | 6.8 (6 to 7.7) | 2.8 (2.3 to 3.3) |  | 0.6 (0.4 to 0.7) | 0.3 (0.2 to 0.3) |
| Cyprus | 3 (2.3 to 4) | 1.3 (0.9 to 1.7) |  | 0.2 (0.1 to 0.3) | 0 (0 to 0.1) |
| Czechia | 6.8 (6.1 to 7.6) | 1.8 (1.5 to 2.1) |  | 0.6 (0.5 to 0.7) | 0.1 (0.1 to 0.2) |
| Democratic Republic of the Congo | 3.3 (2 to 5.2) | 2.9 (1.8 to 4.5) |  | 0.4 (0.2 to 0.7) | 0.3 (0.1 to 0.5) |
| Denmark | 3.7 (3.3 to 4.2) | 0.8 (0.7 to 1) |  | 0.3 (0.3 to 0.4) | 0 (0 to 0) |
| Djibouti | 3.1 (1.7 to 4.9) | 4.2 (2.3 to 6.6) |  | 0.4 (0.2 to 0.6) | 0.3 (0.2 to 0.6) |
| Dominica | 2.5 (1.8 to 3.4) | 2 (1.3 to 2.9) |  | 0.3 (0.2 to 0.5) | 0.3 (0.2 to 0.5) |
| Dominican Republic | 9.8 (7.7 to 12.2) | 9.7 (7 to 13.2) |  | 0.7 (0.5 to 1) | 0.7 (0.4 to 1) |
| East Timor | 8 (5.2 to 11.6) | 9.2 (5.7 to 13.8) |  | 0.6 (0.3 to 0.9) | 0.7 (0.3 to 1.2) |
| Ecuador | 6.7 (5.8 to 7.7) | 4.9 (3.8 to 6.2) |  | 1 (0.8 to 1.3) | 0.3 (0.2 to 0.4) |
| Egypt | 30.8 (25.4 to 37) | 22 (17.5 to 27.1) |  | 2.6 (1.3 to 4.6) | 2 (1.2 to 3.2) |
| El Salvador | 9.3 (7.4 to 11.7) | 5.8 (4.1 to 8) |  | 0.7 (0.5 to 1) | 0.3 (0.2 to 0.5) |
| Equatorial Guinea | 4.4 (2.6 to 6.9) | 3.8 (2 to 6.3) |  | 0.5 (0.3 to 0.9) | 0.4 (0.2 to 0.7) |
| Eritrea | 4.4 (2.7 to 6.7) | 5.7 (3.4 to 8.7) |  | 0.5 (0.3 to 0.9) | 0.4 (0.2 to 0.7) |
| Estonia | 9.1 (7.5 to 10.9) | 1.3 (1 to 1.5) |  | 0.6 (0.5 to 0.8) | 0.1 (0.1 to 0.1) |
| eSwatini | 2.1 (1.1 to 3.3) | 4.1 (2.2 to 6.7) |  | 0.3 (0.2 to 0.5) | 0.4 (0.2 to 0.8) |
| Ethiopia | 5.4 (4.1 to 7.6) | 3.1 (2.3 to 4) |  | 0.3 (0.2 to 0.6) | 0.2 (0.1 to 0.3) |
| Federated States of Micronesia | 22.5 (14.7 to 31.9) | 25.1 (16.7 to 35) |  | 1.3 (0.7 to 2.2) | 1.2 (0.7 to 2.1) |
| Fiji | 21.8 (16.9 to 27.7) | 17.8 (12.9 to 23.4) |  | 0.8 (0.5 to 1.1) | 0.6 (0.4 to 0.9) |
| Finland | 5.5 (4.8 to 6.3) | 1.4 (1.2 to 1.6) |  | 0.6 (0.5 to 0.8) | 0.1 (0.1 to 0.1) |
| France | 2.2 (1.9 to 2.5) | 0.8 (0.7 to 1) |  | 0.2 (0.2 to 0.3) | 0.1 (0 to 0.1) |
| Gabon | 3.5 (2.2 to 5.3) | 3.1 (1.7 to 5) |  | 0.4 (0.2 to 0.6) | 0.3 (0.2 to 0.5) |
| Gambia | 3.2 (2 to 4.9) | 4.4 (2.8 to 6.8) |  | 1 (0.5 to 1.6) | 1.1 (0.6 to 1.9) |
| Georgia | 15.1 (14.2 to 16.2) | 5.7 (5.1 to 6.3) |  | 0.7 (0.5 to 1) | 0.6 (0.5 to 0.8) |
| Germany | 4.8 (4.2 to 5.4) | 1 (0.9 to 1.2) |  | 0.6 (0.5 to 0.8) | 0.1 (0.1 to 0.1) |
| Ghana | 5.5 (3.7 to 7.5) | 3.2 (2 to 4.8) |  | 1.6 (0.9 to 2.6) | 1.4 (0.8 to 2.2) |
| Greece | 5.3 (4.7 to 5.9) | 3 (2.6 to 3.4) |  | 0.6 (0.5 to 0.6) | 0.1 (0.1 to 0.2) |
| Greenland | 5.6 (3.8 to 8) | 1.7 (1.1 to 2.4) |  | 0.5 (0.3 to 0.7) | 0.1 (0.1 to 0.2) |
| Grenada | 8 (6.7 to 9.4) | 2.9 (2.3 to 3.6) |  | 1.7 (1.3 to 2) | 0.4 (0.3 to 0.5) |
| Guam | 8.5 (6.2 to 10.8) | 14.2 (11.6 to 17.1) |  | 0.5 (0.3 to 0.7) | 0.5 (0.3 to 0.7) |
| Guatemala | 11.5 (10.2 to 12.9) | 5.8 (4.9 to 6.9) |  | 0.7 (0.5 to 0.8) | 0.3 (0.2 to 0.4) |
| Guinea | 2.5 (1.7 to 3.7) | 3.6 (2.3 to 5.2) |  | 0.7 (0.4 to 1.2) | 0.9 (0.5 to 1.5) |
| Guinea-Bissau | 5.5 (3.6 to 8.3) | 6.1 (3.9 to 9.3) |  | 1.5 (0.9 to 2.4) | 1.5 (0.8 to 2.5) |
| Guyana | 9.6 (7.8 to 11.6) | 5.3 (3.8 to 7.1) |  | 1.3 (1 to 1.7) | 1 (0.7 to 1.4) |
| Haiti | 10.8 (7.4 to 15.2) | 8.1 (5.3 to 11.8) |  | 1.8 (1 to 3) | 1.2 (0.6 to 2) |
| Honduras | 5.3 (3.8 to 7.2) | 2.9 (1.4 to 4.8) |  | 1.3 (0.8 to 2) | 0.5 (0.2 to 0.8) |
| Hungary | 12.6 (11.2 to 14.1) | 2.6 (2.2 to 3.1) |  | 1.2 (1.1 to 1.4) | 0.2 (0.2 to 0.3) |
| Iceland | 4.5 (3.9 to 5.1) | 1.6 (1.3 to 1.9) |  | 0.3 (0.2 to 0.3) | 0 (0 to 0) |
| India | 12.7 (11.1 to 14.4) | 10.4 (9.4 to 11.5) |  | 0.4 (0.2 to 0.5) | 0.2 (0.2 to 0.4) |
| Indonesia | 10.9 (9.2 to 12.8) | 10.7 (8.5 to 14) |  | 1.1 (0.8 to 1.5) | 0.9 (0.6 to 1.4) |
| Iran | 10.9 (9.7 to 12.2) | 6.2 (5.7 to 6.8) |  | 2 (1.7 to 2.4) | 1.1 (1 to 1.3) |
| Iraq | 9.4 (6.9 to 12.4) | 5.3 (3.7 to 7.9) |  | 2.5 (1.7 to 3.6) | 1.4 (0.9 to 2.2) |
| Ireland | 4.1 (3.6 to 4.6) | 1.1 (0.9 to 1.2) |  | 0.3 (0.2 to 0.3) | 0 (0 to 0) |
| Israel | 3.6 (3.2 to 4.1) | 0.6 (0.5 to 0.7) |  | 0.2 (0.2 to 0.3) | 0 (0 to 0) |
| Italy | 3 (2.9 to 3.1) | 1 (1 to 1.1) |  | 0.3 (0.3 to 0.4) | 0.1 (0 to 0.1) |
| Ivory Coast | 3.5 (2.2 to 5.2) | 4.1 (2.5 to 6.2) |  | 1 (0.6 to 1.5) | 1 (0.5 to 1.7) |
| Jamaica | 2 (1.7 to 2.4) | 1.4 (1 to 1.9) |  | 0.6 (0.5 to 0.8) | 0.4 (0.3 to 0.6) |
| Japan | 2.2 (2.1 to 2.2) | 1.1 (1.1 to 1.2) |  | 0.3 (0.3 to 0.3) | 0.1 (0.1 to 0.1) |
| Jordan | 10.4 (8.2 to 13) | 4.4 (3.3 to 5.8) |  | 2 (1.4 to 2.7) | 0.8 (0.6 to 1.1) |
| Kazakhstan | 13 (10.9 to 15.2) | 3.3 (2.5 to 4.3) |  | 1.6 (1.4 to 1.9) | 0.7 (0.5 to 0.8) |
| Kenya | 1.6 (1.1 to 2) | 2.5 (1.8 to 3.3) |  | 0.2 (0.2 to 0.3) | 0.2 (0.2 to 0.3) |
| Kiribati | 17 (11.9 to 23.3) | 21.8 (14.4 to 29.8) |  | 1.2 (0.7 to 1.8) | 1.5 (0.9 to 2.4) |
| Kuwait | 10.5 (9.3 to 11.9) | 7.5 (6.2 to 9.2) |  | 0.7 (0.5 to 0.8) | 0.5 (0.4 to 0.7) |
| Kyrgyzstan | 12.4 (10.6 to 14.3) | 6.6 (5.3 to 8) |  | 1.7 (1.3 to 2) | 1.1 (0.9 to 1.4) |
| Laos | 20.3 (13.7 to 28.6) | 13.2 (8.8 to 19.1) |  | 1.6 (1 to 2.4) | 0.9 (0.6 to 1.5) |
| Latvia | 10.6 (9 to 12.3) | 3.2 (2.5 to 4.1) |  | 0.8 (0.7 to 1) | 0.2 (0.2 to 0.3) |
| Lebanon | 11.9 (8.6 to 16.3) | 4 (3 to 5.2) |  | 1.2 (0.8 to 2) | 0.4 (0.3 to 0.5) |
| Lesotho | 0.9 (0.5 to 1.4) | 2.8 (1.6 to 4.6) |  | 0.2 (0.1 to 0.3) | 0.5 (0.2 to 0.7) |
| Liberia | 2.9 (1.9 to 4.3) | 3.8 (2.5 to 5.7) |  | 0.8 (0.5 to 1.3) | 0.9 (0.5 to 1.5) |
| Libya | 13.2 (9.6 to 17.8) | 13.8 (9.6 to 19) |  | 1.4 (0.8 to 2.2) | 2 (1.1 to 3.1) |
| Lithuania | 11.2 (9.7 to 12.8) | 3.2 (2.7 to 3.8) |  | 0.7 (0.5 to 0.8) | 0.2 (0.1 to 0.2) |
| Luxembourg | 3.8 (3.2 to 4.4) | 0.5 (0.4 to 0.7) |  | 0.6 (0.5 to 0.7) | 0 (0 to 0) |
| Madagascar | 5.9 (4.1 to 8.1) | 6.8 (4.1 to 10.1) |  | 1.1 (0.7 to 1.7) | 0.9 (0.5 to 1.4) |
| Malawi | 4 (2.7 to 5.6) | 5.2 (3.4 to 7.3) |  | 0.5 (0.3 to 0.7) | 0.5 (0.3 to 0.9) |
| Malaysia | 7.3 (5.5 to 9.3) | 7.1 (5.4 to 8.8) |  | 0.6 (0.4 to 0.9) | 0.4 (0.3 to 0.6) |
| Maldives | 14.1 (10.3 to 19.7) | 5.1 (3.6 to 6.8) |  | 1.3 (0.8 to 2) | 0.4 (0.2 to 0.6) |
| Mali | 2.7 (1.7 to 4) | 2.7 (1.8 to 4.2) |  | 0.7 (0.4 to 1.3) | 0.6 (0.3 to 1.2) |
| Malta | 4.1 (3.6 to 4.6) | 1.4 (1.2 to 1.6) |  | 0.4 (0.3 to 0.5) | 0.1 (0 to 0.1) |
| Marshall Islands | 17.9 (12.6 to 25) | 26.1 (18.2 to 36.1) |  | 0.9 (0.5 to 1.5) | 1.2 (0.7 to 1.9) |
| Mauritania | 3.6 (2.2 to 5.4) | 2.5 (1.4 to 3.9) |  | 1.1 (0.6 to 1.9) | 0.6 (0.3 to 1.2) |
| Mauritius | 13.9 (12.3 to 15.6) | 10.5 (9 to 11.8) |  | 1.6 (1.3 to 1.9) | 0.9 (0.8 to 1.1) |
| Mexico | 4.4 (4.2 to 4.5) | 6.7 (6.1 to 7.4) |  | 0.6 (0.6 to 0.7) | 0.3 (0.3 to 0.3) |
| Moldova | 8.5 (7.5 to 9.4) | 6.7 (5.9 to 7.6) |  | 0.5 (0.4 to 0.6) | 0.2 (0.2 to 0.2) |
| Monaco | 3 (2.1 to 4.3) | 1.8 (1 to 2.9) |  | 0.5 (0.4 to 0.8) | 0.2 (0.1 to 0.4) |
| Mongolia | 8.6 (6.4 to 11.5) | 6 (4.5 to 7.9) |  | 0.2 (0.1 to 0.3) | 0.2 (0.1 to 0.4) |
| Montenegro | 7.4 (5.7 to 9.4) | 3.7 (2.7 to 4.8) |  | 0.2 (0.1 to 0.3) | 0.1 (0.1 to 0.2) |
| Morocco | 17.9 (13.2 to 23.8) | 8.6 (5.7 to 13.9) |  | 2.1 (1.2 to 3.3) | 1.4 (0.8 to 2.5) |
| Mozambique | 1 (0.6 to 1.5) | 2.2 (1.2 to 3.7) |  | 0.4 (0.2 to 0.7) | 0.7 (0.4 to 1.2) |
| Myanmar | 17.9 (11.8 to 26.2) | 8.7 (6.1 to 12.3) |  | 1.6 (1 to 2.4) | 0.9 (0.6 to 1.4) |
| Namibia | 2.3 (1.3 to 3.5) | 2.7 (1.4 to 4.5) |  | 0.3 (0.2 to 0.5) | 0.3 (0.1 to 0.5) |
| Nauru | 39.1 (27 to 54.3) | 46.5 (32.8 to 65.8) |  | 2.5 (1.3 to 4.1) | 2.5 (1.4 to 4.2) |
| Nepal | 10 (6.5 to 14.3) | 8 (5.2 to 11.6) |  | 0.4 (0.2 to 0.7) | 0.2 (0.1 to 0.5) |
| Netherlands | 3.3 (2.9 to 3.7) | 0.6 (0.5 to 0.7) |  | 0.3 (0.2 to 0.3) | 0 (0 to 0.1) |
| New Zealand | 3.9 (3.5 to 4.3) | 1.3 (1.1 to 1.5) |  | 0.3 (0.2 to 0.3) | 0.1 (0.1 to 0.1) |
| Nicaragua | 4.5 (3.6 to 5.8) | 3.8 (2.8 to 5) |  | 0.5 (0.4 to 0.7) | 0.3 (0.2 to 0.4) |
| Niger | 1.5 (0.8 to 2.6) | 1.6 (0.9 to 2.6) |  | 0.4 (0.2 to 0.8) | 0.4 (0.2 to 0.7) |
| Nigeria | 2.8 (2 to 3.8) | 3 (2 to 4.1) |  | 0.4 (0.3 to 0.7) | 0.4 (0.3 to 0.7) |
| Niue | 16.7 (10.7 to 25.6) | 17.4 (11.9 to 26.3) |  | 0.8 (0.5 to 1.4) | 0.8 (0.5 to 1.3) |
| North Korea | 8.3 (5.2 to 12.8) | 10.8 (7 to 16.9) |  | 0.8 (0.5 to 1.4) | 1 (0.6 to 1.6) |
| North Macedonia | 7.3 (5.8 to 9.1) | 2.8 (2 to 3.7) |  | 1 (0.7 to 1.4) | 0.4 (0.2 to 0.6) |
| Northern Mariana Islands | 8.3 (5.1 to 12.1) | 8.8 (6.2 to 12.3) |  | 0.7 (0.4 to 1.1) | 0.4 (0.2 to 0.6) |
| Norway | 3.4 (3.2 to 3.6) | 0.5 (0.4 to 0.5) |  | 0.2 (0.2 to 0.3) | 0 (0 to 0) |
| Oman | 8.8 (6 to 12.4) | 4.7 (3.4 to 6.3) |  | 1.2 (0.7 to 1.7) | 0.7 (0.5 to 1) |
| Pakistan | 10.5 (7.4 to 13.4) | 15.5 (11.7 to 20.1) |  | 0.4 (0.2 to 0.6) | 0.5 (0.3 to 0.8) |
| Palau | 19.3 (12.9 to 27.1) | 25.7 (18.8 to 33.9) |  | 1.4 (0.8 to 2.2) | 1.8 (1.1 to 2.6) |
| Palestine | 10.3 (7.4 to 14.2) | 5.8 (4.5 to 7.4) |  | 1.6 (1 to 2.4) | 0.9 (0.6 to 1.2) |
| Panama | 3 (2.6 to 3.5) | 2.8 (2.2 to 3.5) |  | 0.6 (0.5 to 0.7) | 0.3 (0.2 to 0.3) |
| Papua New Guinea | 10.1 (5.6 to 16.1) | 11.3 (6.9 to 16.6) |  | 0.4 (0.2 to 0.8) | 0.4 (0.2 to 0.6) |
| Paraguay | 4.2 (3.2 to 5.4) | 2.8 (2 to 3.8) |  | 0.4 (0.3 to 0.5) | 0.2 (0.1 to 0.3) |
| Peru | 5.9 (4.4 to 7.7) | 3.5 (2.4 to 5) |  | 0.5 (0.4 to 0.8) | 0.3 (0.2 to 0.5) |
| Philippines | 17.5 (15.9 to 19.4) | 15.3 (13 to 17.8) |  | 0.7 (0.6 to 0.8) | 0.8 (0.6 to 0.9) |
| Poland | 12.2 (11.8 to 12.6) | 1.4 (1.3 to 1.5) |  | 0.7 (0.7 to 0.8) | 0.2 (0.2 to 0.3) |
| Portugal | 3.9 (3.3 to 4.5) | 1.3 (1.1 to 1.6) |  | 1.1 (1 to 1.3) | 0.1 (0.1 to 0.2) |
| Puerto Rico | 5 (4.3 to 5.8) | 2.6 (2.1 to 3.2) |  | 0.3 (0.3 to 0.4) | 0.1 (0.1 to 0.1) |
| Qatar | 8 (5.7 to 10.7) | 2.7 (1.9 to 3.8) |  | 0.4 (0.3 to 0.7) | 0.2 (0.1 to 0.3) |
| Republic of Serbia | 7.4 (5.8 to 9) | 2.4 (1.7 to 3.1) |  | 1 (0.7 to 1.4) | 0.3 (0.2 to 0.4) |
| Republic of the Congo | 5.8 (3.4 to 9.1) | 4.8 (3 to 7.6) |  | 0.6 (0.3 to 1) | 0.5 (0.3 to 0.8) |
| Romania | 8.6 (7.6 to 9.7) | 4.5 (3.8 to 5.2) |  | 0.8 (0.6 to 1) | 0.4 (0.3 to 0.4) |
| Russia | 11.6 (11.2 to 11.9) | 6.6 (6.1 to 7.1) |  | 1.2 (1.1 to 1.2) | 0.8 (0.8 to 0.9) |
| Rwanda | 5.5 (3.4 to 8.5) | 2.7 (1.7 to 4.3) |  | 0.9 (0.5 to 1.5) | 0.2 (0.1 to 0.4) |
| Saint Kitts and Nevis | 8.4 (7.2 to 9.7) | 1.3 (0.8 to 1.9) |  | 1.9 (1.5 to 2.4) | 0.3 (0.2 to 0.5) |
| Saint Lucia | 4.1 (3.5 to 4.8) | 1.3 (1 to 1.7) |  | 1.1 (0.9 to 1.3) | 0.4 (0.3 to 0.5) |
| Saint Vincent and the Grenadines | 7.8 (6.8 to 9) | 3.1 (2.5 to 3.8) |  | 0.9 (0.7 to 1.1) | 0.5 (0.4 to 0.6) |
| Samoa | 11 (7.2 to 16.1) | 15.8 (10.3 to 22.6) |  | 0.6 (0.3 to 0.9) | 0.6 (0.4 to 1) |
| San Marino | 1.4 (1 to 1.9) | 0.5 (0.2 to 0.7) |  | 0.2 (0.2 to 0.3) | 0.1 (0 to 0.1) |
| São Tomé and Principe | 2.8 (1.8 to 4.2) | 3.1 (1.7 to 5.1) |  | 0.9 (0.5 to 1.4) | 1 (0.5 to 1.8) |
| Saudi Arabia | 10.5 (7.2 to 14.6) | 12.3 (8 to 18.1) |  | 1.8 (1.1 to 2.7) | 1.7 (1.1 to 2.7) |
| Senegal | 4 (2.7 to 5.7) | 3.3 (2.1 to 4.9) |  | 1.1 (0.7 to 1.8) | 0.7 (0.4 to 1.3) |
| Seychelles | 11.5 (9 to 14.3) | 5.9 (4.6 to 7.4) |  | 1.1 (0.8 to 1.7) | 0.6 (0.4 to 0.8) |
| Sierra Leone | 3.5 (2.2 to 5.3) | 4.5 (2.8 to 6.8) |  | 1 (0.6 to 1.7) | 1.1 (0.6 to 1.9) |
| Singapore | 6.1 (5.4 to 6.9) | 2.2 (1.9 to 2.5) |  | 0.4 (0.3 to 0.5) | 0 (0 to 0.1) |
| Slovakia | 9 (7.2 to 11) | 2.8 (2.1 to 3.5) |  | 0.6 (0.4 to 0.9) | 0.2 (0.1 to 0.3) |
| Slovenia | 3.3 (2.8 to 3.8) | 0.6 (0.5 to 0.7) |  | 0.3 (0.3 to 0.4) | 0 (0 to 0) |
| Solomon Islands | 18.2 (9.4 to 26.4) | 20.1 (13.7 to 27.8) |  | 0.4 (0.2 to 0.8) | 0.5 (0.3 to 0.8) |
| Somalia | 3.1 (1.9 to 5.1) | 3.7 (2.1 to 6) |  | 0.4 (0.2 to 0.8) | 0.3 (0.1 to 0.6) |
| South Africa | 7 (6 to 8.3) | 3.3 (2.9 to 4) |  | 1.1 (0.9 to 1.3) | 0.5 (0.4 to 0.6) |
| South Korea | 3 (2.1 to 4.1) | 1 (0.7 to 1.3) |  | 0.7 (0.5 to 0.9) | 0.1 (0.1 to 0.2) |
| South Sudan | 3 (1.9 to 4.7) | 4.2 (2.6 to 6.6) |  | 0.4 (0.2 to 0.6) | 0.3 (0.2 to 0.6) |
| Spain | 3.9 (3.4 to 4.5) | 1.3 (1.1 to 1.5) |  | 0.4 (0.4 to 0.5) | 0.1 (0.1 to 0.1) |
| Sri Lanka | 7.9 (6.1 to 10.1) | 5.7 (3.8 to 8) |  | 1.1 (0.8 to 1.5) | 0.7 (0.4 to 1) |
| Sudan | 22.6 (15.4 to 31.9) | 14.7 (8.4 to 21.7) |  | 2.3 (1.2 to 4.1) | 1.9 (0.9 to 3.4) |
| Suriname | 9.2 (6.6 to 11.9) | 4.8 (3.4 to 6.6) |  | 0.9 (0.6 to 1.3) | 0.6 (0.4 to 0.9) |
| Sweden | 2.2 (2 to 2.5) | 0.4 (0.3 to 0.5) |  | 0.3 (0.2 to 0.3) | 0 (0 to 0) |
| Switzerland | 3.5 (3 to 4) | 0.5 (0.5 to 0.6) |  | 0.3 (0.2 to 0.3) | 0 (0 to 0) |
| Syria | 33.1 (25.8 to 40.9) | 17.1 (12.5 to 23.2) |  | 3 (2 to 4.2) | 1.5 (1 to 2.2) |
| Tajikistan | 12 (9.7 to 14.5) | 6.5 (4.7 to 8.4) |  | 1.6 (1.1 to 2.3) | 0.7 (0.5 to 1.1) |
| Thailand | 4.5 (3 to 6.3) | 5.1 (3.5 to 7.3) |  | 0.4 (0.3 to 0.6) | 0.7 (0.4 to 1) |
| The Bahamas | 6.9 (5.9 to 8.1) | 3.4 (2.6 to 4.5) |  | 0.6 (0.5 to 0.7) | 0.4 (0.3 to 0.5) |
| Togo | 3.4 (2.3 to 5) | 3.6 (2.2 to 5.5) |  | 1 (0.6 to 1.5) | 0.9 (0.5 to 1.4) |
| Tokelau | 13.1 (8.3 to 19.9) | 17.4 (12.7 to 24.1) |  | 0.8 (0.4 to 1.3) | 0.9 (0.6 to 1.4) |
| Tonga | 7.7 (5.6 to 10.2) | 9.2 (6.2 to 14) |  | 0.3 (0.2 to 0.5) | 0.3 (0.2 to 0.5) |
| Trinidad and Tobago | 8.4 (7.4 to 9.6) | 5.9 (4.4 to 7.9) |  | 1 (0.8 to 1.2) | 0.6 (0.5 to 0.8) |
| Tunisia | 8.6 (6.2 to 11.5) | 6.1 (4 to 8.7) |  | 1 (0.6 to 1.5) | 0.8 (0.5 to 1.3) |
| Turkey | 9.8 (7.1 to 12.9) | 3.7 (2.7 to 4.9) |  | 1.3 (0.9 to 1.9) | 0.4 (0.3 to 0.6) |
| Turkmenistan | 14.4 (12.5 to 16.3) | 10.4 (7.9 to 13.7) |  | 1.6 (1.1 to 2.1) | 1.9 (1.2 to 2.6) |
| Tuvalu | 19.3 (13.4 to 26.1) | 24.2 (17 to 32.1) |  | 1.2 (0.7 to 1.8) | 1.1 (0.7 to 1.8) |
| Uganda | 2.7 (1.6 to 4.1) | 3.7 (2.3 to 5.6) |  | 0.3 (0.2 to 0.5) | 0.3 (0.1 to 0.4) |
| Ukraine | 7.7 (6.2 to 9.3) | 9.8 (6.9 to 13.4) |  | 0.8 (0.6 to 1) | 0.8 (0.6 to 1.1) |
| United Arab Emirates | 6.9 (4.5 to 9.9) | 3.4 (2.2 to 4.7) |  | 0.9 (0.5 to 1.4) | 0.4 (0.3 to 0.7) |
| United Kingdom | 3.8 (3.7 to 3.9) | 1.3 (1.2 to 1.3) |  | 0.3 (0.3 to 0.3) | 0.1 (0 to 0.1) |
| United Republic of Tanzania | 3.7 (2.5 to 5.3) | 5.5 (3.4 to 8.1) |  | 0.3 (0.2 to 0.5) | 0.4 (0.2 to 0.6) |
| United States of America | 3.8 (3.7 to 3.9) | 2.6 (2.4 to 2.8) |  | 0.2 (0.2 to 0.2) | 0.2 (0.1 to 0.2) |
| United States Virgin Islands | 8.7 (6.2 to 11.7) | 6.4 (3.7 to 9.7) |  | 0.5 (0.3 to 0.7) | 0.3 (0.2 to 0.6) |
| Uruguay | 4.7 (4.1 to 5.4) | 1.9 (1.6 to 2.3) |  | 0.6 (0.5 to 0.7) | 0.2 (0.2 to 0.2) |
| Uzbekistan | 12.8 (11.3 to 14.5) | 11 (9 to 13) |  | 1.4 (1.2 to 1.7) | 0.9 (0.8 to 1.1) |
| Vanuatu | 24 (15.3 to 33.9) | 28.2 (19.5 to 37.7) |  | 1.1 (0.6 to 1.9) | 1.1 (0.7 to 1.8) |
| Venezuela | 8.5 (7.6 to 9.6) | 7.6 (5.7 to 10) |  | 0.5 (0.4 to 0.6) | 0.4 (0.3 to 0.5) |
| Vietnam | 3.2 (2.1 to 4.7) | 2.8 (1.8 to 4.3) |  | 0.6 (0.4 to 1) | 0.5 (0.3 to 0.9) |
| Yemen | 13.4 (7.6 to 21.1) | 9.8 (6 to 15.1) |  | 1.5 (0.7 to 2.6) | 1.5 (0.7 to 2.5) |
| Zambia | 2.9 (2 to 4.3) | 3.9 (2.3 to 6.1) |  | 0.4 (0.2 to 0.7) | 0.4 (0.2 to 0.7) |
| Zimbabwe | 1.3 (0.9 to 1.8) | 3.7 (2.3 to 5.6) |  | 0.2 (0.1 to 0.3) | 0.6 (0.3 to 1) |

| **Supplementary Table 6. Age standardized DALYs rate of ischemic heart disease vs ischemic stroke in youths and young Adults (15-39 years) at country level, 1990-2021, both sexes** | | | | | |
| --- | --- | --- | --- | --- | --- |
|  | **Ischemic heart disease (DALYs rate, 95% UI)** | |  | **Ischemic stroke (DALYs rate, 95% UI)** | |
|  | **Age standardized rate in 1990 (per 100,000)** | **Age standardized rate in 2021 (per 100,000)** |  | **Age standardized rate in 1990 (per 100,000)** | **Age standardized rate in 2021 (per 100,000)** |
| Afghanistan | 1180.1 (753.4 to 1729.4) | 952.1 (631.3 to 1427.3) |  | 185.9 (116.1 to 286.6) | 183.3 (116.5 to 295.1) |
| Albania | 275.8 (215 to 348.5) | 194.5 (144.8 to 259) |  | 48.8 (33.5 to 67.3) | 41.2 (27.5 to 58.7) |
| Algeria | 858.5 (621.3 to 1161.2) | 405.3 (282.8 to 556.6) |  | 139.5 (96.1 to 195.9) | 97.1 (67.5 to 136.9) |
| American Samoa | 666.2 (472.9 to 916.4) | 815.1 (561.2 to 1098.7) |  | 90.8 (66.5 to 119.5) | 82.1 (58.2 to 109.9) |
| Andorra | 119.4 (80.9 to 172.2) | 51.3 (33 to 75.3) |  | 27 (19.1 to 36.4) | 19.4 (12.8 to 27.9) |
| Angola | 195.7 (122.3 to 288.1) | 201.2 (127.7 to 302.3) |  | 56.8 (39.9 to 77.9) | 51 (35.9 to 70) |
| Antigua and Barbuda | 264.4 (225.4 to 310.3) | 60.5 (48.1 to 74.7) |  | 46.7 (38.4 to 56.3) | 23.9 (18.3 to 30.6) |
| Argentina | 358.7 (313.6 to 407.7) | 123.7 (104.5 to 143.3) |  | 45.6 (35.9 to 57.6) | 25 (17.8 to 34.2) |
| Armenia | 554.3 (482.4 to 622.7) | 296 (255.1 to 339.7) |  | 88.6 (67 to 112.4) | 57 (41.9 to 76.1) |
| Australia | 172.6 (149.5 to 197.8) | 65.2 (54.8 to 76.8) |  | 30.5 (22.8 to 40.2) | 21 (14.1 to 29.3) |
| Austria | 192.9 (169.3 to 221.6) | 54.5 (46.4 to 62.8) |  | 47.7 (37.1 to 60.7) | 23.1 (15 to 33.4) |
| Azerbaijan | 895.5 (745.6 to 1051.8) | 425 (328.4 to 532.1) |  | 76.6 (56.2 to 100.2) | 49.4 (33.8 to 67.5) |
| Bahrain | 698.5 (564.4 to 851.2) | 350.7 (266.8 to 446.2) |  | 79.2 (58.2 to 102.2) | 62.3 (45.7 to 81.1) |
| Bangladesh | 523.2 (375.3 to 707.4) | 385.2 (266.6 to 531.4) |  | 67.5 (45.4 to 101.3) | 56.2 (35.7 to 89) |
| Barbados | 217.6 (183 to 256.5) | 87 (64.4 to 115.6) |  | 52.9 (43.3 to 63.4) | 32.8 (25.4 to 41.7) |
| Belarus | 600.2 (510.4 to 700.8) | 453.5 (359.4 to 559.1) |  | 88.9 (67.8 to 112) | 62.4 (45.7 to 82.7) |
| Belgium | 214.3 (187.1 to 242.6) | 45.5 (38.6 to 52.9) |  | 45.5 (36.2 to 57) | 19 (13 to 27.1) |
| Belize | 261.9 (220.5 to 306.2) | 137.9 (113 to 166.1) |  | 33.4 (26.7 to 40.8) | 28.4 (22.5 to 35.6) |
| Benin | 114.8 (76.4 to 169.2) | 131.8 (84.7 to 192.8) |  | 68 (48.4 to 91.3) | 66.1 (47.3 to 90) |
| Bermuda | 298.5 (253.1 to 350.4) | 80.7 (62.9 to 103.6) |  | 37 (28.6 to 46.3) | 21.2 (15.7 to 28.2) |
| Bhutan | 453.9 (255.4 to 692.2) | 359.7 (212.7 to 550.2) |  | 39.6 (26.5 to 55.9) | 32.5 (21.7 to 46.2) |
| Bolivia | 503.6 (344.2 to 734.5) | 222.2 (142.3 to 340.3) |  | 69.7 (44.4 to 104.8) | 32.2 (21.9 to 44.4) |
| Bosnia and Herzegovina | 376.3 (301.2 to 465.8) | 161.9 (110.5 to 220.1) |  | 99.6 (71.7 to 132.5) | 84.3 (60.9 to 110.8) |
| Botswana | 166.4 (81.6 to 276) | 126.5 (64.9 to 200.4) |  | 55.5 (35.7 to 79.7) | 44.1 (29.6 to 61.7) |
| Brazil | 395.2 (374 to 417.8) | 244.1 (229.6 to 260.2) |  | 64.1 (58.6 to 70.5) | 29.5 (25.9 to 33.7) |
| Brunei | 682.1 (512.6 to 884.6) | 382.2 (286.6 to 488) |  | 74 (54 to 100.4) | 36.8 (26.1 to 49.4) |
| Bulgaria | 707.2 (632.3 to 787.2) | 503.2 (414.3 to 599.3) |  | 105.8 (87.6 to 126.3) | 92.4 (73.8 to 114.1) |
| Burkina Faso | 106.1 (68.5 to 159.3) | 117.8 (72.6 to 180.4) |  | 53.3 (37 to 74.6) | 51.3 (35.5 to 71) |
| Burundi | 340.6 (217.2 to 515.1) | 292.3 (193.5 to 415.3) |  | 86.2 (57.7 to 125.3) | 51.9 (35.8 to 73.7) |
| Cabo Verde | 247.9 (170.9 to 345.1) | 176.4 (112.4 to 264) |  | 111.5 (80 to 153.5) | 76.7 (53.7 to 106.9) |
| Cambodia | 457.8 (326.6 to 625.9) | 325.8 (209 to 500.8) |  | 71.1 (50.7 to 97.7) | 52.7 (36.9 to 73.6) |
| Cameroon | 128.1 (80 to 187.5) | 194.4 (116.7 to 301.2) |  | 69.1 (48.1 to 97.4) | 78.7 (54.1 to 113) |
| Canada | 160.4 (139.9 to 182.7) | 78.9 (67 to 92.1) |  | 42.3 (30.1 to 58.1) | 34.1 (23 to 47.8) |
| Central African Republic | 266.5 (158.2 to 429.5) | 282.7 (158.2 to 461) |  | 59 (40.3 to 83.3) | 53.5 (34.9 to 76.7) |
| Chad | 133.6 (85.2 to 201.2) | 183.6 (114 to 270.4) |  | 77.2 (53.2 to 112.6) | 82.1 (56.7 to 119.1) |
| Chile | 143.5 (122.6 to 166.9) | 104.8 (87.9 to 122) |  | 43.4 (34.6 to 55) | 28.8 (20.8 to 39.4) |
| China | 330.2 (286 to 377.8) | 288.2 (238 to 344.8) |  | 80 (64.2 to 98.2) | 70.4 (55.7 to 86.3) |
| Colombia | 352.6 (309 to 401.6) | 165.9 (135.1 to 199.3) |  | 54.1 (45.7 to 63.5) | 24 (19 to 29.8) |
| Comoros | 241 (105.1 to 384.1) | 240.7 (157.3 to 342.3) |  | 71.5 (45.4 to 106.1) | 51 (35.6 to 70.2) |
| Cook Islands | 598.7 (398.4 to 877.8) | 438 (286.6 to 645.2) |  | 87.3 (61.6 to 119.8) | 73.2 (49.9 to 100.2) |
| Costa Rica | 239.3 (207.5 to 272.3) | 197.1 (166.5 to 229.6) |  | 34.9 (28.1 to 43.9) | 23.4 (17.9 to 30) |
| Croatia | 365.3 (324.4 to 409.2) | 98 (81.9 to 115.2) |  | 60.4 (46.8 to 77.1) | 32 (21.3 to 44.9) |
| Cuba | 393.4 (348.7 to 442.6) | 162.2 (132.7 to 193.3) |  | 48.2 (39.7 to 58) | 28.7 (22.5 to 35.8) |
| Cyprus | 175.4 (133.4 to 228.4) | 76 (55.8 to 101.5) |  | 25.1 (17.9 to 34) | 16.3 (11 to 23) |
| Czechia | 389.5 (348.7 to 433.4) | 109.1 (93.2 to 125.8) |  | 73.3 (57.1 to 93) | 37.3 (24.9 to 52.5) |
| Democratic Republic of the Congo | 191.3 (115.5 to 299.7) | 171.2 (105.9 to 263.3) |  | 51.2 (35.6 to 72.4) | 40.2 (26.8 to 56.6) |
| Denmark | 214.5 (188.5 to 243.3) | 51 (43.4 to 59.3) |  | 39.5 (31.1 to 49.8) | 18.1 (12.1 to 25.6) |
| Djibouti | 182.4 (104 to 286.4) | 249.3 (140.3 to 388.1) |  | 52.2 (35.6 to 73.2) | 50.8 (34.8 to 71.4) |
| Dominica | 147.8 (108.6 to 197.4) | 117.6 (79 to 170) |  | 34.5 (25.8 to 45.1) | 33.5 (24.6 to 44.7) |
| Dominican Republic | 573.4 (449.7 to 715.3) | 571.7 (409.2 to 778.1) |  | 56.1 (41.5 to 74.5) | 55.1 (39.5 to 75.8) |
| East Timor | 467.2 (305.8 to 677.8) | 547 (340.6 to 819) |  | 66.3 (47.1 to 91) | 72.3 (46.8 to 104.2) |
| Ecuador | 401.1 (348.1 to 456.9) | 295.7 (232.1 to 371.2) |  | 78.3 (63.6 to 93.1) | 31 (24.1 to 39.1) |
| Egypt | 1838.9 (1511.6 to 2209.5) | 1317.6 (1045.6 to 1623) |  | 194.4 (113.3 to 312.1) | 164.2 (115.3 to 232.9) |
| El Salvador | 552.9 (442.9 to 696.4) | 342.5 (246.1 to 466.9) |  | 55.8 (41.6 to 73.1) | 31.1 (22.9 to 41.6) |
| Equatorial Guinea | 254.5 (151.7 to 398.5) | 224.6 (120.1 to 369.3) |  | 60.5 (40.6 to 86.2) | 49.6 (33.7 to 72.1) |
| Eritrea | 256.1 (158.3 to 391.5) | 333.5 (199.6 to 509.9) |  | 57.6 (38.1 to 85.1) | 51 (34.7 to 72) |
| Estonia | 512.6 (426.5 to 613.1) | 74.2 (59.6 to 90.5) |  | 73.1 (55.9 to 92.4) | 37.2 (24.5 to 53.4) |
| eSwatini | 123.1 (67 to 191.3) | 236.2 (131 to 384.8) |  | 43.1 (29.1 to 60) | 50.2 (33.1 to 73.3) |
| Ethiopia | 324.9 (244.1 to 456.3) | 188.4 (140.2 to 241.4) |  | 45.3 (31.5 to 65.6) | 31.6 (22.9 to 41.7) |
| Federated States of Micronesia | 1261.5 (829.5 to 1790.6) | 1418.7 (943.6 to 1978) |  | 132.8 (90.8 to 188.4) | 124.9 (87.3 to 172.5) |
| Fiji | 1237.3 (953.7 to 1576.4) | 1010.7 (734.1 to 1335.3) |  | 106.8 (78.5 to 140.4) | 93.4 (67.1 to 124.5) |
| Finland | 318.7 (275.9 to 363.1) | 82.6 (70.1 to 96.7) |  | 59 (47.1 to 72.1) | 25 (17 to 35.4) |
| France | 126 (108.1 to 144.8) | 48.9 (41.4 to 57.7) |  | 29.4 (23 to 37.4) | 15.9 (11.4 to 21.6) |
| Gabon | 199.9 (125.4 to 301.6) | 178.6 (100.1 to 286.3) |  | 51.7 (36.7 to 69.8) | 47.3 (32.2 to 65.3) |
| Gambia | 186.5 (116 to 284.1) | 259.2 (165.5 to 398.8) |  | 103.4 (71.4 to 147.6) | 104.9 (70.1 to 152.1) |
| Georgia | 860.7 (805.7 to 923.8) | 326.3 (291.8 to 359) |  | 99.2 (73 to 126.4) | 86.6 (66 to 109.7) |
| Germany | 276.5 (242.9 to 311.1) | 59.9 (51.4 to 70.3) |  | 64.3 (51 to 79.7) | 27.3 (18.5 to 39.2) |
| Ghana | 315.7 (216.2 to 432.2) | 186.4 (120.9 to 279.2) |  | 150.6 (106.1 to 211) | 138.9 (98.6 to 189.1) |
| Greece | 306.7 (272.3 to 341.9) | 173.3 (151.5 to 196.9) |  | 54.9 (44.6 to 66.6) | 25.3 (18.2 to 34.5) |
| Greenland | 316.3 (217.8 to 457.1) | 96.8 (62.7 to 135.7) |  | 66.1 (48 to 88.5) | 35.2 (23.6 to 49.6) |
| Grenada | 460.4 (388.4 to 542.9) | 165.1 (131.8 to 206.2) |  | 114.9 (93.5 to 138.8) | 39.9 (32 to 49.6) |
| Guam | 486.5 (355.8 to 620.5) | 807.6 (659.4 to 976.1) |  | 75.3 (54.1 to 99.6) | 83.7 (60.5 to 109.9) |
| Guatemala | 685.2 (606.4 to 768.5) | 349 (291.2 to 414.4) |  | 52.3 (41.3 to 64) | 29.5 (23.9 to 36.1) |
| Guinea | 147 (99.3 to 214.1) | 211.6 (137.7 to 307.7) |  | 81.3 (56.8 to 112.8) | 93.4 (65.5 to 131.4) |
| Guinea-Bissau | 322 (207.9 to 480.9) | 357.2 (227 to 537.6) |  | 135 (94.5 to 193.1) | 128.8 (86.9 to 191.9) |
| Guyana | 556.8 (451 to 669.9) | 305.4 (222.5 to 407.8) |  | 98.1 (79.3 to 120.3) | 74.9 (56.7 to 99.2) |
| Haiti | 626 (428.6 to 882.1) | 472.4 (308.7 to 683.8) |  | 123.8 (75.6 to 193.5) | 84.9 (51.1 to 133.6) |
| Honduras | 308.8 (220.2 to 417.4) | 170.1 (86.7 to 276.4) |  | 90.8 (61 to 132.9) | 40.3 (24.9 to 62.6) |
| Hungary | 708.6 (630.5 to 791.6) | 150.8 (127 to 177.2) |  | 115.3 (96 to 138.7) | 47.3 (33.6 to 65) |
| Iceland | 263.4 (228.6 to 302) | 95.4 (80.1 to 112) |  | 38.3 (29.2 to 48.9) | 19.9 (13.1 to 28.5) |
| India | 739.7 (644.4 to 842.9) | 603.8 (542.4 to 666) |  | 42.4 (31.9 to 53.7) | 34.4 (25.6 to 45.4) |
| Indonesia | 634.9 (537.1 to 745.6) | 624.6 (500.7 to 818) |  | 121.5 (96.1 to 150.9) | 100.6 (74.7 to 129.5) |
| Iran | 648.1 (574.3 to 718.7) | 375.2 (345.6 to 406.9) |  | 173.7 (149.3 to 203) | 108.3 (93.2 to 124) |
| Iraq | 546.9 (407.1 to 723.7) | 315.9 (221.1 to 463.3) |  | 197.3 (145.4 to 263.6) | 130.5 (94 to 181.4) |
| Ireland | 237.4 (210.8 to 267.7) | 62.6 (52.7 to 73.8) |  | 36.2 (28 to 46.5) | 16.1 (10.6 to 23.3) |
| Israel | 213.4 (189 to 240) | 38.4 (32.9 to 44.6) |  | 32.5 (24.6 to 42.3) | 17.4 (11.4 to 25.1) |
| Italy | 174.6 (166.8 to 182.1) | 64 (59.8 to 68.4) |  | 41.6 (34.2 to 50.3) | 19.7 (14.3 to 26) |
| Ivory Coast | 206.8 (131.6 to 301) | 239 (149.6 to 360.8) |  | 105.5 (76.1 to 144.7) | 101.4 (69.5 to 146.9) |
| Jamaica | 120.5 (100.6 to 143.3) | 83.3 (60.3 to 114) |  | 51.2 (40.2 to 63.1) | 36.9 (28.1 to 48.3) |
| Japan | 128.5 (123.5 to 133.6) | 66.6 (63.5 to 70.1) |  | 40.7 (32.3 to 50.4) | 28.5 (20.7 to 37.5) |
| Jordan | 612.6 (483.6 to 762) | 264.1 (199.1 to 343.6) |  | 170.4 (127.3 to 220) | 93.4 (70.6 to 119.7) |
| Kazakhstan | 736 (622 to 860.4) | 189.7 (143.8 to 245) |  | 166.4 (138.2 to 196.2) | 93.2 (71.2 to 118.5) |
| Kenya | 95.6 (71 to 121.8) | 150.8 (112.2 to 200.4) |  | 46.4 (34.6 to 59.9) | 41.7 (31.3 to 53.1) |
| Kiribati | 961.4 (670.8 to 1319.5) | 1231 (812.2 to 1688.9) |  | 145 (105.8 to 190.3) | 156 (109.8 to 213.8) |
| Kuwait | 633.8 (562.6 to 711.8) | 450.2 (370.7 to 547) |  | 87.2 (69 to 109.3) | 74.9 (57 to 96.8) |
| Kyrgyzstan | 715.4 (611.6 to 826.8) | 374 (303.2 to 451.2) |  | 149 (121 to 177.3) | 98.8 (78.5 to 122.7) |
| Laos | 1186.2 (800.1 to 1674.6) | 780.1 (515.6 to 1127.8) |  | 130.3 (91.7 to 184.4) | 93 (64.7 to 130.8) |
| Latvia | 599.4 (510.2 to 692) | 182.8 (143 to 230.7) |  | 85.2 (67.3 to 106.2) | 46.6 (33.2 to 62.4) |
| Lebanon | 686.3 (494.3 to 938.2) | 244.5 (186 to 315.6) |  | 113.4 (82.7 to 163.4) | 69.9 (51.5 to 91.1) |
| Lesotho | 51.2 (28.9 to 81) | 162.7 (95.6 to 266.2) |  | 32.5 (20.5 to 46.6) | 48 (32.8 to 67.6) |
| Liberia | 171.2 (114.1 to 250.9) | 224.7 (145 to 335.7) |  | 87.8 (61.5 to 118.9) | 87 (59.2 to 126.5) |
| Libya | 788.2 (569.3 to 1055.4) | 818.5 (575.2 to 1126.8) |  | 132.4 (92.7 to 182.3) | 169.2 (114.9 to 240.2) |
| Lithuania | 629.1 (544.2 to 719.4) | 181.5 (152 to 215.2) |  | 83.6 (63 to 108.9) | 50.3 (34.3 to 71.1) |
| Luxembourg | 218.6 (186.8 to 256.2) | 34 (28.3 to 40.6) |  | 54.6 (44.4 to 66.5) | 17.4 (11.6 to 24.9) |
| Madagascar | 346.3 (242.8 to 476.4) | 399.3 (244.6 to 591.6) |  | 101.9 (73.2 to 140.9) | 87.7 (60.7 to 121.9) |
| Malawi | 241.2 (165.1 to 334) | 306.2 (203.8 to 433.1) |  | 58.7 (40.7 to 81.1) | 61.2 (42.2 to 85.5) |
| Malaysia | 427.2 (323.4 to 545.5) | 408.9 (314.3 to 510.7) |  | 78.7 (58.3 to 103.5) | 67.2 (48.9 to 88.9) |
| Maldives | 817.7 (595.4 to 1135.8) | 299.6 (212.5 to 402.4) |  | 120.2 (87.4 to 163.6) | 52.1 (36.4 to 69.6) |
| Mali | 156.2 (99.3 to 234.9) | 163.3 (106.8 to 247) |  | 79.5 (53.2 to 118.3) | 72.9 (49.6 to 106) |
| Malta | 240.4 (211.3 to 268.8) | 83.5 (71.8 to 96.7) |  | 43.9 (35 to 54.2) | 18.9 (13 to 26.2) |
| Marshall Islands | 1029.4 (722.8 to 1442.8) | 1471.6 (1025.7 to 2034.9) |  | 107.2 (75.7 to 148.8) | 118.5 (81.3 to 168.9) |
| Mauritania | 210.4 (133 to 311.1) | 146.3 (83 to 231.1) |  | 112.4 (76.5 to 163.8) | 75.4 (50 to 113.3) |
| Mauritius | 793.2 (700.7 to 886.2) | 607.4 (525.5 to 686.1) |  | 136.2 (112.6 to 163) | 87.2 (70.4 to 106.1) |
| Mexico | 259 (249.6 to 268.3) | 398.9 (362.2 to 439.7) |  | 58.2 (51.4 to 65.8) | 33.5 (28.1 to 39.7) |
| Moldova | 486.7 (433.3 to 542.3) | 385.4 (336.8 to 437.1) |  | 63.8 (47.8 to 82.8) | 46.7 (33 to 64.5) |
| Monaco | 172.9 (118.9 to 244) | 102.2 (57.7 to 163.9) |  | 52.5 (38.8 to 69.5) | 33.5 (23 to 46.8) |
| Mongolia | 491.7 (367.5 to 654.9) | 341.1 (255.9 to 448.4) |  | 67.9 (47.1 to 92.2) | 64.4 (45.1 to 87.1) |
| Montenegro | 428 (329.6 to 539.2) | 212.6 (156.2 to 278.6) |  | 42.5 (29.3 to 59.3) | 37.3 (24.8 to 52.2) |
| Morocco | 1046.4 (772.2 to 1394.2) | 508.1 (340.2 to 814.1) |  | 166 (108.6 to 245.9) | 120.9 (80.5 to 187) |
| Mozambique | 61.1 (39.6 to 89.4) | 129.9 (74.4 to 217.4) |  | 54.7 (38.6 to 74.9) | 72.1 (47.6 to 104.2) |
| Myanmar | 1050.8 (692.5 to 1544) | 511.5 (359.1 to 727.6) |  | 132.6 (95 to 186.1) | 90.3 (64.3 to 124.2) |
| Namibia | 133.8 (75.4 to 202.7) | 153.2 (80.5 to 255.7) |  | 48.5 (32.6 to 66.8) | 41.4 (26.5 to 59.4) |
| Nauru | 2205.9 (1521.7 to 3072.7) | 2638.7 (1859.1 to 3745.4) |  | 226.9 (151 to 325.4) | 233.4 (164.1 to 336.9) |
| Nepal | 583.5 (380 to 839.4) | 470.3 (309.1 to 681.2) |  | 44.1 (28.8 to 63.6) | 31.8 (20.7 to 47.3) |
| Netherlands | 186.6 (164.1 to 210.3) | 38.4 (33 to 44.3) |  | 39.7 (29.3 to 52.3) | 21.5 (14 to 30.7) |
| New Zealand | 221.7 (198.7 to 246) | 74.6 (65.2 to 85.2) |  | 38.4 (29.5 to 50.2) | 24.6 (16.6 to 34.6) |
| Nicaragua | 267.8 (211.7 to 338.6) | 223.6 (169.3 to 293.5) |  | 46.4 (35.3 to 59.2) | 27.6 (20.3 to 35.8) |
| Niger | 91.9 (51.9 to 150.9) | 95.7 (53 to 150.7) |  | 63.2 (42.8 to 92) | 55.4 (37.5 to 80) |
| Nigeria | 161.4 (117.1 to 218.9) | 176.4 (115.8 to 237.7) |  | 62.7 (47.4 to 83.3) | 62.6 (47.8 to 79.7) |
| Niue | 948.7 (604.3 to 1451.8) | 1002.5 (690.2 to 1505.7) |  | 109.8 (78.2 to 152.7) | 112.5 (81.7 to 151.7) |
| North Korea | 493.2 (309 to 754.7) | 630.8 (410.1 to 988.5) |  | 98.1 (69.9 to 133.4) | 101.5 (71.4 to 146.9) |
| North Macedonia | 418.9 (333.4 to 519.8) | 161.2 (117.8 to 217.1) |  | 107.3 (80.9 to 136.7) | 62.1 (44.1 to 84.7) |
| Northern Mariana Islands | 466.3 (288.9 to 679.4) | 498.3 (352.1 to 693.3) |  | 86.6 (60.7 to 118.6) | 65.9 (46 to 89.2) |
| Norway | 195.4 (185.9 to 206.3) | 29.1 (26.9 to 31.6) |  | 39.6 (31 to 50) | 20 (13.7 to 27.8) |
| Oman | 517.8 (351.3 to 728.6) | 282.6 (206.7 to 374.4) |  | 113.8 (81.1 to 152.8) | 95.4 (71.2 to 123.6) |
| Pakistan | 609.4 (431.5 to 782.1) | 906.5 (686.7 to 1175.1) |  | 50.4 (35.2 to 67.8) | 59.7 (43.4 to 81.4) |
| Palau | 1091.1 (727.4 to 1531.5) | 1457.3 (1065.2 to 1925.6) |  | 138.5 (98.5 to 189.6) | 162.3 (114.5 to 219) |
| Palestine | 607.8 (435.6 to 837.8) | 350.2 (272.8 to 442) |  | 135.9 (97.6 to 183.9) | 86.2 (65.5 to 110.8) |
| Panama | 181.2 (156.6 to 208) | 169.6 (134.7 to 210.5) |  | 49.1 (40.1 to 58.7) | 27.8 (21.9 to 34.8) |
| Papua New Guinea | 572.3 (315.3 to 919.4) | 646.9 (393 to 950.1) |  | 62.5 (41.4 to 91.5) | 56.2 (38.3 to 79.3) |
| Paraguay | 247.4 (190.8 to 313.1) | 167.5 (120.7 to 225.6) |  | 34.9 (27 to 44.7) | 21.8 (16.4 to 28.5) |
| Peru | 356.7 (269.3 to 465.3) | 217.1 (150.5 to 304.7) |  | 47.3 (34.9 to 62.3) | 32.1 (23.2 to 42.9) |
| Philippines | 1022.1 (928.3 to 1132.3) | 894.8 (760 to 1043.6) |  | 76.7 (63.3 to 91.4) | 83 (67.3 to 100.5) |
| Poland | 684.9 (664.3 to 705.4) | 81.8 (74.4 to 89.2) |  | 73.8 (62.4 to 87) | 39.9 (30.7 to 50.2) |
| Portugal | 221.9 (190.4 to 259.5) | 78.1 (66 to 91.4) |  | 85 (72.6 to 99.5) | 21.5 (16.2 to 28) |
| Puerto Rico | 292.8 (254.2 to 334.2) | 151.8 (122.9 to 184.4) |  | 34.4 (27.9 to 42.3) | 20 (14.5 to 26.5) |
| Qatar | 483.6 (348.9 to 643.9) | 171.5 (120.3 to 236.6) |  | 73.3 (51.9 to 97.6) | 50.7 (34.4 to 70.7) |
| Republic of Serbia | 420.9 (333 to 511.8) | 137.5 (102.3 to 176.6) |  | 99.3 (74.6 to 127.9) | 51.4 (35.4 to 71.1) |
| Republic of the Congo | 332.8 (197.2 to 518.1) | 277.8 (174.1 to 435.7) |  | 68 (46.4 to 95.6) | 59.5 (40.7 to 82.7) |
| Romania | 488.3 (435.1 to 550.5) | 258.1 (222.3 to 297.1) |  | 86 (66.6 to 107.4) | 54.1 (40.5 to 70.7) |
| Russia | 650.4 (626.9 to 670.8) | 373.1 (346.1 to 403.5) |  | 113 (97 to 131) | 86.9 (74 to 101.9) |
| Rwanda | 325.9 (203.5 to 501) | 162.9 (99.9 to 255) |  | 84.9 (57.5 to 123) | 39.2 (26.6 to 55.4) |
| Saint Kitts and Nevis | 477.1 (408.8 to 553) | 77.5 (50.4 to 112.5) |  | 131 (105.3 to 163.2) | 34.8 (26.1 to 44.9) |
| Saint Lucia | 235.4 (199.7 to 276.2) | 81.2 (63.9 to 102.1) |  | 80.8 (67 to 96) | 37.9 (30.1 to 46.6) |
| Saint Vincent and the Grenadines | 452.5 (390.4 to 517.3) | 180.3 (146.6 to 219.3) |  | 73 (60.3 to 87.6) | 44 (35.2 to 53.8) |
| Samoa | 622.7 (410.3 to 913.4) | 898.3 (587.3 to 1285.2) |  | 87.7 (61.9 to 118.7) | 92.5 (66.5 to 125.8) |
| San Marino | 81.5 (57.8 to 110.2) | 28.9 (16.1 to 44.9) |  | 32.6 (23.8 to 42.9) | 21.4 (14.6 to 29.9) |
| São Tomé and Principe | 163.9 (102.9 to 241.3) | 185.7 (105.5 to 300.2) |  | 102.8 (72 to 139.6) | 110.8 (73.9 to 161.8) |
| Saudi Arabia | 614.1 (423.2 to 848.4) | 706 (464.8 to 1036.4) |  | 140.1 (97.4 to 194.7) | 136.8 (92.9 to 194.6) |
| Senegal | 236.6 (157.6 to 335.8) | 195.2 (126.4 to 290.1) |  | 115.8 (83.5 to 158.8) | 86.9 (60.6 to 120.2) |
| Seychelles | 660.5 (518.4 to 825.4) | 340.8 (266.8 to 430.3) |  | 108.6 (79.7 to 143.2) | 72 (51.1 to 96.3) |
| Sierra Leone | 205.9 (129 to 307.1) | 266 (166.6 to 396.7) |  | 109.4 (77.1 to 153) | 109.6 (72.8 to 158.4) |
| Singapore | 364.3 (322.7 to 408.6) | 132.1 (116.1 to 148.7) |  | 50.1 (39 to 64.2) | 23.8 (15.6 to 34.3) |
| Slovakia | 515.5 (411.8 to 627.3) | 161.8 (123.3 to 207.1) |  | 75.9 (54.8 to 99.1) | 47.3 (32.7 to 64.7) |
| Slovenia | 189.5 (163.9 to 217.1) | 36.5 (29.9 to 43.9) |  | 48 (34.8 to 64) | 24.5 (15.5 to 36.3) |
| Solomon Islands | 1034.9 (535.3 to 1502.5) | 1146.6 (782.7 to 1582.7) |  | 80.8 (55.2 to 113.6) | 82.9 (57.3 to 114.5) |
| Somalia | 186.1 (113.1 to 299.9) | 220.4 (128.2 to 351.8) |  | 53.2 (34.3 to 78.9) | 44.9 (29.4 to 65.7) |
| South Africa | 398.8 (340.3 to 470.2) | 192.4 (165.4 to 226.6) |  | 104.3 (86.7 to 125.7) | 58 (47 to 70.8) |
| South Korea | 179.2 (126.3 to 241.8) | 57.8 (42.7 to 76.3) |  | 75.4 (56.2 to 96.7) | 34.6 (22.9 to 47.9) |
| South Sudan | 179.7 (111.7 to 276.8) | 254.3 (155.4 to 392.7) |  | 48.6 (32.6 to 68.2) | 46.2 (30.9 to 65.9) |
| Spain | 227.1 (195.5 to 261.2) | 75.5 (64.7 to 87) |  | 45 (36.5 to 55.5) | 18.5 (13.3 to 25.8) |
| Sri Lanka | 465.8 (359.7 to 592.7) | 334.5 (222.9 to 469.4) |  | 109.1 (83.1 to 140.2) | 77.7 (55.5 to 106) |
| Sudan | 1320.3 (893.7 to 1862.9) | 863 (496.2 to 1276.9) |  | 176.5 (105.8 to 282.5) | 155.9 (91.7 to 243.9) |
| Suriname | 525.4 (376.4 to 683) | 279.9 (198.2 to 383.6) |  | 69.7 (49.5 to 94) | 52.5 (38.5 to 70.5) |
| Sweden | 128.4 (114.6 to 143.7) | 25.9 (21.6 to 31) |  | 37.7 (29.2 to 48) | 29.8 (19.4 to 43.4) |
| Switzerland | 205.6 (177.9 to 235.4) | 33.9 (29 to 39.6) |  | 34.7 (27.2 to 44.3) | 14.8 (10 to 20.8) |
| Syria | 1967.1 (1537.3 to 2432.1) | 1013.4 (747.6 to 1375.2) |  | 239.9 (178.4 to 319.7) | 136.1 (99.8 to 182.2) |
| Tajikistan | 693.3 (560.1 to 834.8) | 375.6 (275.4 to 486.6) |  | 132.8 (98 to 177.6) | 79.9 (56.3 to 108) |
| Thailand | 267.3 (176.4 to 372.1) | 298.2 (203 to 422.1) |  | 60.8 (42.9 to 81.7) | 73.1 (51.9 to 97.6) |
| The Bahamas | 397 (336.9 to 462.7) | 196.6 (149.2 to 258.8) |  | 51.2 (41.2 to 63.3) | 36.8 (28.6 to 46.5) |
| Togo | 201.5 (136.1 to 289.8) | 209.5 (129.2 to 319.2) |  | 103.2 (74.8 to 138.3) | 91 (62.9 to 127.8) |
| Tokelau | 743.1 (467.7 to 1121.8) | 1006.3 (736.3 to 1383.8) |  | 92.5 (64.3 to 129.2) | 104.4 (76 to 139.1) |
| Tonga | 438 (316 to 579.8) | 527.5 (353.9 to 797.5) |  | 66.4 (46 to 90) | 66.1 (46.1 to 90.6) |
| Trinidad and Tobago | 487.8 (428.8 to 553.8) | 342.6 (258.2 to 452) |  | 82.4 (68.4 to 98.5) | 54.8 (42.5 to 68.7) |
| Tunisia | 507.1 (362.7 to 674.1) | 359.2 (234.7 to 511.5) |  | 89.3 (62.4 to 121.8) | 82 (55.2 to 115.7) |
| Turkey | 568.7 (415.7 to 747.1) | 221.8 (162.6 to 292.8) |  | 128.9 (95.4 to 168.9) | 62.9 (46.1 to 82.2) |
| Turkmenistan | 822.3 (713.7 to 930.4) | 598.6 (455.1 to 791.6) |  | 143.3 (108.8 to 179.3) | 172.4 (129.7 to 220.3) |
| Tuvalu | 1088.7 (756.8 to 1475.1) | 1368.5 (962.4 to 1821) |  | 118.7 (85.1 to 161.5) | 113.9 (81.7 to 155.5) |
| Uganda | 159.9 (97.1 to 241.5) | 223.5 (140.5 to 333.4) |  | 49.8 (34.2 to 69.4) | 43.8 (29.7 to 61) |
| Ukraine | 435 (354.6 to 527.1) | 557.5 (395.6 to 759.9) |  | 93.3 (70.7 to 119.8) | 93.1 (68.3 to 122.6) |
| United Arab Emirates | 416.2 (274.8 to 601.1) | 210.9 (138.3 to 295.9) |  | 113 (81.6 to 154.6) | 74.3 (54.1 to 99.8) |
| United Kingdom | 216.9 (211.6 to 222.2) | 74.9 (72.1 to 78.1) |  | 39.4 (32.2 to 47.6) | 20.2 (14.8 to 26.3) |
| United Republic of Tanzania | 221.5 (148.1 to 317.9) | 330.4 (206.5 to 482) |  | 47.6 (33.5 to 64.5) | 55.4 (37.5 to 76.3) |
| United States of America | 216 (209.5 to 223.2) | 151 (140.4 to 160) |  | 54.9 (41.5 to 70.2) | 48.1 (36.4 to 61.6) |
| United States Virgin Islands | 500.6 (359.5 to 675.7) | 376.6 (219.2 to 571) |  | 41.9 (30.2 to 57.4) | 33.2 (21.9 to 48.6) |
| Uruguay | 276.6 (240 to 316.4) | 114.8 (96.6 to 135.4) |  | 61.9 (49.2 to 77) | 30.8 (22.7 to 40.6) |
| Uzbekistan | 739.5 (647.9 to 834.8) | 640.5 (527.1 to 760.7) |  | 127.9 (106.6 to 152.4) | 97.7 (78.3 to 120.1) |
| Vanuatu | 1352.4 (865.7 to 1914.7) | 1597.4 (1103.4 to 2141.1) |  | 133.3 (93.6 to 181) | 134.7 (96.4 to 182.2) |
| Venezuela | 498.3 (442.8 to 561.8) | 445.5 (335.3 to 583.7) |  | 45.9 (37.4 to 55.6) | 35.8 (27.1 to 46.6) |
| Vietnam | 184.5 (122.4 to 269.7) | 163.8 (106 to 250.2) |  | 71 (50.3 to 96.9) | 65.7 (45.8 to 92.1) |
| Yemen | 759.7 (429.6 to 1205.8) | 564.1 (344.6 to 873.2) |  | 120.6 (71.7 to 186.1) | 119.9 (72 to 179.4) |
| Zambia | 174.5 (118.4 to 252.7) | 233 (138.4 to 359) |  | 52.7 (35.3 to 74.7) | 49 (33.2 to 68.7) |
| Zimbabwe | 75.7 (52.8 to 106.1) | 215.2 (133.3 to 326.5) |  | 38.7 (26.6 to 53) | 64.1 (43.9 to 90.1) |

| **Supplementary Table 7. Age standardized incidence number of ischemic heart disease vs ischemic stroke in youths and young Adults (15-39 years) at country level, 1990-2021, both sexes** | | | | | |
| --- | --- | --- | --- | --- | --- |
|  | **Ischemic heart disease (Incidence number, 95% UI)** | |  | **Ischemic stroke (Incidence number, 95% UI)** | |
|  | **Age standardized number in 1990** | **Age standardized number in 2021** |  | **Age standardized number in 1990** | **Age standardized number in 2021** |
| Afghanistan | 239.6 (119.1 to 393.1) | 1110.2 (585.4 to 1757.6) |  | 119.5 (72.5 to 186.5) | 461.7 (301 to 675.3) |
| Albania | 107.7 (57.4 to 172.9) | 69.6 (36.7 to 110.2) |  | 35.8 (19.1 to 59.2) | 22.6 (12.3 to 36.8) |
| Algeria | 856.3 (457 to 1347.5) | 2025.9 (1076.9 to 3212.8) |  | 398.2 (247.7 to 617.4) | 635 (402.8 to 938.9) |
| American Samoa | 1 (0.5 to 1.6) | 1 (0.5 to 1.5) |  | 0.5 (0.3 to 0.8) | 0.5 (0.3 to 0.7) |
| Andorra | 0.7 (0.3 to 1.2) | 0.7 (0.3 to 1.1) |  | 0.4 (0.2 to 0.7) | 0.4 (0.2 to 0.7) |
| Angola | 194 (94.2 to 320.1) | 564.1 (270.9 to 936.9) |  | 114.5 (61.5 to 188.2) | 324.4 (174.8 to 533.7) |
| Antigua and Barbuda | 2.1 (1.1 to 3.4) | 3.2 (1.7 to 5.1) |  | 0.5 (0.3 to 0.9) | 0.7 (0.3 to 1.2) |
| Argentina | 449.6 (216.1 to 760.9) | 632.8 (301 to 1056.1) |  | 263 (132.3 to 452.7) | 312.1 (150.1 to 535.1) |
| Armenia | 107.3 (55.4 to 170.1) | 102.6 (54.6 to 163.8) |  | 65.5 (40 to 100.7) | 38.2 (22 to 60.2) |
| Australia | 262.1 (133.3 to 426.4) | 288.8 (132.6 to 495.5) |  | 140.5 (83.3 to 213.2) | 153.2 (91 to 231.5) |
| Austria | 81.5 (37.6 to 139.8) | 79.7 (36.5 to 135.3) |  | 61.2 (32.3 to 103) | 50.7 (23.9 to 87.9) |
| Azerbaijan | 230.5 (119.3 to 372.3) | 385.1 (204.2 to 611.2) |  | 113 (63.3 to 183.9) | 132.8 (72.8 to 217.3) |
| Bahrain | 34.7 (18.4 to 54.1) | 95.3 (51.7 to 147.4) |  | 8.1 (4.6 to 12.9) | 18.9 (10.2 to 31.2) |
| Bangladesh | 2280.4 (1089.2 to 3763.4) | 4448.2 (2276 to 7276.6) |  | 839 (422.3 to 1416.5) | 1500.3 (801.7 to 2490) |
| Barbados | 9.5 (5.1 to 14.9) | 9.8 (5.3 to 15.4) |  | 2.6 (1.4 to 4.3) | 2.1 (1.1 to 3.5) |
| Belarus | 392.7 (213.8 to 621.6) | 325.4 (177 to 510.7) |  | 155.5 (87.6 to 254.6) | 103.1 (57.8 to 169.3) |
| Belgium | 141.4 (75.6 to 219.6) | 91.3 (41.7 to 156.5) |  | 77.4 (38.4 to 133) | 48.2 (22.2 to 83) |
| Belize | 5 (2.6 to 8) | 15.6 (8.1 to 24.6) |  | 1.2 (0.5 to 2.1) | 3.3 (1.7 to 5.6) |
| Benin | 75.7 (36.4 to 125.9) | 239.1 (116.5 to 398.5) |  | 59 (33.1 to 96.1) | 164.2 (94.2 to 263.4) |
| Bermuda | 2.5 (1.3 to 3.9) | 1.8 (1 to 2.9) |  | 0.6 (0.3 to 1) | 0.4 (0.2 to 0.6) |
| Bhutan | 14.5 (7.2 to 23.7) | 24.2 (12.3 to 39) |  | 4.7 (2.2 to 8.2) | 6.6 (3.2 to 11.5) |
| Bolivia | 122.8 (61.5 to 202) | 278.8 (140.9 to 456) |  | 54.5 (29.3 to 89.4) | 86.6 (42.4 to 148) |
| Bosnia and Herzegovina | 153.9 (82.2 to 244.2) | 88.2 (47.4 to 140.1) |  | 95.8 (63.7 to 142) | 51.1 (33.9 to 74.6) |
| Botswana | 24.3 (11.7 to 40.3) | 70.3 (35.4 to 115.6) |  | 13.5 (7.1 to 22.5) | 30.2 (16.5 to 49.4) |
| Brazil | 3191.4 (1607.2 to 5217.4) | 4278.2 (2165.8 to 6941.9) |  | 1578 (853.1 to 2593.7) | 1411.3 (700.6 to 2431.6) |
| Brunei | 2.5 (1 to 4.4) | 4.2 (1.7 to 7.4) |  | 3.8 (2 to 6.2) | 4.4 (2.2 to 7.5) |
| Bulgaria | 227.6 (121.6 to 362.3) | 166.9 (87.8 to 261.8) |  | 88.8 (51.3 to 139.7) | 66.3 (40.9 to 100.4) |
| Burkina Faso | 129.2 (61.5 to 216.1) | 365.2 (176.1 to 608.2) |  | 94.7 (50.5 to 159.3) | 230.6 (126.2 to 375) |
| Burundi | 88.7 (42.5 to 147.3) | 236.4 (113 to 391.5) |  | 75.5 (44.2 to 119.1) | 141.3 (78 to 229.5) |
| Cabo Verde | 5.3 (2.5 to 8.8) | 14.6 (7.3 to 24) |  | 4.7 (2.7 to 7.6) | 9.3 (5.3 to 15) |
| Cambodia | 108.8 (48.2 to 186) | 235.2 (110.9 to 396.1) |  | 76.6 (37.8 to 133.7) | 149 (79 to 245.8) |
| Cameroon | 143.8 (68.7 to 238.3) | 570.2 (274.2 to 946.2) |  | 112.6 (62.1 to 185.7) | 436.7 (261.8 to 685.6) |
| Canada | 521.9 (256.2 to 859.9) | 470.1 (234.7 to 776) |  | 304 (153.6 to 517.1) | 276.5 (192 to 385.3) |
| Central African Republic | 50.3 (23.9 to 83.6) | 108.7 (52.6 to 179.7) |  | 27.5 (14.3 to 46) | 54.9 (29.9 to 88.6) |
| Chad | 97.2 (47 to 162.5) | 278 (134.3 to 461.1) |  | 75.1 (42.3 to 121.1) | 216.3 (129.6 to 333.9) |
| Chile | 96.2 (36.9 to 175.7) | 137.6 (60.5 to 237.7) |  | 121.4 (59.5 to 208.7) | 134.1 (63.9 to 232.3) |
| China | 34434.5 (17950.2 to 54715.2) | 40556.1 (21930.8 to 63295.2) |  | 10531.1 (5509.2 to 17573.8) | 11379.3 (6324.2 to 18493.7) |
| Colombia | 837.6 (435.9 to 1360.9) | 1293 (670.1 to 2082.8) |  | 315.4 (166.1 to 523.7) | 318.2 (152.6 to 547.3) |
| Comoros | 6.8 (3.3 to 11.5) | 14.9 (7.3 to 24.5) |  | 5.7 (3.2 to 9.2) | 9.2 (5 to 15.1) |
| Cook Islands | 0.3 (0.2 to 0.6) | 0.3 (0.2 to 0.5) |  | 0.2 (0.1 to 0.3) | 0.2 (0.1 to 0.3) |
| Costa Rica | 90.4 (47.8 to 143.9) | 151.9 (80 to 241.8) |  | 27.8 (13.9 to 47.5) | 36.8 (17.9 to 62.8) |
| Croatia | 128.5 (68.9 to 204.7) | 84.9 (43.7 to 133.2) |  | 45.7 (23.6 to 76.6) | 23.5 (11.9 to 40) |
| Cuba | 388.8 (204.7 to 623) | 336.9 (178.7 to 527.7) |  | 103.5 (52.8 to 177.1) | 71.3 (35.8 to 119.7) |
| Cyprus | 5.2 (2.1 to 9.4) | 9.1 (3.6 to 16.7) |  | 4.2 (1.8 to 7.6) | 5.4 (2.2 to 10.1) |
| Czechia | 389.2 (219.1 to 604.6) | 323 (178 to 508.3) |  | 118.4 (65.9 to 193.6) | 66.9 (33.2 to 114.6) |
| Democratic Republic of the Congo | 661.1 (321 to 1102.4) | 1671.9 (804 to 2805.6) |  | 373.5 (191.5 to 630.9) | 833.7 (441.7 to 1391.3) |
| Denmark | 21.8 (11.6 to 36.6) | 32.7 (13.3 to 57.9) |  | 28.8 (17.9 to 44.4) | 25.5 (11.2 to 45) |
| Djibouti | 7.3 (3.5 to 12.3) | 29.9 (14.7 to 49.4) |  | 5.1 (2.7 to 8.4) | 17.3 (9.6 to 27.9) |
| Dominica | 2.2 (1.2 to 3.6) | 2.3 (1.2 to 3.6) |  | 0.6 (0.3 to 1) | 0.5 (0.3 to 0.8) |
| Dominican Republic | 215.6 (113.5 to 345.3) | 397.2 (210.4 to 624.4) |  | 65.6 (35.6 to 108) | 117.4 (69 to 181.6) |
| East Timor | 9.5 (4.4 to 16.1) | 15.3 (6.8 to 26.1) |  | 6.4 (3.1 to 11.1) | 11.8 (6.2 to 19.5) |
| Ecuador | 203.2 (101.2 to 334.3) | 436.6 (222.8 to 711.1) |  | 106.3 (59.9 to 171) | 158.8 (87.1 to 257.6) |
| Egypt | 1877.6 (979.9 to 2981) | 4563 (2435.3 to 7128.9) |  | 774 (491.8 to 1148.6) | 1773.6 (1159.2 to 2621.9) |
| El Salvador | 104.8 (51.7 to 171.1) | 155.4 (80.5 to 249.8) |  | 40 (20.1 to 67.3) | 40 (18.8 to 69.1) |
| Equatorial Guinea | 7.2 (3.4 to 11.9) | 34.1 (16.4 to 56.5) |  | 4.4 (2.4 to 7.3) | 16.8 (8.5 to 28.2) |
| Eritrea | 51.7 (24.3 to 86.3) | 124.6 (60.2 to 207.1) |  | 36.6 (19.6 to 60.7) | 78.4 (43.6 to 126.1) |
| Estonia | 70.4 (46.5 to 100.4) | 53.2 (29.3 to 83.3) |  | 20.7 (12.8 to 31.3) | 11.8 (5.9 to 19.9) |
| eSwatini | 13.3 (6.3 to 22) | 28.2 (13.9 to 46.5) |  | 6.8 (3.3 to 11.9) | 11.8 (6.1 to 19.7) |
| Ethiopia | 819.6 (388.6 to 1352.1) | 1996.6 (952.1 to 3326.3) |  | 496.7 (258.8 to 827.5) | 867.1 (417.2 to 1488.6) |
| Federated States of Micronesia | 2 (1 to 3.3) | 2.2 (1.1 to 3.6) |  | 1.3 (0.8 to 2.1) | 1.5 (1 to 2.3) |
| Fiji | 17.7 (8.9 to 29) | 22.1 (11.3 to 35.9) |  | 10.9 (6.5 to 17.2) | 12.5 (7.7 to 19.1) |
| Finland | 119.5 (72.6 to 177.3) | 50.7 (23.6 to 85.5) |  | 43 (25.8 to 66.2) | 34 (18.8 to 54) |
| France | 821 (483.6 to 1242.7) | 536.5 (252.3 to 922.3) |  | 298.7 (180.2 to 452.5) | 261.5 (161.8 to 394.5) |
| Gabon | 17.3 (8.4 to 28.5) | 36.3 (17.5 to 59.7) |  | 10.8 (5.7 to 18.2) | 20 (10.7 to 33.4) |
| Gambia | 17.8 (8.5 to 29.5) | 50.3 (24.7 to 82.5) |  | 15.5 (9.1 to 24.7) | 39.1 (23.9 to 59.8) |
| Georgia | 179.2 (95 to 283.4) | 112.7 (60.1 to 179.2) |  | 94 (54.8 to 149.3) | 48.1 (29.6 to 74.5) |
| Germany | 1450.6 (894.5 to 2138.3) | 897 (444.6 to 1514.9) |  | 867.4 (516.9 to 1348.3) | 527.8 (275.6 to 877.5) |
| Ghana | 272.9 (133.8 to 450.6) | 765.2 (379.1 to 1262.4) |  | 334.9 (216 to 495.2) | 896.4 (605.6 to 1302.5) |
| Greece | 71.8 (30.2 to 129.3) | 53.1 (21.9 to 94.3) |  | 73.7 (35.1 to 126.7) | 44.3 (20.4 to 77) |
| Greenland | 1.2 (0.6 to 2) | 0.9 (0.4 to 1.4) |  | 0.8 (0.5 to 1.4) | 0.5 (0.2 to 0.8) |
| Grenada | 2.6 (1.4 to 4.2) | 3.5 (1.9 to 5.6) |  | 1 (0.6 to 1.5) | 0.9 (0.5 to 1.4) |
| Guam | 2.8 (1.4 to 4.7) | 3 (1.5 to 4.9) |  | 1.4 (0.8 to 2.4) | 1.8 (1.1 to 2.8) |
| Guatemala | 139.8 (68.7 to 230.9) | 371.4 (186.8 to 606.1) |  | 54.7 (27.9 to 89.5) | 107.4 (54.3 to 180.3) |
| Guinea | 91.4 (43.9 to 149.1) | 235.7 (113.9 to 391.3) |  | 73.2 (42.7 to 117.3) | 196.5 (119.4 to 300.5) |
| Guinea-Bissau | 16.9 (8.2 to 28.1) | 42.6 (20.8 to 70) |  | 16.9 (10.5 to 26) | 36.9 (23.5 to 54.2) |
| Guyana | 27.5 (14.4 to 44.2) | 26.1 (13.7 to 41.2) |  | 9.9 (6.1 to 14.9) | 7.8 (4.9 to 11.9) |
| Haiti | 205.7 (108.4 to 330.2) | 524.7 (277.7 to 831.2) |  | 56.5 (32.1 to 90.6) | 121 (72.2 to 185.8) |
| Honduras | 100.9 (50.8 to 162.8) | 274.8 (141.9 to 450.4) |  | 32.8 (16.3 to 55.1) | 70.5 (33.1 to 124.3) |
| Hungary | 395.9 (220 to 618.8) | 231.4 (125.2 to 362.5) |  | 170 (110.3 to 250.9) | 69.7 (37.9 to 114.8) |
| Iceland | 3.6 (1.8 to 6) | 3.4 (1.5 to 5.7) |  | 2.1 (1 to 3.7) | 1.8 (0.8 to 3.2) |
| India | 24601.6 (12931.4 to 39600.7) | 50604.3 (26796.2 to 80461.6) |  | 7049.1 (3511.8 to 12058.3) | 12727.7 (6603.2 to 21346.2) |
| Indonesia | 2866.6 (1360.2 to 4777.5) | 4875 (2369.4 to 7967.9) |  | 2737.8 (1568.5 to 4423.4) | 3670.8 (2135.4 to 5756.5) |
| Iran | 2010.7 (1056.1 to 3179.7) | 4957.9 (2681.7 to 7693.4) |  | 938.4 (577.6 to 1442.1) | 1224.7 (759.1 to 1871.5) |
| Iraq | 682.3 (364.9 to 1096.1) | 2043.7 (1092.2 to 3236) |  | 299.8 (187.9 to 454.4) | 620.6 (378.3 to 959.7) |
| Ireland | 35.2 (15.6 to 60.5) | 36.5 (15.6 to 64.2) |  | 24.9 (11.8 to 43.5) | 19.1 (7.9 to 35.8) |
| Israel | 45.3 (20.5 to 77.8) | 69.9 (29.5 to 122.9) |  | 39.4 (18.7 to 68) | 49.4 (22.1 to 90) |
| Italy | 782.6 (367.2 to 1317.4) | 539.2 (259.9 to 892) |  | 436.1 (194.4 to 796.4) | 193.1 (85.2 to 346.6) |
| Ivory Coast | 223.6 (110.7 to 375.2) | 610.8 (302 to 1000.6) |  | 204.9 (124.8 to 315.6) | 443.4 (275.8 to 682.3) |
| Jamaica | 71.4 (37.3 to 114.6) | 108.7 (57.9 to 171.9) |  | 18.6 (9.4 to 31.1) | 22.2 (11.5 to 38) |
| Japan | 867.1 (353 to 1554.4) | 634.5 (259.6 to 1136.5) |  | 874.5 (388.1 to 1580.4) | 703.4 (338.1 to 1223.3) |
| Jordan | 130 (68.3 to 206.9) | 611.5 (322 to 966.5) |  | 62.7 (36.7 to 102.1) | 187.7 (105.5 to 305.5) |
| Kazakhstan | 603.5 (322.6 to 964.6) | 700.3 (379.2 to 1117.8) |  | 403.5 (261.5 to 594.9) | 319 (200.1 to 492.5) |
| Kenya | 393.9 (190.5 to 651.4) | 1094.5 (536 to 1800.9) |  | 240.8 (124.3 to 407.9) | 545 (286.6 to 909.6) |
| Kiribati | 1.7 (0.8 to 2.7) | 2.9 (1.5 to 4.7) |  | 1.5 (1 to 2.2) | 2.7 (1.9 to 3.7) |
| Kuwait | 115.6 (62.3 to 181.7) | 350.8 (189 to 544.6) |  | 30.9 (17.8 to 50.5) | 78.7 (44.6 to 127.8) |
| Kyrgyzstan | 129.8 (67.1 to 207.8) | 193.3 (100.9 to 309.1) |  | 84.6 (52.8 to 130.3) | 81.6 (46.8 to 128.8) |
| Laos | 50 (22.7 to 84.6) | 107.2 (50 to 179.2) |  | 38.7 (20.8 to 63.9) | 81.3 (46.4 to 130.1) |
| Latvia | 68.3 (35.5 to 109) | 41.5 (22 to 65.2) |  | 32.5 (18.2 to 52.4) | 14.7 (7.8 to 24.6) |
| Lebanon | 103.5 (53.8 to 167.7) | 306.1 (162.8 to 475.5) |  | 43.2 (26.3 to 66.7) | 96.7 (61.2 to 145.9) |
| Lesotho | 23.9 (11.4 to 40) | 41.8 (20.4 to 68.8) |  | 11.3 (5.4 to 20) | 16.9 (8.7 to 28.1) |
| Liberia | 45.3 (22.3 to 75.4) | 117.7 (57.9 to 194.1) |  | 36.2 (21.2 to 57.6) | 79.9 (47.3 to 126.7) |
| Libya | 143.1 (75.3 to 228.5) | 379.3 (200.3 to 592.1) |  | 54.7 (33.4 to 84.2) | 132.8 (89.3 to 191.1) |
| Lithuania | 107 (58.6 to 164.8) | 58.6 (30.8 to 92.3) |  | 56.1 (31.9 to 89.4) | 27 (14.8 to 44.2) |
| Luxembourg | 3 (1.2 to 5.2) | 4.2 (1.7 to 7.5) |  | 3.2 (1.6 to 5.3) | 2.7 (1.2 to 4.9) |
| Madagascar | 188.7 (89.6 to 314.5) | 523.2 (250.8 to 874.3) |  | 187.7 (114 to 290.9) | 450.3 (274.4 to 696.2) |
| Malawi | 165.2 (79.7 to 276.7) | 380.2 (185.6 to 629.8) |  | 105.4 (56.6 to 176.1) | 225.3 (126.3 to 366.7) |
| Malaysia | 261.6 (122.8 to 440) | 591 (288.5 to 974.8) |  | 213.2 (114.9 to 350.5) | 407.9 (225.8 to 663.5) |
| Maldives | 2.4 (1.1 to 4) | 11.7 (5.8 to 19.1) |  | 2.6 (1.5 to 4.1) | 6.4 (3.4 to 10.7) |
| Mali | 131.7 (63 to 218.2) | 375.5 (176.7 to 626.1) |  | 103.5 (58.9 to 167) | 256.7 (145.4 to 415.3) |
| Malta | 2.9 (1.2 to 5) | 2.6 (1.1 to 4.6) |  | 2.7 (1.3 to 4.6) | 1.8 (0.8 to 3.2) |
| Marshall Islands | 0.8 (0.4 to 1.3) | 1.3 (0.6 to 2.1) |  | 0.5 (0.3 to 0.8) | 0.9 (0.6 to 1.3) |
| Mauritania | 37.9 (18.6 to 62.9) | 84.7 (41.6 to 140.1) |  | 37.2 (22.4 to 58.6) | 59.7 (34.2 to 96.2) |
| Mauritius | 18 (8.5 to 30.1) | 16.5 (7.8 to 27.4) |  | 16.5 (9.7 to 26) | 10.5 (5.6 to 17.5) |
| Mexico | 2324.2 (1198.2 to 3726) | 3996.7 (2114.9 to 6348.7) |  | 979.1 (529.5 to 1618.4) | 1062.7 (545.6 to 1787.6) |
| Moldova | 152.6 (81.9 to 246.1) | 132.1 (71.5 to 209.8) |  | 56.3 (31 to 91.7) | 40.7 (23.3 to 65.8) |
| Monaco | 0.3 (0.1 to 0.4) | 0.2 (0.1 to 0.4) |  | 0.2 (0.1 to 0.3) | 0.2 (0.1 to 0.3) |
| Mongolia | 66.7 (34.4 to 109) | 122.8 (65.6 to 197.4) |  | 28.6 (16.1 to 47.2) | 49.6 (29.8 to 78.8) |
| Montenegro | 22.6 (12 to 35.8) | 19.2 (10.5 to 30.6) |  | 5.7 (2.8 to 9.8) | 4.6 (2.3 to 8) |
| Morocco | 992.9 (525 to 1577.7) | 1680.8 (898.3 to 2634.6) |  | 372.5 (226.6 to 579.6) | 523.2 (327.1 to 794) |
| Mozambique | 226.2 (110.6 to 374.5) | 551.7 (269.2 to 918.5) |  | 136.7 (71.5 to 227.2) | 365 (213.4 to 585.3) |
| Myanmar | 572.6 (265.4 to 958.4) | 731.6 (346 to 1231.3) |  | 447.3 (242.6 to 729.2) | 556.5 (312.4 to 897.4) |
| Namibia | 26.8 (13 to 44.6) | 54.5 (27 to 89.9) |  | 14.5 (7.4 to 24.7) | 24 (12.1 to 40.7) |
| Nauru | 0.2 (0.1 to 0.3) | 0.3 (0.1 to 0.4) |  | 0.2 (0.1 to 0.3) | 0.3 (0.2 to 0.4) |
| Nepal | 432.1 (219.4 to 701.5) | 768.7 (385.9 to 1250) |  | 132 (64.4 to 231.8) | 220 (101.7 to 384.1) |
| Netherlands | 210.5 (102.4 to 356.6) | 147.3 (67.9 to 249.9) |  | 144.5 (72.7 to 248) | 88.1 (40.9 to 156.9) |
| New Zealand | 78 (38 to 130.5) | 61.2 (33.1 to 97.6) |  | 26.8 (12.1 to 47.9) | 29.4 (13.7 to 52) |
| Nicaragua | 80.2 (40.7 to 131.2) | 180.7 (93.6 to 291.1) |  | 29.6 (14.7 to 50.9) | 46.5 (22.1 to 80.4) |
| Niger | 118.2 (56.6 to 194) | 360.3 (168.7 to 601.2) |  | 98.8 (56 to 156.9) | 267.3 (151.2 to 429.4) |
| Nigeria | 1659.4 (816.6 to 2736.4) | 4643.7 (2310.3 to 7592.8) |  | 1076.8 (583.3 to 1797.7) | 2710.5 (1516.4 to 4429.8) |
| Niue | 0 (0 to 0.1) | 0 (0 to 0.1) |  | 0 (0 to 0) | 0 (0 to 0) |
| North Korea | 511.6 (262.4 to 818.5) | 725.5 (376.7 to 1157.3) |  | 223 (125.3 to 357) | 303.8 (182.7 to 472.9) |
| North Macedonia | 67.1 (36.1 to 106.7) | 69.7 (37.4 to 110.3) |  | 27.9 (16.6 to 43.8) | 23.2 (13.5 to 36.6) |
| Northern Mariana Islands | 1.3 (0.6 to 2.1) | 1 (0.5 to 1.6) |  | 0.6 (0.3 to 1) | 0.4 (0.2 to 0.6) |
| Norway | 56.6 (27.5 to 94.1) | 52.6 (24.4 to 88.9) |  | 41.4 (19.3 to 73.9) | 25.8 (11.6 to 45.8) |
| Oman | 84.5 (45.1 to 133.6) | 351 (190.9 to 543.9) |  | 31 (19.3 to 47.4) | 97.6 (63.1 to 145.1) |
| Pakistan | 3067.5 (1598.8 to 4866.1) | 8951.7 (4795.4 to 14021.5) |  | 1016.3 (531.1 to 1711.4) | 2919.7 (1667.4 to 4689.7) |
| Palau | 0.3 (0.2 to 0.5) | 0.4 (0.2 to 0.6) |  | 0.2 (0.1 to 0.4) | 0.3 (0.2 to 0.4) |
| Palestine | 63 (32.5 to 100.6) | 205.5 (107.6 to 327.2) |  | 23.7 (13.5 to 38.5) | 60.9 (35.2 to 98.3) |
| Panama | 58.1 (29.5 to 95.1) | 108.2 (55.9 to 176.1) |  | 18.9 (9.3 to 32.5) | 26.3 (12.3 to 45.7) |
| Papua New Guinea | 73.9 (35.7 to 122) | 214.7 (103.8 to 351.1) |  | 27.2 (12.4 to 48.5) | 65.4 (30.2 to 116.5) |
| Paraguay | 65.4 (31.7 to 109.8) | 141 (70.3 to 232) |  | 26.8 (12.5 to 46.7) | 48.4 (22.4 to 85.9) |
| Peru | 411 (200.6 to 674.8) | 892.2 (458.3 to 1460.5) |  | 179.6 (92.8 to 300.4) | 294.9 (150.5 to 499) |
| Philippines | 860.5 (401 to 1445.7) | 1637 (772.7 to 2728.6) |  | 518.2 (244.7 to 906.9) | 1281.4 (731.2 to 2033.4) |
| Poland | 1626.3 (835.1 to 2625.7) | 809.7 (426.2 to 1277.5) |  | 411.6 (223.7 to 679.3) | 223.6 (112.5 to 381.5) |
| Portugal | 56.8 (21.7 to 104.7) | 46.4 (17.5 to 84.7) |  | 75.9 (40.1 to 125.8) | 32.5 (13.6 to 60.3) |
| Puerto Rico | 124.5 (65.1 to 196.2) | 100.1 (53.2 to 156.6) |  | 25.7 (12.2 to 44.8) | 18 (8.4 to 32.1) |
| Qatar | 35.1 (18.7 to 54.7) | 274.2 (147.8 to 428.3) |  | 9.9 (6 to 15.1) | 56.5 (30.6 to 94) |
| Republic of Serbia | 434.7 (252.3 to 662.4) | 372 (204.5 to 580.1) |  | 113.4 (65.2 to 180.2) | 69.8 (37.8 to 114.4) |
| Republic of the Congo | 43.7 (21 to 72.6) | 120.1 (59.6 to 197.2) |  | 30.3 (16.7 to 49.8) | 64.6 (35.4 to 104.3) |
| Romania | 717.6 (384.6 to 1137.5) | 472.7 (255.7 to 746.3) |  | 264.1 (147.8 to 424.1) | 131.2 (70.4 to 216.6) |
| Russia | 9338.8 (5217.3 to 14464.5) | 8854.3 (4988.7 to 13630.4) |  | 2433.6 (1421.1 to 3839.8) | 1730.3 (1028.9 to 2657.2) |
| Rwanda | 111.3 (53.6 to 187.1) | 242.3 (114.7 to 405.6) |  | 98 (56.1 to 157.1) | 139 (72.1 to 234.2) |
| Saint Kitts and Nevis | 1.4 (0.7 to 2.3) | 2.2 (1.2 to 3.5) |  | 0.7 (0.5 to 1) | 0.5 (0.3 to 0.8) |
| Saint Lucia | 4.5 (2.3 to 7.2) | 6.5 (3.5 to 10.3) |  | 1.4 (0.8 to 2.3) | 1.4 (0.8 to 2.3) |
| Saint Vincent and the Grenadines | 3.3 (1.7 to 5.3) | 3.6 (1.9 to 5.8) |  | 1.3 (0.8 to 2) | 0.8 (0.5 to 1.4) |
| Samoa | 3.1 (1.5 to 5.1) | 4.3 (2.1 to 7) |  | 1.8 (1 to 2.9) | 2.8 (1.7 to 4.2) |
| San Marino | 0.2 (0.1 to 0.4) | 0.2 (0.1 to 0.4) |  | 0.2 (0.1 to 0.3) | 0.1 (0.1 to 0.2) |
| São Tomé and Principe | 1.8 (0.8 to 2.9) | 4.6 (2.2 to 7.6) |  | 1.9 (1.1 to 2.9) | 5 (3.2 to 7.4) |
| Saudi Arabia | 620.7 (328.1 to 976.7) | 2914 (1592.2 to 4560.4) |  | 190.8 (109.7 to 301.7) | 621.6 (398.4 to 925) |
| Senegal | 136.7 (67.5 to 222.6) | 335 (167.2 to 549.2) |  | 123.9 (73.4 to 198.3) | 250.6 (147.7 to 389.4) |
| Seychelles | 1 (0.5 to 1.7) | 1.5 (0.7 to 2.5) |  | 0.9 (0.5 to 1.5) | 1.1 (0.6 to 1.7) |
| Sierra Leone | 85 (42.9 to 138.5) | 183.3 (90.5 to 305.1) |  | 69 (40.9 to 108.4) | 151.2 (95.1 to 229.2) |
| Singapore | 49 (22.3 to 83.7) | 59.7 (27.8 to 101.1) |  | 40.4 (20.5 to 69.7) | 40.4 (18.3 to 72.1) |
| Slovakia | 169.3 (90.1 to 269.7) | 135.5 (72.2 to 210.5) |  | 68.1 (38.7 to 109.7) | 47.9 (26.3 to 77.8) |
| Slovenia | 66.7 (36.6 to 105.2) | 48.7 (26.3 to 76.4) |  | 17.9 (9.1 to 30.8) | 9.4 (4.4 to 16.6) |
| Solomon Islands | 5.7 (2.8 to 9.5) | 14.3 (7.1 to 23.4) |  | 3.6 (2.1 to 5.7) | 9.4 (5.9 to 14.1) |
| Somalia | 138.2 (67.2 to 230.8) | 378.6 (184.1 to 625.4) |  | 92.6 (51 to 153.3) | 233.8 (130.6 to 380) |
| South Africa | 1090.5 (565.8 to 1760.4) | 1921.9 (1007.4 to 3069.7) |  | 598.8 (342 to 961.7) | 684.4 (381.3 to 1108.2) |
| South Korea | 355.3 (141.3 to 638.8) | 263.3 (98.2 to 487) |  | 685.8 (374.2 to 1103.4) | 392.7 (191.6 to 677.5) |
| South Sudan | 88.3 (41.7 to 148.3) | 149.1 (71 to 249.9) |  | 59.6 (30.6 to 99.9) | 90.3 (46 to 153.2) |
| Spain | 401.1 (199 to 663.3) | 329.1 (148 to 565.8) |  | 279.5 (146.8 to 464.7) | 167.6 (91.3 to 274.4) |
| Sri Lanka | 245.2 (114.3 to 413.9) | 278.4 (131.3 to 462.7) |  | 195 (107.2 to 319.7) | 200.5 (109.9 to 329.4) |
| Sudan | 706.8 (371.9 to 1127.6) | 1949.2 (1047.3 to 3057.5) |  | 259 (157.2 to 399.5) | 688.4 (443.9 to 1014.2) |
| Suriname | 13 (6.7 to 20.9) | 20.8 (11 to 32.9) |  | 3.6 (2 to 5.9) | 4.8 (2.7 to 7.7) |
| Sweden | 70.7 (50.5 to 93.5) | 99.8 (45 to 169.8) |  | 54.2 (25.6 to 95.6) | 67.9 (32.4 to 118.4) |
| Switzerland | 79.7 (42.5 to 126.5) | 62.8 (27.3 to 109.3) |  | 45.4 (21.9 to 77.8) | 32.5 (13.7 to 59.1) |
| Syria | 385.8 (199.5 to 616.8) | 462.9 (245.7 to 747.3) |  | 252.4 (171.4 to 359.3) | 199.5 (131.1 to 291.4) |
| Tajikistan | 144.1 (74.1 to 231.7) | 325.5 (170.4 to 520) |  | 72.3 (40.4 to 116.7) | 123.1 (68.5 to 196.6) |
| Thailand | 780 (360.9 to 1310) | 729.8 (344 to 1203.8) |  | 616.6 (317.7 to 1037) | 538.6 (305.7 to 861.3) |
| The Bahamas | 9.9 (5.2 to 15.6) | 14.8 (8 to 23.6) |  | 2.6 (1.4 to 4.2) | 3.3 (1.8 to 5.3) |
| Togo | 62.2 (30.3 to 104) | 169.2 (83.3 to 278.7) |  | 56.7 (33.6 to 91) | 133.8 (82.8 to 203.4) |
| Tokelau | 0 (0 to 0) | 0 (0 to 0) |  | 0 (0 to 0) | 0 (0 to 0) |
| Tonga | 1.6 (0.8 to 2.7) | 2 (1 to 3.2) |  | 0.8 (0.4 to 1.3) | 0.9 (0.5 to 1.5) |
| Trinidad and Tobago | 49.4 (26.2 to 79.1) | 61 (32.8 to 95.5) |  | 17.6 (11.1 to 26.6) | 13.8 (7.8 to 22) |
| Tunisia | 286.1 (152 to 460.3) | 503.9 (267.9 to 799) |  | 91.3 (49.5 to 148.5) | 136.4 (81.3 to 214.7) |
| Turkey | 2319.2 (1233.4 to 3681.5) | 3308.7 (1758.9 to 5245.2) |  | 911 (540.6 to 1464.9) | 916.7 (501.3 to 1519.4) |
| Turkmenistan | 107.5 (55.4 to 171.8) | 183.8 (98 to 291.6) |  | 59.4 (35 to 92.7) | 118.5 (79.9 to 171.3) |
| Tuvalu | 0.2 (0.1 to 0.3) | 0.3 (0.1 to 0.4) |  | 0.1 (0.1 to 0.2) | 0.2 (0.1 to 0.2) |
| Uganda | 251.6 (119.3 to 416.8) | 668.8 (315.3 to 1125.5) |  | 181.6 (93.9 to 312.4) | 437.3 (226.8 to 725.3) |
| Ukraine | 2005.4 (1088.4 to 3117.4) | 1731.8 (957.3 to 2677.7) |  | 825 (467.3 to 1339) | 674 (418 to 1033.3) |
| United Arab Emirates | 143.8 (76.2 to 225.8) | 932.1 (497.5 to 1481.5) |  | 48.1 (30 to 74.1) | 230.8 (149.9 to 339.9) |
| United Kingdom | 679.5 (304.4 to 1175.5) | 697 (333.4 to 1166.1) |  | 376.5 (178.7 to 651.3) | 301.4 (151 to 512.5) |
| United Republic of Tanzania | 375.7 (177.8 to 633.3) | 1111.7 (546 to 1839.9) |  | 255.4 (130.3 to 432.7) | 734.2 (411.8 to 1187.2) |
| United States of America | 6462.3 (2951.9 to 11045.7) | 4902.4 (2471.5 to 7898.5) |  | 2487.8 (1210.8 to 4300.6) | 2508.6 (1252.7 to 4273.6) |
| United States Virgin Islands | 3.3 (1.7 to 5.3) | 2.3 (1.2 to 3.6) |  | 0.8 (0.4 to 1.3) | 0.4 (0.2 to 0.8) |
| Uruguay | 28.1 (12.4 to 48) | 30.1 (13.4 to 52.2) |  | 37.7 (21.4 to 60) | 21.5 (10.5 to 36.9) |
| Uzbekistan | 566.5 (293.5 to 911.5) | 1196.6 (639.7 to 1903.8) |  | 350.5 (206.8 to 549.9) | 557 (340.3 to 847.5) |
| Vanuatu | 3.1 (1.6 to 5.2) | 7.3 (3.7 to 11.9) |  | 2.1 (1.3 to 3.2) | 5.6 (3.6 to 8.1) |
| Venezuela | 546.6 (284.8 to 878.7) | 720.2 (379.3 to 1146) |  | 156.9 (75.3 to 272.6) | 171.1 (83.2 to 293.1) |
| Vietnam | 835.9 (382.1 to 1416.9) | 1413.2 (667.6 to 2338) |  | 649.4 (327.3 to 1103.6) | 1036.3 (584.5 to 1659.6) |
| Yemen | 425.4 (225.3 to 684) | 1385.4 (731.8 to 2172.6) |  | 137.8 (79.5 to 216.9) | 431.5 (269 to 654.1) |
| Zambia | 116 (53.5 to 195.6) | 346.2 (164.8 to 572) |  | 79 (42 to 132) | 203.2 (110.2 to 331.4) |
| Zimbabwe | 183.5 (87.8 to 307.3) | 352 (173.6 to 580.2) |  | 90.4 (46.8 to 149.8) | 173.7 (98.3 to 279.2) |

| **Supplementary Table 8. Age standardized prevalence number of ischemic heart disease vs ischemic stroke in youths and young Adults (15-39 years) at country level, 1990-2021, both sexes** | | | | | |
| --- | --- | --- | --- | --- | --- |
|  | **Ischemic heart disease (Prevalence number, 95% UI)** | |  | **Ischemic stroke (Prevalence number, 95% UI)** | |
|  | **Age standardized number in 1990** | **Age standardized number in 2021** |  | **Age standardized number in 1990** | **Age standardized number in 2021** |
| Afghanistan | 971.5 (805.7 to 1158.9) | 4664.2 (3686.9 to 5843.5) |  | 1805 (1609.8 to 2006.9) | 6453 (5893.7 to 7037.9) |
| Albania | 624.2 (524.9 to 738.6) | 425.3 (338.1 to 524.9) |  | 581.2 (522.3 to 645.1) | 367.9 (334.9 to 405.2) |
| Algeria | 4033.8 (3362.6 to 4828.5) | 9937.7 (7889.4 to 12407.5) |  | 6103.4 (5491.8 to 6755.3) | 9932.8 (9116.5 to 10775.9) |
| American Samoa | 5.2 (4.2 to 6.2) | 5.1 (4 to 6.4) |  | 10.6 (9.5 to 11.8) | 8.5 (7.8 to 9.4) |
| Andorra | 4.6 (3.6 to 5.7) | 4.9 (3.8 to 6.2) |  | 7 (6.1 to 8) | 6.7 (5.9 to 7.6) |
| Angola | 760.6 (614.1 to 918.7) | 2274.2 (1770.2 to 2883.1) |  | 1858.3 (1658.4 to 2073.8) | 5146.9 (4664.3 to 5694.2) |
| Antigua and Barbuda | 10.6 (8.9 to 12.7) | 17.1 (13.7 to 21) |  | 9.4 (8.3 to 10.4) | 12 (10.8 to 13.3) |
| Argentina | 2172.5 (1757 to 2658.1) | 3080.3 (2400.2 to 3860.4) |  | 3846.9 (3386.1 to 4355.9) | 4844.2 (4322.5 to 5413) |
| Armenia | 568.7 (479.3 to 671) | 573.9 (458.8 to 701.5) |  | 747 (670.5 to 829.9) | 516.6 (470.2 to 570.7) |
| Australia | 1316.3 (1078.1 to 1584.8) | 1568.6 (1220.3 to 2000) |  | 1952.2 (1745.9 to 2167.5) | 2460.5 (2251.3 to 2680) |
| Austria | 541.1 (427.8 to 674.7) | 631.1 (497.2 to 792.9) |  | 1145.5 (1008.4 to 1294.6) | 931.3 (825.9 to 1050.4) |
| Azerbaijan | 1149.6 (969.1 to 1375.7) | 2041.2 (1614.4 to 2513.8) |  | 1339.2 (1188.3 to 1503.8) | 1798.2 (1624 to 1989.1) |
| Bahrain | 174.3 (147.2 to 206) | 490.2 (393.2 to 603.7) |  | 159.6 (143.5 to 177.9) | 356.2 (323.5 to 391.4) |
| Bangladesh | 10517.6 (8630.3 to 12653.8) | 22384.9 (17659.4 to 27571.6) |  | 11653.9 (10217.1 to 13230.8) | 20412.5 (18260 to 22635) |
| Barbados | 47.3 (39.6 to 55.9) | 51.5 (40.9 to 63.1) |  | 42.3 (37.1 to 47.9) | 37.5 (33.9 to 41.4) |
| Belarus | 2101.3 (1773.9 to 2471.9) | 1836.9 (1468.8 to 2254.7) |  | 1949.9 (1725 to 2200.2) | 1472.7 (1341.5 to 1620) |
| Belgium | 733 (600 to 887.8) | 642.9 (493.9 to 818.8) |  | 1188.7 (1005 to 1387) | 899.8 (787.8 to 1012.9) |
| Belize | 22.1 (18.5 to 26.4) | 74.2 (58.8 to 91.9) |  | 21.2 (18.7 to 23.8) | 56.4 (50.9 to 62.6) |
| Benin | 331.3 (268.9 to 401.7) | 1100.6 (848.6 to 1395.9) |  | 1034.4 (935.4 to 1143.5) | 2861.1 (2612.4 to 3134) |
| Bermuda | 13.2 (11.1 to 15.7) | 10.3 (8.3 to 12.5) |  | 9.8 (8.7 to 11) | 6.3 (5.7 to 7) |
| Bhutan | 65.9 (54.2 to 79.6) | 120.7 (95.6 to 150.4) |  | 71 (61.9 to 81.2) | 97.2 (86.4 to 108.5) |
| Bolivia | 597.2 (485.4 to 724.8) | 1380.2 (1079.6 to 1729.1) |  | 839.6 (749.2 to 937.1) | 1415.1 (1271.1 to 1575.9) |
| Bosnia and Herzegovina | 942.4 (792.1 to 1114.9) | 600.5 (480.1 to 731.2) |  | 1197.9 (1090.2 to 1317.1) | 710.3 (657.1 to 766.8) |
| Botswana | 96 (77.7 to 116.4) | 311.7 (239.9 to 387.8) |  | 225.7 (199 to 253.1) | 498.6 (453.6 to 548.6) |
| Brazil | 18079 (13618.9 to 23291.9) | 28443.5 (21097.2 to 37234) |  | 19958.3 (16105.7 to 24304) | 22229.9 (18383.1 to 26406.7) |
| Brunei | 18.5 (14.6 to 22.9) | 31.1 (23.9 to 39.4) |  | 46.7 (41.3 to 52.9) | 62.7 (55.9 to 69.7) |
| Bulgaria | 1429.7 (1200.2 to 1690.2) | 1143.8 (910.6 to 1402.1) |  | 1323 (1145.3 to 1518.4) | 926.9 (812.5 to 1049.1) |
| Burkina Faso | 555.5 (445.1 to 674.9) | 1665.2 (1286.5 to 2067.4) |  | 1593.1 (1423.1 to 1771) | 3903.4 (3552.9 to 4310.6) |
| Burundi | 373.3 (299.4 to 453.3) | 1030.2 (798.6 to 1295.2) |  | 1020.9 (918.2 to 1137.6) | 2119.9 (1920.6 to 2341.9) |
| Cabo Verde | 24.3 (19.6 to 29.3) | 76.6 (59.9 to 94.9) |  | 80.4 (72.3 to 89.5) | 158.7 (145.3 to 173) |
| Cambodia | 590 (473.1 to 724.7) | 1423.3 (1103.3 to 1794.6) |  | 1223.6 (1079.1 to 1378.2) | 2310 (2076.5 to 2561.7) |
| Cameroon | 598.6 (478.6 to 729.5) | 2518.5 (1955.2 to 3143.7) |  | 2064.8 (1857.4 to 2292.1) | 7145.6 (6533.4 to 7773.5) |
| Canada | 2654.1 (2150.4 to 3254.1) | 2484.8 (1946.5 to 3117.8) |  | 5302.9 (4768.9 to 5910.1) | 5176.4 (4736.3 to 5631.6) |
| Central African Republic | 173.2 (139.4 to 209.4) | 390 (296.8 to 498.1) |  | 452.5 (403.6 to 506.1) | 880.9 (797.2 to 973.7) |
| Chad | 429 (347.4 to 519.3) | 1283.9 (985.8 to 1624.1) |  | 1299.3 (1170.4 to 1441.3) | 3568.3 (3267.8 to 3876.7) |
| Chile | 798.2 (624 to 996.1) | 1230.3 (950.9 to 1555.9) |  | 1796.4 (1580.9 to 2035.5) | 2210.3 (1973.8 to 2453) |
| China | 185497.4 (137838.9 to 244821.7) | 223108.9 (160973.9 to 302389.1) |  | 225908.2 (185774.8 to 270081.2) | 195030.3 (161942 to 229580.4) |
| Colombia | 3910.7 (3234 to 4700.6) | 6184.4 (4901.6 to 7766.8) |  | 4918.6 (4369.6 to 5512.3) | 5569.3 (4992.8 to 6219.9) |
| Comoros | 32.5 (26 to 39.4) | 75.2 (57.9 to 93.4) |  | 82.6 (73.9 to 92.5) | 136.2 (123.3 to 150.3) |
| Cook Islands | 1.9 (1.5 to 2.2) | 1.7 (1.4 to 2.1) |  | 3.8 (3.5 to 4.3) | 3.1 (2.8 to 3.4) |
| Costa Rica | 461.1 (383.5 to 550.6) | 821.3 (652.7 to 1014.1) |  | 467.3 (411.9 to 527) | 642.3 (576.8 to 714) |
| Croatia | 922.7 (769.2 to 1090.5) | 665.9 (528.6 to 817.1) |  | 653.5 (551.6 to 762.8) | 387 (346.1 to 429.3) |
| Cuba | 1928.8 (1613.6 to 2306.1) | 1743.9 (1382.6 to 2162.4) |  | 1696.9 (1498.4 to 1909.2) | 1209.7 (1094.1 to 1336.9) |
| Cyprus | 48.2 (37.7 to 60.8) | 97.5 (75.5 to 122.7) |  | 71.9 (61.1 to 83.5) | 112.3 (96.2 to 129.4) |
| Czechia | 2424.5 (2061.8 to 2846) | 2004.5 (1607.4 to 2435.5) |  | 1573.6 (1375.8 to 1783.6) | 1052.3 (945.7 to 1168.8) |
| Democratic Republic of the Congo | 2543.5 (2050.1 to 3077.2) | 6468.1 (4953.8 to 8209.8) |  | 6071 (5386 to 6810.1) | 12969.8 (11598.8 to 14451.2) |
| Denmark | 306.7 (235.3 to 404.4) | 268.7 (207.2 to 343.4) |  | 587.8 (515.3 to 664.8) | 445.2 (392.6 to 501.7) |
| Djibouti | 33.9 (27.2 to 41.5) | 152 (117.5 to 191) |  | 80.1 (71.4 to 89.7) | 264.2 (240.3 to 291.1) |
| Dominica | 10.4 (8.6 to 12.5) | 11.5 (9.1 to 14.2) |  | 9.7 (8.6 to 10.9) | 8.2 (7.4 to 9.1) |
| Dominican Republic | 996.4 (825.8 to 1188.8) | 1944.1 (1546.9 to 2422.2) |  | 1084.6 (970.9 to 1209.8) | 1688.8 (1533.2 to 1854.4) |
| East Timor | 55.2 (44.6 to 67.8) | 94.1 (72.8 to 120.3) |  | 104.6 (92.3 to 118) | 177.7 (159.9 to 196.3) |
| Ecuador | 996.2 (814.4 to 1208.3) | 2198.7 (1726.6 to 2765.5) |  | 1535.6 (1370.7 to 1722.6) | 2495.5 (2258.6 to 2740.3) |
| Egypt | 8672.5 (7192.5 to 10402.9) | 22281.6 (17733.2 to 27943.2) |  | 12253 (10898.3 to 13761.3) | 25753.8 (23634.5 to 27930.5) |
| El Salvador | 485.4 (392.6 to 592.7) | 720.6 (565.2 to 908.5) |  | 653.2 (578.3 to 733.5) | 704 (627.7 to 784.5) |
| Equatorial Guinea | 27.3 (22.2 to 32.9) | 143 (110.7 to 180.6) |  | 69.4 (62.1 to 77.6) | 292.3 (263.2 to 323.9) |
| Eritrea | 213.8 (171.5 to 260.1) | 552.8 (426.5 to 692.9) |  | 527.6 (468.2 to 593.4) | 1111.8 (1004.5 to 1222.5) |
| Estonia | 351.7 (303.1 to 406.3) | 269 (215 to 329.3) |  | 234.2 (200.1 to 272.6) | 152.9 (137.4 to 169.7) |
| eSwatini | 51 (40.9 to 62.5) | 119.6 (92.8 to 151.7) |  | 114.9 (101.6 to 129.4) | 198.2 (179.3 to 219.2) |
| Ethiopia | 3329.6 (2440.8 to 4395.4) | 8557.7 (6027.4 to 11675.5) |  | 7080 (5695.5 to 8611.5) | 13927.3 (11508.1 to 16637.2) |
| Federated States of Micronesia | 9.4 (7.7 to 11.4) | 10.4 (8.1 to 13.1) |  | 22.7 (20.4 to 25) | 22.8 (20.9 to 24.8) |
| Fiji | 94.7 (78.2 to 113.8) | 121.5 (96.4 to 149.6) |  | 204.6 (184.4 to 226.2) | 218.7 (200.9 to 238.1) |
| Finland | 551 (440.3 to 680.9) | 340.5 (263.5 to 432.2) |  | 674 (605.4 to 750.2) | 571.2 (511.1 to 636.3) |
| France | 4478.5 (3670.4 to 5438.7) | 3465.6 (2676.5 to 4351.8) |  | 5310.9 (4598.6 to 6041.6) | 3902.9 (3520.2 to 4327.9) |
| Gabon | 70.2 (56.7 to 84.7) | 152.4 (117.4 to 193.3) |  | 179.7 (160.1 to 201.9) | 332.7 (301.1 to 367.4) |
| Gambia | 79.9 (64.5 to 97.3) | 239.7 (183.9 to 301.5) |  | 267.4 (242.5 to 294.1) | 622.8 (570.6 to 678.7) |
| Georgia | 947.7 (798.1 to 1124.1) | 609.8 (485.5 to 750.4) |  | 1320.7 (1179 to 1474.7) | 667 (606.9 to 732) |
| Germany | 7863.9 (6488.4 to 9473.1) | 5774 (4499.3 to 7198.2) |  | 12857 (11456.2 to 14383.2) | 9184 (8136.4 to 10282.3) |
| Ghana | 1168.5 (942.1 to 1411.5) | 3600.7 (2776.6 to 4498.3) |  | 4933.7 (4499.4 to 5388.9) | 12898.1 (11934.7 to 13891.9) |
| Greece | 610.5 (473.5 to 769.2) | 483.1 (373.6 to 614.2) |  | 1288.8 (1115.2 to 1479) | 800.9 (708.4 to 904.3) |
| Greenland | 5.2 (4.3 to 6.3) | 4.2 (3.3 to 5.3) |  | 15.9 (14.3 to 17.6) | 9.1 (8.2 to 10.1) |
| Grenada | 12.4 (10.4 to 14.8) | 17.5 (13.9 to 21.7) |  | 13.6 (11.8 to 15.6) | 14.7 (12.8 to 16.8) |
| Guam | 14.8 (12.2 to 17.9) | 16.2 (12.7 to 20.2) |  | 31.6 (28.4 to 35.1) | 32.9 (30.1 to 35.7) |
| Guatemala | 636 (490 to 791.6) | 1599.9 (1251.4 to 2027.7) |  | 864.4 (769.2 to 974.4) | 1806.5 (1614.2 to 2010.8) |
| Guinea | 387.7 (315.1 to 467.1) | 1047.3 (805.8 to 1317.7) |  | 1237.2 (1119.2 to 1371.3) | 3058.6 (2804.4 to 3326.2) |
| Guinea-Bissau | 68.8 (55.3 to 83.6) | 186.3 (144.5 to 233.3) |  | 270.9 (246.5 to 297.3) | 558.4 (514.1 to 604.7) |
| Guyana | 117.5 (97.3 to 139.5) | 118.6 (94 to 147.3) |  | 159.4 (143 to 177.9) | 119.8 (109.2 to 131.2) |
| Haiti | 864.1 (716.4 to 1037.5) | 2339.6 (1839.1 to 2892.2) |  | 887.4 (799.7 to 983.2) | 1878.1 (1713.3 to 2053.6) |
| Honduras | 455 (375.5 to 542.8) | 1287.7 (1015.2 to 1598.3) |  | 594.3 (526.9 to 666.8) | 1283.2 (1142.5 to 1434.5) |
| Hungary | 2721.8 (2314.9 to 3192.1) | 1614.2 (1290.4 to 1972.6) |  | 1932.6 (1745.7 to 2144.6) | 1098.3 (989.3 to 1215) |
| Iceland | 20.4 (16.3 to 25.3) | 22.1 (17.1 to 28) |  | 33.9 (29.6 to 38.8) | 33.8 (30 to 38.2) |
| India | 105253 (78119.9 to 139641.9) | 228859.1 (163235.9 to 309504.1) |  | 100590.7 (80526.6 to 122272.2) | 178653.5 (147156.9 to 213584.5) |
| Indonesia | 16448.1 (12281.4 to 21455.7) | 30137.2 (21962.6 to 40289.2) |  | 42273.8 (34440.7 to 51151.4) | 54421 (45105.9 to 64675.8) |
| Iran | 8990.4 (6711.6 to 11867.5) | 24078 (17213.3 to 32803) |  | 16102.2 (13238.2 to 19221.7) | 22365.5 (18835 to 26216.2) |
| Iraq | 3125.8 (2622.8 to 3726.9) | 10088.7 (8029.1 to 12407.2) |  | 4875.7 (4428.8 to 5369.2) | 11282.8 (10326.4 to 12270.1) |
| Ireland | 246.9 (198 to 306.5) | 277.9 (215 to 350.7) |  | 394.7 (337.9 to 454.3) | 354.9 (309.9 to 403.8) |
| Israel | 361.3 (283.4 to 455.5) | 580.2 (444.1 to 731.6) |  | 555.5 (486.9 to 628.8) | 825 (726.2 to 927.9) |
| Italy | 4743.8 (3637.4 to 6063.8) | 3464.6 (2550.7 to 4600.1) |  | 7251.2 (5833.5 to 8892.1) | 4167.8 (3437.4 to 4952) |
| Ivory Coast | 966.1 (780.5 to 1168.6) | 2841.4 (2205 to 3580.8) |  | 3547.7 (3228 to 3871.5) | 7471.1 (6874.5 to 8122.9) |
| Jamaica | 348.8 (287.6 to 419.6) | 563.3 (447 to 692.1) |  | 333.2 (294.2 to 375.9) | 384.2 (346.3 to 425.4) |
| Japan | 7209.5 (5393.6 to 9378.3) | 4896.1 (3604.5 to 6417.4) |  | 13551.5 (10871.1 to 16535) | 10176.6 (8421.8 to 12065.4) |
| Jordan | 641.6 (533.7 to 773.1) | 3127.1 (2490.7 to 3886.5) |  | 1050 (938.7 to 1163.3) | 3395.2 (3076.6 to 3718.6) |
| Kazakhstan | 3039.2 (2555.4 to 3601.5) | 3712.4 (2949.8 to 4553.4) |  | 5344.1 (4872 to 5852.1) | 4387.1 (4006.7 to 4795.6) |
| Kenya | 1789.9 (1325.6 to 2341.6) | 5263.1 (3834.6 to 7016.3) |  | 4109.9 (3328.8 to 4989.9) | 9113.6 (7558.5 to 10833.7) |
| Kiribati | 8 (6.5 to 9.6) | 14.1 (11.1 to 17.6) |  | 23.6 (21.7 to 25.8) | 35.7 (33 to 38.3) |
| Kuwait | 606.4 (510 to 718.9) | 1938.8 (1573 to 2377.4) |  | 598.6 (541.5 to 659.5) | 1489.9 (1363.6 to 1628) |
| Kyrgyzstan | 630.3 (527.2 to 754.4) | 907.9 (704.5 to 1133.8) |  | 978 (879.2 to 1084.8) | 1077 (978.1 to 1188.9) |
| Laos | 273.8 (222.9 to 332.8) | 644.8 (494.9 to 817.2) |  | 593.2 (526 to 668.2) | 1229.7 (1114.1 to 1355) |
| Latvia | 414.4 (347.4 to 493.2) | 269 (213 to 334.4) |  | 417.1 (354.3 to 482.9) | 211.3 (189.7 to 234.6) |
| Lebanon | 501.9 (420.6 to 602.4) | 1572.7 (1257.4 to 1932.3) |  | 688.8 (621.8 to 761.5) | 1659 (1521.1 to 1799.9) |
| Lesotho | 84.1 (67.5 to 102.4) | 157.5 (121.6 to 200.4) |  | 186.2 (163.8 to 210.6) | 275 (246.9 to 304.6) |
| Liberia | 201.3 (163.2 to 244.2) | 551.8 (426.8 to 694.6) |  | 600.2 (542.8 to 665.2) | 1257.8 (1148.3 to 1373.1) |
| Libya | 700 (576.6 to 845) | 1891.8 (1505.3 to 2365) |  | 1132.9 (1029.6 to 1240.6) | 2264.8 (2100.5 to 2432.9) |
| Lithuania | 576.1 (487.5 to 681.5) | 349.6 (275.8 to 433) |  | 710.5 (565.9 to 875.4) | 374.1 (314.1 to 440.6) |
| Luxembourg | 25.8 (20.1 to 32.3) | 40.1 (31 to 50.6) |  | 48 (39.1 to 58.4) | 54.9 (48.5 to 62.1) |
| Madagascar | 839.6 (675.3 to 1024.2) | 2436.4 (1861.6 to 3063.8) |  | 2445.9 (2201.3 to 2709.5) | 5985.3 (5448 to 6575.8) |
| Malawi | 699 (562.9 to 850.9) | 1723.5 (1331.6 to 2165.8) |  | 1752.6 (1566.1 to 1965.5) | 3590.5 (3255.7 to 3971.1) |
| Malaysia | 1754.2 (1432.8 to 2122.3) | 4439.4 (3499.4 to 5553.2) |  | 3084.7 (2742.8 to 3473.1) | 6323.7 (5735.2 to 6951.9) |
| Maldives | 15.2 (12.4 to 18.6) | 86.2 (68.6 to 107.9) |  | 34.1 (30.4 to 38.3) | 97.7 (88.6 to 107.4) |
| Mali | 538.4 (435.5 to 647.8) | 1603.1 (1227.9 to 2046.8) |  | 1711.3 (1531.9 to 1907.5) | 4503 (4099.1 to 4935.4) |
| Malta | 26.3 (20.6 to 33.1) | 25.8 (19.9 to 32.8) |  | 41.5 (35.3 to 48.2) | 33.7 (29.7 to 38.2) |
| Marshall Islands | 3.7 (3 to 4.5) | 6 (4.7 to 7.5) |  | 9.1 (8.2 to 10.1) | 12.8 (11.8 to 14) |
| Mauritania | 178 (145.4 to 214.7) | 421.8 (327.8 to 527) |  | 561.9 (509.3 to 620.4) | 989.1 (899.6 to 1088.1) |
| Mauritius | 122.7 (99.7 to 149.6) | 119.8 (93.9 to 150) |  | 245.2 (220.5 to 272.9) | 169.3 (153.3 to 187.6) |
| Mexico | 11019.7 (8270 to 14430.6) | 19873.2 (14362.2 to 26843.4) |  | 16143.3 (13080.3 to 19585.9) | 19296.2 (15962.8 to 22801.7) |
| Moldova | 809.7 (679.6 to 963.4) | 722.8 (569 to 888.1) |  | 724.3 (641.9 to 817.7) | 551.4 (502.3 to 605.4) |
| Monaco | 1.7 (1.3 to 2.1) | 1.7 (1.3 to 2.1) |  | 3.1 (2.7 to 3.5) | 2.8 (2.5 to 3.2) |
| Mongolia | 306.7 (253 to 366.7) | 619.5 (488.9 to 764.6) |  | 507.7 (459.7 to 556.1) | 759 (693.4 to 824.6) |
| Montenegro | 145.6 (123 to 170.9) | 134.6 (107.2 to 163.8) |  | 85.8 (75.8 to 96.4) | 72.8 (65.4 to 80.8) |
| Morocco | 4850.3 (4006 to 5849.4) | 8538.5 (6789.9 to 10538.1) |  | 6442.4 (5820.3 to 7136.7) | 8857.3 (8095.9 to 9664.4) |
| Mozambique | 932.7 (748.5 to 1148.6) | 2410.2 (1845.9 to 3028.6) |  | 2225.9 (1981 to 2499.3) | 5601.6 (5076.6 to 6163.6) |
| Myanmar | 3009.3 (2451 to 3643.7) | 4237.1 (3280.8 to 5385.5) |  | 7008.9 (6230 to 7830.2) | 8694.3 (7905.6 to 9522.9) |
| Namibia | 110.8 (90.2 to 134.6) | 236.6 (183.3 to 297) |  | 235.5 (207.2 to 265.8) | 395.8 (355.5 to 438.9) |
| Nauru | 1.1 (0.9 to 1.3) | 1.3 (1 to 1.6) |  | 3.5 (3.2 to 3.8) | 4.1 (3.8 to 4.4) |
| Nepal | 1944.6 (1610.9 to 2333) | 3620.3 (2842.8 to 4566.5) |  | 1940.7 (1701.3 to 2210.1) | 3278.3 (2898.9 to 3669.7) |
| Netherlands | 1380.7 (1114.2 to 1697.6) | 980.4 (750.7 to 1228.4) |  | 2361 (2074.5 to 2664.1) | 1602.2 (1413.1 to 1800) |
| New Zealand | 383.4 (284.4 to 507.1) | 366.7 (260 to 499.8) |  | 460.3 (369.3 to 563) | 567 (467.2 to 672.3) |
| Nicaragua | 385.5 (318.8 to 463.6) | 888.7 (698.8 to 1116.4) |  | 477.2 (421.1 to 542.6) | 819.1 (730.1 to 911.6) |
| Niger | 499 (399.4 to 604) | 1570.7 (1212.3 to 1994.3) |  | 1629.4 (1471.2 to 1803.8) | 4399.2 (3981 to 4851) |
| Nigeria | 7332.2 (5382.2 to 9652.4) | 21606.3 (15460.2 to 29186.8) |  | 19747 (16101.1 to 23737.1) | 49832.4 (41420.5 to 58743.9) |
| Niue | 0.2 (0.2 to 0.3) | 0.2 (0.1 to 0.2) |  | 0.5 (0.5 to 0.6) | 0.4 (0.3 to 0.4) |
| North Korea | 2518.5 (2089 to 3018.7) | 3710.1 (2921 to 4607.2) |  | 4167 (3746.8 to 4613) | 4858.8 (4435.6 to 5311.9) |
| North Macedonia | 428.5 (359 to 508) | 482.7 (384 to 592.1) |  | 414.7 (353.1 to 482.6) | 366.3 (311.9 to 424.6) |
| Northern Mariana Islands | 7.4 (6.1 to 8.9) | 5.5 (4.3 to 6.8) |  | 11.8 (10.5 to 13.2) | 7.8 (7.1 to 8.6) |
| Norway | 346.9 (264.6 to 447.9) | 340 (247.1 to 452.9) |  | 644.1 (520.3 to 792.9) | 530.8 (438.2 to 630) |
| Oman | 389.5 (327 to 462.8) | 1756.7 (1417.2 to 2159.8) |  | 566.9 (515.7 to 620.8) | 2010.5 (1861.2 to 2156.6) |
| Pakistan | 13729.4 (10280.9 to 17965.9) | 40435.2 (29199.4 to 54621.2) |  | 15174.6 (12258.8 to 18457.9) | 39161.4 (32394.2 to 47107.7) |
| Palau | 1.7 (1.3 to 2) | 2 (1.6 to 2.4) |  | 4.3 (3.9 to 4.8) | 4 (3.7 to 4.3) |
| Palestine | 301.9 (250.8 to 363.7) | 1023.3 (809.6 to 1275.1) |  | 406.8 (363 to 454.3) | 1069.9 (967.4 to 1178.2) |
| Panama | 274.7 (228.6 to 329.3) | 542.4 (430.1 to 676.6) |  | 353 (314.6 to 395.1) | 486.5 (435.5 to 541) |
| Papua New Guinea | 355.9 (291 to 432.4) | 1049.1 (811.4 to 1326.5) |  | 637.6 (565.1 to 714.7) | 1556.6 (1402.4 to 1713.9) |
| Paraguay | 438.4 (357.2 to 531) | 1002 (793.4 to 1243.9) |  | 402.7 (353.8 to 457.7) | 727.7 (647.3 to 815.9) |
| Peru | 2093.2 (1698.8 to 2545.5) | 4691.9 (3689.1 to 5899.3) |  | 2888 (2574.7 to 3237.2) | 4751.9 (4279.2 to 5255.6) |
| Philippines | 5262.8 (3974 to 6824.4) | 11028.8 (8137.3 to 14634.7) |  | 9339.4 (7538.3 to 11272.1) | 18771.8 (15552.3 to 22270.6) |
| Poland | 8181.7 (6256.9 to 10540.1) | 6645.3 (4942.1 to 8752.5) |  | 5388.2 (4376.4 to 6501.1) | 3854.3 (3201.9 to 4571.4) |
| Portugal | 484.5 (375.8 to 613.1) | 457.8 (345.8 to 591.2) |  | 1111.1 (920.1 to 1324.6) | 650 (567.7 to 742.5) |
| Puerto Rico | 625 (527.4 to 744.3) | 528.4 (424.4 to 650) |  | 474.1 (419.3 to 531.5) | 343 (307.5 to 381.5) |
| Qatar | 180.3 (153.2 to 212.6) | 1421.3 (1146.6 to 1743.4) |  | 179.4 (163.3 to 196.6) | 1084.3 (983.3 to 1189.1) |
| Republic of Serbia | 2586.4 (2199.2 to 3049.3) | 2315 (1859 to 2804.1) |  | 1684.3 (1435.8 to 1964.8) | 1157.5 (988 to 1342.6) |
| Republic of the Congo | 167 (134.6 to 202) | 495.8 (382.9 to 626.7) |  | 470.4 (417.7 to 525.5) | 1038.4 (940.7 to 1143.3) |
| Romania | 4603.1 (3877.6 to 5414.1) | 3143.4 (2503.4 to 3833.6) |  | 3733.3 (3227 to 4269.4) | 2047.8 (1837.8 to 2273.2) |
| Russia | 45371.3 (33948.7 to 59417.7) | 44836.1 (32431.7 to 60311.5) |  | 31244.2 (25575.3 to 37687) | 23033.1 (19303.3 to 27219.9) |
| Rwanda | 473.4 (381.3 to 578.9) | 1083.6 (832.8 to 1371) |  | 1319.7 (1179.2 to 1471.3) | 2164.5 (1953.5 to 2392.4) |
| Saint Kitts and Nevis | 6.8 (5.6 to 8.1) | 11.3 (9 to 13.9) |  | 8.7 (7.5 to 9.9) | 8.6 (7.8 to 9.5) |
| Saint Lucia | 21.2 (17.7 to 25.3) | 33 (26.3 to 40.6) |  | 23.1 (19.8 to 26.5) | 24.2 (21.9 to 26.8) |
| Saint Vincent and the Grenadines | 15 (12.4 to 18) | 17.6 (14 to 21.8) |  | 19.2 (16.9 to 21.6) | 13.8 (12.5 to 15.2) |
| Samoa | 15.7 (12.8 to 18.9) | 22.4 (17.7 to 28.1) |  | 36.1 (32.4 to 40.2) | 45.5 (41.6 to 49.5) |
| San Marino | 1.5 (1.2 to 1.9) | 1.5 (1.2 to 1.9) |  | 2.7 (2.3 to 3) | 2.4 (2.2 to 2.8) |
| São Tomé and Principe | 8.2 (6.6 to 10) | 23.5 (18.3 to 29.4) |  | 31.5 (28.5 to 34.6) | 71.9 (66.2 to 77.7) |
| Saudi Arabia | 2852.5 (2402.5 to 3415.3) | 14649.9 (11740.4 to 17880.8) |  | 3317.1 (2973.1 to 3688.2) | 10692.7 (9835.9 to 11618.6) |
| Senegal | 632.9 (511.9 to 767.1) | 1636.6 (1271 to 2067.9) |  | 2035.9 (1838.2 to 2251.1) | 4156.5 (3799.3 to 4551.9) |
| Seychelles | 6.2 (4.9 to 7.5) | 10.4 (8.2 to 13.1) |  | 13 (11.6 to 14.5) | 16.4 (15 to 18) |
| Sierra Leone | 377.5 (307.5 to 453) | 843.6 (660.9 to 1063.9) |  | 1197.9 (1089.8 to 1320.4) | 2459 (2266.8 to 2663) |
| Singapore | 301.9 (240.4 to 370.6) | 397.6 (325.2 to 481.8) |  | 520.7 (458.7 to 588.2) | 570.2 (508.8 to 638.3) |
| Slovakia | 1106.3 (930.6 to 1303.7) | 954.8 (762.7 to 1168.6) |  | 906.7 (810.7 to 1015.8) | 728.2 (660.1 to 803.6) |
| Slovenia | 455.1 (383.2 to 539.2) | 351.3 (279.9 to 430.6) |  | 260.1 (217.8 to 307.4) | 152.6 (135.4 to 170.6) |
| Solomon Islands | 25.8 (21.1 to 31.2) | 67.5 (52.6 to 85.3) |  | 70.2 (63.3 to 77.5) | 152.7 (139.8 to 166.1) |
| Somalia | 581.1 (468.2 to 707.2) | 1628.8 (1247.3 to 2048.4) |  | 1332.6 (1192.6 to 1492.5) | 3449.4 (3118.1 to 3818.2) |
| South Africa | 4223.6 (3118.1 to 5563.1) | 8003.4 (5718.3 to 10789.2) |  | 9577.5 (7754.5 to 11561) | 11763.9 (9743.5 to 13874.3) |
| South Korea | 2520.7 (1962.4 to 3158.9) | 2336.4 (1793 to 2967.7) |  | 9128.3 (8117.2 to 10185.4) | 5702.1 (5115.5 to 6321.6) |
| South Sudan | 387.2 (309 to 470.1) | 683.8 (524.9 to 859.2) |  | 932.9 (826.4 to 1056.9) | 1396.8 (1256.9 to 1548.7) |
| Spain | 2420.1 (1946.7 to 2983.9) | 2204.8 (1697.7 to 2764) |  | 4151.6 (3538.6 to 4804.3) | 2848.5 (2551.7 to 3179.4) |
| Sri Lanka | 1532.9 (1244.1 to 1873.6) | 1931.8 (1513.4 to 2434.2) |  | 3243.6 (2893.7 to 3632) | 3156.8 (2846.7 to 3497.5) |
| Sudan | 3127.3 (2595.7 to 3749) | 9222.8 (7288.2 to 11582.8) |  | 4507 (4059.9 to 4997.5) | 11195 (10281.9 to 12154.1) |
| Suriname | 60 (49.9 to 72.3) | 104.5 (83.3 to 128.2) |  | 63.2 (56.8 to 70.3) | 78.3 (71.3 to 85.9) |
| Sweden | 495 (371.7 to 641.1) | 574.5 (422.6 to 761) |  | 988.4 (795.5 to 1212.6) | 1463.4 (1206.4 to 1744.6) |
| Switzerland | 482.5 (388.2 to 600.9) | 470.6 (359.3 to 598.1) |  | 723.4 (622.6 to 832.4) | 600.6 (528.9 to 682.9) |
| Syria | 1933.5 (1575.3 to 2345.2) | 2384.9 (1880.9 to 3025.7) |  | 4057.4 (3726.7 to 4428.7) | 3232.2 (2961.9 to 3513.8) |
| Tajikistan | 719.1 (594.8 to 866.5) | 1589.2 (1235.1 to 1982.2) |  | 862.1 (764.8 to 969.4) | 1641.9 (1484.6 to 1818.3) |
| Thailand | 4620.8 (3714.8 to 5627.9) | 4737 (3731.4 to 5926) |  | 9552.2 (8498 to 10743.2) | 7849 (7111.8 to 8667.5) |
| The Bahamas | 46.5 (38.7 to 55.7) | 74.4 (59 to 92.5) |  | 43.6 (38.8 to 48.6) | 56.1 (50.7 to 61.8) |
| Togo | 266.7 (214.8 to 323.9) | 774.9 (599.2 to 978.7) |  | 942.9 (855.2 to 1040.8) | 2069 (1904.5 to 2237.2) |
| Tokelau | 0.1 (0.1 to 0.1) | 0.1 (0.1 to 0.2) |  | 0.3 (0.2 to 0.3) | 0.2 (0.2 to 0.3) |
| Tonga | 8.5 (6.9 to 10.3) | 10.9 (8.6 to 13.7) |  | 17.3 (15.4 to 19.3) | 18.3 (16.6 to 20.1) |
| Trinidad and Tobago | 246.2 (207.2 to 292.2) | 333.8 (266.4 to 406.5) |  | 262.6 (237 to 291) | 228.5 (208.9 to 250.3) |
| Tunisia | 1359 (1125 to 1631.3) | 2487 (1990.4 to 3048.9) |  | 1565.8 (1397.8 to 1754.2) | 2237.1 (2046 to 2455.4) |
| Turkey | 10988.6 (9244.6 to 13075.9) | 16082.4 (12787.9 to 19980.3) |  | 16437.5 (14715.8 to 18247) | 17200.2 (15576.7 to 18933.1) |
| Turkmenistan | 512 (427.7 to 610.5) | 927.6 (732.4 to 1149.5) |  | 813.9 (734.7 to 898.3) | 1436.5 (1327.6 to 1547) |
| Tuvalu | 0.9 (0.7 to 1) | 1.3 (1 to 1.6) |  | 1.9 (1.7 to 2.1) | 2.5 (2.3 to 2.7) |
| Uganda | 1025.7 (816.7 to 1249.6) | 2855.5 (2178.3 to 3637.1) |  | 3059.7 (2728 to 3439.2) | 7144.9 (6461.3 to 7907.5) |
| Ukraine | 10294.5 (7724.6 to 13461) | 8911.1 (6402.5 to 11982.8) |  | 10780.7 (8766.4 to 13040.6) | 8334.9 (6966.6 to 9908.7) |
| United Arab Emirates | 698.1 (589.2 to 824.3) | 4905.1 (4010.5 to 5955.3) |  | 861 (781.5 to 945.4) | 3422.1 (3155.9 to 3691.4) |
| United Kingdom | 5043.2 (3864.2 to 6410.8) | 4493.1 (3345.6 to 5840) |  | 7488.1 (6201.2 to 8908) | 5964.6 (5028.7 to 6985.3) |
| United Republic of Tanzania | 1531.7 (1229 to 1860.4) | 4981.5 (3819.7 to 6294) |  | 4144.5 (3673.4 to 4671.2) | 11717.7 (10680.3 to 12871.8) |
| United States of America | 29396.1 (21653.8 to 39181.3) | 28329.3 (20463.5 to 38156.8) |  | 65039.6 (53058.5 to 78348.3) | 65923.2 (54823.6 to 77746) |
| United States Virgin Islands | 16.4 (13.7 to 19.4) | 11.7 (9.4 to 14.3) |  | 13.1 (11.5 to 14.8) | 7.5 (6.8 to 8.4) |
| Uruguay | 177.6 (140.4 to 220.8) | 188.3 (146.2 to 237.7) |  | 452.8 (404.1 to 504.2) | 340.8 (304.4 to 379.2) |
| Uzbekistan | 2798.2 (2280.3 to 3401.7) | 6160.5 (4867.5 to 7597.3) |  | 4004.8 (3547.5 to 4496.2) | 6897 (6264.3 to 7569.3) |
| Vanuatu | 15.6 (12.8 to 18.8) | 37.2 (29.2 to 46.6) |  | 39.8 (35.9 to 43.9) | 87.9 (80.9 to 95.4) |
| Venezuela | 2624.4 (2186.2 to 3123.6) | 3655.1 (2902.7 to 4509.3) |  | 2729.6 (2405.5 to 3081) | 3018.5 (2709.3 to 3340.3) |
| Vietnam | 5021.6 (4053.2 to 6137.4) | 9655.7 (7540.1 to 12090.9) |  | 9852.1 (8659.1 to 11153.6) | 14716.1 (13295.2 to 16226.3) |
| Yemen | 1839.9 (1535 to 2212.8) | 6226.7 (4943.4 to 7755.8) |  | 2297.3 (2058.5 to 2556.1) | 6488.3 (5922.7 to 7059.9) |
| Zambia | 446.1 (355.9 to 546.2) | 1434.2 (1090.7 to 1811.2) |  | 1202.8 (1067.6 to 1347.2) | 3115.7 (2808.9 to 3451.7) |
| Zimbabwe | 733.3 (585.6 to 900.6) | 1475.3 (1150.3 to 1846.4) |  | 1563.1 (1380.8 to 1754.3) | 2715.2 (2447.1 to 2988.9) |

| **Supplementary Table 9. Age standardized mortality number of ischemic heart disease vs ischemic stroke in youths and young Adults (15-39 years) at country level, 1990-2021, both sexes** | | | | | |
| --- | --- | --- | --- | --- | --- |
|  | **Ischemic heart disease (Mortality number, 95% UI)** | |  | **Ischemic stroke (Mortality number, 95% UI)** | |
|  | **Age standardized number in 1990** | **Age standardized number in 2021** |  | **Age standardized number in 1990** | **Age standardized number in 2021** |
| Afghanistan | 91.3 (57.2 to 136.3) | 303.1 (198.8 to 454.9) |  | 11 (5.8 to 19.1) | 47.3 (26.1 to 83.2) |
| Albania | 11.8 (9.1 to 15) | 6.1 (4.5 to 8.1) |  | 0.5 (0.3 to 0.7) | 0.2 (0.1 to 0.3) |
| Algeria | 239.8 (172 to 326) | 244.9 (169.9 to 336.8) |  | 26.8 (15.8 to 41.6) | 35.3 (20.3 to 56.7) |
| American Samoa | 0.4 (0.3 to 0.5) | 0.4 (0.3 to 0.6) |  | 0 (0 to 0) | 0 (0 to 0) |
| Andorra | 0.1 (0.1 to 0.2) | 0 (0 to 0.1) |  | 0 (0 to 0) | 0 (0 to 0) |
| Angola | 21.9 (13.6 to 32.5) | 68.9 (43.4 to 104) |  | 3 (1.7 to 4.9) | 8 (4.6 to 12.9) |
| Antigua and Barbuda | 0.2 (0.2 to 0.2) | 0.1 (0.1 to 0.1) |  | 0 (0 to 0) | 0 (0 to 0) |
| Argentina | 140.4 (122.6 to 159.6) | 71.4 (60.2 to 83) |  | 9.1 (7.4 to 11) | 3.4 (2.8 to 4.1) |
| Armenia | 26.2 (22.8 to 29.5) | 12.5 (10.8 to 14.4) |  | 2 (1.4 to 2.6) | 0.7 (0.6 to 0.8) |
| Australia | 39.8 (34.4 to 45.7) | 20.8 (17.5 to 24.5) |  | 2.4 (2 to 2.9) | 0.8 (0.6 to 0.9) |
| Austria | 18.9 (16.5 to 21.7) | 5.4 (4.6 to 6.3) |  | 2.4 (2 to 2.8) | 0.3 (0.2 to 0.3) |
| Azerbaijan | 85.8 (71.1 to 101.3) | 65.7 (51.1 to 82) |  | 3.5 (2.2 to 5) | 2 (1.1 to 3.3) |
| Bahrain | 6.1 (4.9 to 7.5) | 8.9 (6.7 to 11.3) |  | 0.3 (0.2 to 0.4) | 0.7 (0.5 to 1) |
| Bangladesh | 619.5 (440.8 to 841.7) | 843.4 (580.8 to 1167.2) |  | 56.5 (32.1 to 95.5) | 77.5 (39.7 to 146) |
| Barbados | 0.8 (0.6 to 0.9) | 0.3 (0.2 to 0.4) |  | 0.1 (0.1 to 0.2) | 0.1 (0 to 0.1) |
| Belarus | 84.8 (72 to 99.1) | 58.3 (46.2 to 71.9) |  | 6.2 (4.6 to 7.7) | 2.7 (2 to 3.5) |
| Belgium | 27.6 (24 to 31.3) | 5.5 (4.6 to 6.4) |  | 3.2 (2.7 to 3.8) | 0.3 (0.3 to 0.4) |
| Belize | 0.5 (0.4 to 0.6) | 0.8 (0.6 to 0.9) |  | 0 (0 to 0.1) | 0.1 (0.1 to 0.1) |
| Benin | 5.5 (3.6 to 8.1) | 19 (12 to 28.1) |  | 1.4 (0.8 to 2.2) | 4.5 (2.6 to 7.2) |
| Bermuda | 0.3 (0.2 to 0.3) | 0.1 (0 to 0.1) |  | 0 (0 to 0) | 0 (0 to 0) |
| Bhutan | 3.3 (1.8 to 5) | 4.1 (2.4 to 6.3) |  | 0.1 (0.1 to 0.2) | 0.1 (0.1 to 0.3) |
| Bolivia | 37.3 (25.4 to 54.6) | 34.2 (21.6 to 52.9) |  | 4.1 (2.2 to 6.7) | 3.1 (1.6 to 4.9) |
| Bosnia and Herzegovina | 23.9 (19 to 29.5) | 5.9 (3.9 to 8.1) |  | 2.8 (1.7 to 4.3) | 0.9 (0.6 to 1.3) |
| Botswana | 2.3 (1.1 to 3.8) | 4.6 (2.3 to 7.3) |  | 0.4 (0.2 to 0.7) | 0.6 (0.3 to 0.9) |
| Brazil | 737.7 (697 to 780.1) | 722.6 (678.4 to 769.3) |  | 95.4 (89.6 to 101.2) | 55.6 (52 to 59.8) |
| Brunei | 2.7 (2 to 3.5) | 2.9 (2.1 to 3.6) |  | 0.2 (0.1 to 0.3) | 0.1 (0.1 to 0.1) |
| Bulgaria | 74.2 (66.4 to 82.5) | 37.7 (31.2 to 44.8) |  | 6.8 (6 to 7.7) | 3.9 (3.2 to 4.7) |
| Burkina Faso | 9.1 (5.8 to 13.8) | 28.8 (17.4 to 44.5) |  | 1.8 (1 to 3.1) | 5.3 (3 to 9.2) |
| Burundi | 21 (13.3 to 31.9) | 46.6 (30.6 to 66.5) |  | 3.3 (1.8 to 5.6) | 4.1 (2.3 to 6.9) |
| Cabo Verde | 0.8 (0.6 to 1.1) | 1.5 (0.9 to 2.2) |  | 0.2 (0.1 to 0.4) | 0.3 (0.2 to 0.5) |
| Cambodia | 50.5 (35.7 to 69.5) | 77.4 (49.4 to 119.4) |  | 4.3 (2.5 to 6.9) | 5.3 (3.3 to 8.4) |
| Cameroon | 13.5 (8.4 to 19.9) | 73 (43.4 to 113.8) |  | 3.6 (2 to 6) | 16.3 (9 to 27.7) |
| Canada | 63 (54.6 to 71.9) | 33.2 (28 to 39) |  | 4 (3.4 to 4.8) | 2.2 (1.8 to 2.7) |
| Central African Republic | 7.7 (4.6 to 12.5) | 17.6 (9.9 to 28.8) |  | 0.9 (0.5 to 1.5) | 1.7 (0.9 to 3) |
| Chad | 7.8 (4.9 to 11.8) | 30.9 (19 to 45.9) |  | 2.2 (1.2 to 4.2) | 7.7 (4.3 to 13.6) |
| Chile | 25 (21.3 to 29.1) | 25 (20.9 to 29.2) |  | 3.8 (3.1 to 4.5) | 1.9 (1.6 to 2.2) |
| China | 5590.1 (4838.2 to 6410.3) | 5009 (4136.6 to 6013.2) |  | 669.9 (545.4 to 847.2) | 578.2 (464.8 to 698.5) |
| Colombia | 146.8 (128.3 to 167.5) | 106.5 (86.3 to 128.7) |  | 16.5 (14 to 19.5) | 7.9 (6.2 to 10) |
| Comoros | 1.2 (0.5 to 1.9) | 2.3 (1.5 to 3.3) |  | 0.2 (0.1 to 0.4) | 0.2 (0.1 to 0.4) |
| Cook Islands | 0.1 (0.1 to 0.2) | 0.1 (0.1 to 0.1) |  | 0 (0 to 0) | 0 (0 to 0) |
| Costa Rica | 9.1 (7.9 to 10.4) | 12.7 (10.7 to 14.9) |  | 0.7 (0.6 to 0.9) | 0.6 (0.5 to 0.7) |
| Croatia | 24 (21.3 to 27) | 4.6 (3.8 to 5.4) |  | 1.9 (1.6 to 2.2) | 0.3 (0.2 to 0.3) |
| Cuba | 55.2 (48.8 to 62.4) | 20.4 (16.7 to 24.3) |  | 4.7 (3.8 to 5.8) | 1.8 (1.5 to 2.3) |
| Cyprus | 1.8 (1.3 to 2.3) | 1.5 (1.1 to 2.1) |  | 0.1 (0.1 to 0.2) | 0.1 (0 to 0.1) |
| Czechia | 52 (46.7 to 57.8) | 12.3 (10.5 to 14.3) |  | 4.7 (3.9 to 5.6) | 0.8 (0.7 to 1) |
| Democratic Republic of the Congo | 76.5 (45.9 to 120) | 174.6 (107.3 to 270) |  | 9.8 (5.3 to 16.8) | 17.5 (9.2 to 30.5) |
| Denmark | 13.9 (12.2 to 15.9) | 3 (2.5 to 3.4) |  | 1.3 (1.1 to 1.5) | 0.1 (0.1 to 0.2) |
| Djibouti | 0.9 (0.5 to 1.4) | 4.4 (2.4 to 6.9) |  | 0.1 (0.1 to 0.2) | 0.4 (0.2 to 0.6) |
| Dominica | 0.1 (0.1 to 0.2) | 0.1 (0.1 to 0.1) |  | 0 (0 to 0) | 0 (0 to 0) |
| Dominican Republic | 50.1 (39 to 62.9) | 84.1 (60 to 114.8) |  | 3.6 (2.4 to 5.1) | 5.8 (3.7 to 8.8) |
| East Timor | 4.5 (2.9 to 6.5) | 8.7 (5.4 to 13.1) |  | 0.3 (0.2 to 0.5) | 0.6 (0.3 to 1.1) |
| Ecuador | 49.1 (42.5 to 56) | 68.4 (53.4 to 86.1) |  | 7.6 (6 to 9.2) | 3.8 (2.9 to 5) |
| Egypt | 1225.7 (1003.6 to 1475.1) | 1780.6 (1412.8 to 2196.3) |  | 101.6 (48.6 to 181) | 164.9 (99.9 to 256.7) |
| El Salvador | 33 (26.4 to 41.7) | 27.1 (19.4 to 37) |  | 2.6 (1.8 to 3.6) | 1.5 (1 to 2.3) |
| Equatorial Guinea | 1.1 (0.6 to 1.7) | 4.4 (2.3 to 7.3) |  | 0.1 (0.1 to 0.2) | 0.4 (0.2 to 0.8) |
| Eritrea | 9.3 (5.7 to 14.3) | 28.2 (16.7 to 43.2) |  | 1.1 (0.6 to 2) | 2.1 (1.2 to 3.6) |
| Estonia | 10.5 (8.8 to 12.6) | 1.1 (0.9 to 1.4) |  | 0.7 (0.6 to 0.9) | 0.1 (0.1 to 0.1) |
| eSwatini | 0.9 (0.5 to 1.5) | 3.6 (2 to 5.9) |  | 0.1 (0.1 to 0.2) | 0.4 (0.2 to 0.7) |
| Ethiopia | 174.8 (130.7 to 247) | 253.5 (187.6 to 326.3) |  | 10.1 (5.2 to 19.8) | 15.2 (9.7 to 24.3) |
| Federated States of Micronesia | 1.5 (1 to 2.1) | 1.8 (1.2 to 2.5) |  | 0.1 (0 to 0.1) | 0.1 (0.1 to 0.1) |
| Fiji | 12 (9.2 to 15.4) | 12.1 (8.8 to 16) |  | 0.4 (0.3 to 0.6) | 0.4 (0.3 to 0.6) |
| Finland | 21.5 (18.6 to 24.5) | 4.8 (4 to 5.6) |  | 2.4 (1.9 to 2.9) | 0.2 (0.2 to 0.3) |
| France | 95.5 (81.8 to 110.3) | 32.5 (27.3 to 38.7) |  | 10.5 (8.9 to 12.4) | 2.3 (1.9 to 2.8) |
| Gabon | 2.2 (1.4 to 3.3) | 4 (2.2 to 6.4) |  | 0.2 (0.1 to 0.4) | 0.4 (0.2 to 0.7) |
| Gambia | 1.9 (1.2 to 3) | 7.4 (4.7 to 11.4) |  | 0.6 (0.3 to 1) | 1.9 (1 to 3.3) |
| Georgia | 61.6 (57.7 to 66.2) | 14.4 (12.9 to 15.8) |  | 3 (2.1 to 4) | 1.6 (1.3 to 2) |
| Germany | 287.1 (252.3 to 323.5) | 55.4 (46.9 to 65.1) |  | 38.2 (30.9 to 46.3) | 5 (4.1 to 5.9) |
| Ghana | 51.8 (35.3 to 71.3) | 80.1 (51.5 to 120.6) |  | 15.7 (9 to 25.4) | 35.5 (21.3 to 56) |
| Greece | 38.4 (34.1 to 42.9) | 18.7 (16.4 to 21.1) |  | 4.1 (3.5 to 4.8) | 0.8 (0.6 to 0.9) |
| Greenland | 0.3 (0.2 to 0.4) | 0.1 (0 to 0.1) |  | 0 (0 to 0) | 0 (0 to 0) |
| Grenada | 0.4 (0.4 to 0.5) | 0.2 (0.2 to 0.3) |  | 0.1 (0.1 to 0.1) | 0 (0 to 0) |
| Guam | 1 (0.7 to 1.3) | 1.5 (1.2 to 1.8) |  | 0.1 (0 to 0.1) | 0 (0 to 0.1) |
| Guatemala | 59.3 (52.4 to 66.7) | 71.1 (59 to 84.5) |  | 3.4 (2.6 to 4.3) | 3.6 (2.8 to 4.5) |
| Guinea | 9 (6 to 13.1) | 31.4 (20.2 to 46) |  | 2.6 (1.5 to 4.3) | 8.3 (4.6 to 13.5) |
| Guinea-Bissau | 3.4 (2.2 to 5.1) | 8.9 (5.6 to 13.3) |  | 0.9 (0.5 to 1.5) | 2.2 (1.2 to 3.7) |
| Guyana | 5.4 (4.4 to 6.6) | 2.8 (2.1 to 3.8) |  | 0.8 (0.6 to 1) | 0.6 (0.4 to 0.8) |
| Haiti | 45.5 (30.9 to 64.5) | 83.1 (53.9 to 120.6) |  | 8 (4.5 to 13.3) | 12.4 (6.4 to 21.2) |
| Honduras | 14.8 (10.5 to 20.1) | 21.6 (10.6 to 35.5) |  | 3.8 (2.3 to 5.9) | 3.6 (1.7 to 6.5) |
| Hungary | 100.7 (89.6 to 112.4) | 15.6 (13.1 to 18.4) |  | 9.8 (8.5 to 11.4) | 1.3 (1.1 to 1.6) |
| Iceland | 0.9 (0.8 to 1) | 0.4 (0.3 to 0.5) |  | 0.1 (0 to 0.1) | 0 (0 to 0) |
| India | 7660.5 (6665.7 to 8752.4) | 11873.5 (10660.6 to 13099.1) |  | 219 (150.8 to 310.2) | 265.7 (189.8 to 430.8) |
| Indonesia | 1479.6 (1248.1 to 1742.3) | 2391.4 (1912.1 to 3141) |  | 157.4 (113 to 214.8) | 211.8 (129.6 to 307) |
| Iran | 399.6 (352.8 to 444.2) | 481 (444.6 to 520) |  | 75.1 (64.1 to 89.4) | 84.6 (75.6 to 94.4) |
| Iraq | 107.7 (79.2 to 143.7) | 164.8 (113.9 to 243.8) |  | 30.4 (20.6 to 44) | 45.8 (29.1 to 70.8) |
| Ireland | 10.3 (9.1 to 11.6) | 3.5 (3 to 4.2) |  | 0.8 (0.6 to 0.9) | 0.1 (0.1 to 0.1) |
| Israel | 12.9 (11.5 to 14.6) | 3.9 (3.3 to 4.5) |  | 0.8 (0.7 to 1) | 0.1 (0.1 to 0.2) |
| Italy | 119.7 (114.6 to 124.8) | 35.1 (33 to 37.3) |  | 13.8 (13 to 14.6) | 1.8 (1.6 to 1.9) |
| Ivory Coast | 27.3 (17.2 to 40.1) | 81.4 (50.5 to 123.7) |  | 7.9 (4.6 to 12.7) | 20.4 (10.8 to 34.8) |
| Jamaica | 3.2 (2.7 to 3.9) | 3.1 (2.2 to 4.3) |  | 1 (0.8 to 1.3) | 0.9 (0.6 to 1.3) |
| Japan | 190.1 (182.9 to 197.1) | 77 (73.9 to 80.3) |  | 24.9 (23.6 to 26.2) | 5.3 (5 to 5.6) |
| Jordan | 24.1 (18.8 to 30.4) | 42.8 (32 to 56.2) |  | 5 (3.5 to 6.9) | 8.2 (5.6 to 11.3) |
| Kazakhstan | 163.6 (138.2 to 191.7) | 49 (36.8 to 63.3) |  | 21.2 (17.9 to 24.1) | 9.9 (7.1 to 12.4) |
| Kenya | 22.5 (16.6 to 28.9) | 95.2 (70.2 to 127.5) |  | 3.5 (2.4 to 5) | 8.9 (6.1 to 12.5) |
| Kiribati | 0.9 (0.6 to 1.2) | 1.9 (1.3 to 2.7) |  | 0.1 (0 to 0.1) | 0.1 (0.1 to 0.2) |
| Kuwait | 18.1 (16 to 20.3) | 38.7 (31.8 to 47.2) |  | 1.1 (0.9 to 1.3) | 2.5 (2 to 3.2) |
| Kyrgyzstan | 39.1 (33.3 to 45.4) | 33.9 (27.5 to 40.9) |  | 5.4 (4.3 to 6.5) | 5.7 (4.5 to 7.1) |
| Laos | 53.5 (35.9 to 76) | 80.4 (53 to 116.3) |  | 4.1 (2.5 to 6.5) | 5.8 (3.4 to 9.4) |
| Latvia | 20.2 (17.1 to 23.3) | 4 (3.1 to 5.1) |  | 1.5 (1.3 to 1.8) | 0.3 (0.2 to 0.4) |
| Lebanon | 23.4 (16.8 to 32.1) | 19.7 (14.8 to 25.6) |  | 2.4 (1.5 to 3.9) | 1.9 (1.3 to 2.6) |
| Lesotho | 0.8 (0.4 to 1.2) | 4 (2.3 to 6.5) |  | 0.1 (0.1 to 0.3) | 0.7 (0.4 to 1.1) |
| Liberia | 4.6 (3.1 to 6.9) | 15 (9.6 to 22.6) |  | 1.3 (0.8 to 2.1) | 3.5 (1.9 to 6.1) |
| Libya | 37.3 (26.7 to 50.2) | 84.3 (59 to 116.5) |  | 4 (2.4 to 6.2) | 12.2 (7 to 19.2) |
| Lithuania | 29.5 (25.5 to 33.7) | 5.5 (4.6 to 6.5) |  | 1.8 (1.4 to 2.1) | 0.3 (0.2 to 0.4) |
| Luxembourg | 1.2 (1 to 1.4) | 0.3 (0.2 to 0.3) |  | 0.2 (0.2 to 0.2) | 0 (0 to 0) |
| Madagascar | 44.9 (31.3 to 62) | 135.2 (82.4 to 201) |  | 8.6 (5.3 to 13.3) | 18.5 (10.6 to 30) |
| Malawi | 25.8 (17.5 to 36.1) | 70.9 (47 to 100.7) |  | 3 (1.7 to 4.8) | 7.4 (4.2 to 12.3) |
| Malaysia | 97.3 (73.3 to 124.7) | 192.5 (147.5 to 240.3) |  | 8.7 (5.9 to 12.3) | 11.6 (7.9 to 16.4) |
| Maldives | 1.7 (1.3 to 2.4) | 3.2 (2.2 to 4.2) |  | 0.2 (0.1 to 0.3) | 0.2 (0.1 to 0.3) |
| Mali | 13.4 (8.4 to 20.3) | 39.6 (25.6 to 60.4) |  | 3.7 (1.9 to 6.7) | 9.8 (5.1 to 17.4) |
| Malta | 1.2 (1 to 1.3) | 0.4 (0.4 to 0.5) |  | 0.1 (0.1 to 0.1) | 0 (0 to 0) |
| Marshall Islands | 0.5 (0.4 to 0.7) | 1.1 (0.8 to 1.6) |  | 0 (0 to 0) | 0.1 (0 to 0.1) |
| Mauritania | 4.6 (2.9 to 6.9) | 7 (3.9 to 11.1) |  | 1.4 (0.8 to 2.5) | 1.8 (0.9 to 3.4) |
| Mauritius | 12.8 (11.3 to 14.3) | 9.4 (8.1 to 10.6) |  | 1.4 (1.2 to 1.7) | 0.8 (0.7 to 1) |
| Mexico | 256.7 (247.1 to 265.9) | 667.1 (603.5 to 735.2) |  | 39.4 (37.5 to 41.3) | 29.5 (26.1 to 33) |
| Moldova | 30.3 (27 to 33.7) | 20.3 (17.7 to 23) |  | 1.7 (1.3 to 2.1) | 0.6 (0.4 to 0.7) |
| Monaco | 0.1 (0 to 0.1) | 0 (0 to 0.1) |  | 0 (0 to 0) | 0 (0 to 0) |
| Mongolia | 11.5 (8.6 to 15.4) | 15.8 (11.8 to 20.8) |  | 0.3 (0.2 to 0.5) | 0.6 (0.3 to 0.9) |
| Montenegro | 3.6 (2.7 to 4.5) | 1.6 (1.1 to 2.1) |  | 0.1 (0.1 to 0.1) | 0 (0 to 0.1) |
| Morocco | 318 (232 to 426.7) | 246 (163.2 to 396.8) |  | 37.4 (21 to 60.8) | 39 (21.7 to 71.3) |
| Mozambique | 8.1 (5.1 to 12) | 41.6 (23.5 to 70.1) |  | 3.4 (2 to 5.4) | 13.7 (7.1 to 23.5) |
| Myanmar | 541.8 (355 to 799.1) | 366.3 (256.7 to 522.5) |  | 47.4 (29.3 to 73.9) | 38.1 (23.7 to 59) |
| Namibia | 1.9 (1.1 to 3) | 4.8 (2.4 to 8.1) |  | 0.3 (0.2 to 0.5) | 0.5 (0.3 to 1) |
| Nauru | 0.3 (0.2 to 0.4) | 0.4 (0.3 to 0.5) |  | 0 (0 to 0) | 0 (0 to 0) |
| Nepal | 125.2 (80.6 to 181.1) | 189.5 (123.3 to 276.2) |  | 5.2 (2.7 to 8.8) | 5.4 (2.6 to 10.8) |
| Netherlands | 38.5 (33.9 to 43.5) | 6.6 (5.6 to 7.6) |  | 3 (2.6 to 3.6) | 0.4 (0.4 to 0.5) |
| New Zealand | 10.1 (9.1 to 11.2) | 4.6 (4 to 5.3) |  | 0.7 (0.6 to 0.8) | 0.2 (0.2 to 0.2) |
| Nicaragua | 10.8 (8.5 to 13.8) | 20 (15.1 to 26.5) |  | 1.3 (0.9 to 1.8) | 1.4 (0.9 to 2) |
| Niger | 7 (3.8 to 11.7) | 22 (11.9 to 35.5) |  | 1.9 (0.9 to 3.8) | 5.2 (2.5 to 10) |
| Nigeria | 154 (110.7 to 209.9) | 437.7 (283.6 to 593.4) |  | 24.3 (15 to 40.7) | 64.7 (38.3 to 97) |
| Niue | 0 (0 to 0) | 0 (0 to 0) |  | 0 (0 to 0) | 0 (0 to 0) |
| North Korea | 127.2 (79.2 to 195.4) | 220.3 (142.7 to 346.8) |  | 12.5 (7.4 to 20.3) | 19.9 (11.6 to 33.3) |
| North Macedonia | 11 (8.7 to 13.6) | 4.7 (3.4 to 6.4) |  | 1.6 (1.1 to 2.1) | 0.6 (0.4 to 0.9) |
| Northern Mariana Islands | 0.4 (0.2 to 0.6) | 0.3 (0.2 to 0.4) |  | 0 (0 to 0.1) | 0 (0 to 0) |
| Norway | 10.6 (10.1 to 11.2) | 1.7 (1.6 to 1.8) |  | 0.8 (0.7 to 0.8) | 0.1 (0.1 to 0.1) |
| Oman | 14.2 (9.6 to 20.1) | 25.1 (18.2 to 33.4) |  | 1.9 (1.2 to 2.9) | 3.6 (2.4 to 5.3) |
| Pakistan | 687.2 (477.6 to 889.2) | 2741.9 (2070.1 to 3564.3) |  | 25.4 (14.1 to 39.8) | 93.6 (59 to 151.9) |
| Palau | 0.2 (0.2 to 0.3) | 0.3 (0.2 to 0.4) |  | 0 (0 to 0) | 0 (0 to 0) |
| Palestine | 12.4 (8.8 to 17.3) | 22.2 (17.1 to 28.3) |  | 2.1 (1.3 to 3.1) | 3.3 (2.3 to 4.6) |
| Panama | 5.3 (4.6 to 6.2) | 8.8 (6.9 to 11) |  | 1 (0.8 to 1.3) | 0.8 (0.6 to 1) |
| Papua New Guinea | 26.6 (14.3 to 43.1) | 85.5 (51.8 to 126) |  | 1.2 (0.6 to 2.3) | 2.9 (1.5 to 5) |
| Paraguay | 11.3 (8.6 to 14.4) | 16.1 (11.4 to 21.9) |  | 1.1 (0.8 to 1.5) | 1.1 (0.8 to 1.6) |
| Peru | 93.6 (70.4 to 122.7) | 102.6 (70.1 to 144.9) |  | 8.8 (5.9 to 12.6) | 9 (5.7 to 13.4) |
| Philippines | 774.2 (702.7 to 858.4) | 1329.5 (1127.3 to 1551) |  | 31 (27 to 35.8) | 65.5 (54.3 to 79) |
| Poland | 386.8 (375.3 to 398.3) | 40.9 (37.3 to 44.7) |  | 22.6 (21.4 to 23.8) | 6.7 (6.1 to 7.5) |
| Portugal | 27.1 (23.2 to 31.8) | 8.3 (7 to 9.8) |  | 8.1 (6.9 to 9.4) | 0.8 (0.7 to 0.9) |
| Puerto Rico | 13 (11.3 to 14.9) | 5.3 (4.2 to 6.4) |  | 0.9 (0.7 to 1.1) | 0.2 (0.2 to 0.3) |
| Qatar | 4.2 (3 to 5.5) | 11 (7.6 to 15.2) |  | 0.2 (0.1 to 0.3) | 0.7 (0.5 to 1.2) |
| Republic of Serbia | 52.6 (41.6 to 64) | 15.1 (11.2 to 19.5) |  | 7.1 (4.9 to 9.7) | 1.9 (1.2 to 2.8) |
| Republic of the Congo | 8.4 (5 to 13.2) | 19.1 (11.9 to 30.1) |  | 0.9 (0.5 to 1.5) | 2 (1.1 to 3.4) |
| Romania | 145.8 (129.7 to 164) | 51.9 (44.6 to 59.9) |  | 14 (11 to 17.4) | 4.2 (3.4 to 5.1) |
| Russia | 1436.2 (1384.3 to 1480.4) | 747.5 (693.3 to 806.5) |  | 140.5 (135.4 to 145.3) | 93 (84.7 to 100.5) |
| Rwanda | 26.5 (16.5 to 40.7) | 28 (16.9 to 44.1) |  | 4.3 (2.4 to 7.3) | 2.5 (1.3 to 4.2) |
| Saint Kitts and Nevis | 0.2 (0.2 to 0.3) | 0.1 (0 to 0.1) |  | 0.1 (0 to 0.1) | 0 (0 to 0) |
| Saint Lucia | 0.4 (0.3 to 0.4) | 0.2 (0.1 to 0.2) |  | 0.1 (0.1 to 0.1) | 0.1 (0 to 0.1) |
| Saint Vincent and the Grenadines | 0.6 (0.5 to 0.6) | 0.2 (0.2 to 0.3) |  | 0.1 (0.1 to 0.1) | 0 (0 to 0.1) |
| Samoa | 1.1 (0.7 to 1.5) | 2.1 (1.4 to 3) |  | 0.1 (0 to 0.1) | 0.1 (0.1 to 0.1) |
| San Marino | 0 (0 to 0) | 0 (0 to 0) |  | 0 (0 to 0) | 0 (0 to 0) |
| São Tomé and Principe | 0.2 (0.1 to 0.3) | 0.5 (0.3 to 0.8) |  | 0.1 (0 to 0.1) | 0.2 (0.1 to 0.3) |
| Saudi Arabia | 120.4 (82.3 to 167.1) | 525.2 (343.5 to 774.2) |  | 21 (12.9 to 31.6) | 72.5 (44.2 to 112.1) |
| Senegal | 18 (11.9 to 25.8) | 35.8 (22.9 to 53.5) |  | 5.3 (3.2 to 8.3) | 8.4 (4.6 to 14.4) |
| Seychelles | 0.6 (0.5 to 0.8) | 0.5 (0.4 to 0.6) |  | 0.1 (0 to 0.1) | 0 (0 to 0.1) |
| Sierra Leone | 9.6 (5.9 to 14.4) | 28.4 (17.6 to 42.7) |  | 2.9 (1.6 to 4.9) | 7.2 (3.8 to 12.4) |
| Singapore | 18.1 (16 to 20.3) | 10.1 (9 to 11.3) |  | 1.2 (1 to 1.4) | 0.2 (0.2 to 0.2) |
| Slovakia | 38.1 (30.4 to 46.4) | 10.9 (8.2 to 14) |  | 2.7 (1.9 to 3.8) | 0.8 (0.5 to 1.2) |
| Slovenia | 5.2 (4.5 to 6) | 0.8 (0.6 to 0.9) |  | 0.5 (0.4 to 0.6) | 0 (0 to 0.1) |
| Solomon Islands | 3.4 (1.7 to 5) | 9.4 (6.4 to 12.9) |  | 0.1 (0 to 0.1) | 0.2 (0.1 to 0.4) |
| Somalia | 16.1 (9.7 to 26.1) | 51.8 (30 to 82.6) |  | 2.1 (1 to 4) | 4 (1.9 to 7.8) |
| South Africa | 178.4 (152 to 210.5) | 164.4 (141.1 to 194.4) |  | 29.4 (24.1 to 35.9) | 24.4 (19.8 to 29.8) |
| South Korea | 118 (82.5 to 159.6) | 33.2 (24 to 44.7) |  | 26.9 (18.9 to 36.6) | 4.5 (3.1 to 6.3) |
| South Sudan | 11.4 (7 to 17.7) | 26.8 (16.2 to 41.6) |  | 1.4 (0.7 to 2.4) | 2.1 (1.1 to 3.8) |
| Spain | 105.7 (90.8 to 121.9) | 35.2 (30.3 to 40.7) |  | 12.6 (10.6 to 14.7) | 2 (1.7 to 2.4) |
| Sri Lanka | 108 (83.1 to 137.7) | 89.1 (59 to 125.5) |  | 15.6 (10.9 to 21.6) | 10.7 (6.5 to 16.2) |
| Sudan | 284.6 (190.7 to 404.8) | 471.2 (268.8 to 701.6) |  | 29.3 (14.7 to 52.8) | 62.1 (28.6 to 109.6) |
| Suriname | 2.3 (1.7 to 3.1) | 2 (1.4 to 2.7) |  | 0.2 (0.2 to 0.4) | 0.3 (0.2 to 0.4) |
| Sweden | 12.8 (11.5 to 14.4) | 2.7 (2.2 to 3.2) |  | 1.7 (1.4 to 1.9) | 0.3 (0.2 to 0.3) |
| Switzerland | 18.8 (16.3 to 21.6) | 3.4 (2.8 to 3.9) |  | 1.6 (1.3 to 1.9) | 0.1 (0.1 to 0.2) |
| Syria | 266.7 (207.6 to 330.6) | 152.3 (112 to 207.5) |  | 24.6 (16.7 to 35.4) | 13.4 (8.8 to 19.8) |
| Tajikistan | 40.4 (32.4 to 49) | 50.6 (36.8 to 65.7) |  | 5.6 (3.7 to 8.1) | 5.9 (3.6 to 8.9) |
| Thailand | 211.9 (138.2 to 296.8) | 225.9 (152.6 to 320.5) |  | 19.3 (12 to 29) | 29.8 (19 to 43.4) |
| The Bahamas | 1.4 (1.2 to 1.6) | 1 (0.8 to 1.3) |  | 0.1 (0.1 to 0.2) | 0.1 (0.1 to 0.1) |
| Togo | 7.5 (5.1 to 10.9) | 21 (12.8 to 32.2) |  | 2.2 (1.3 to 3.5) | 5.2 (2.9 to 8.5) |
| Tokelau | 0 (0 to 0) | 0 (0 to 0) |  | 0 (0 to 0) | 0 (0 to 0) |
| Tonga | 0.4 (0.3 to 0.5) | 0.6 (0.4 to 0.9) |  | 0 (0 to 0) | 0 (0 to 0) |
| Trinidad and Tobago | 7.6 (6.7 to 8.6) | 6.6 (4.9 to 8.7) |  | 0.9 (0.8 to 1.1) | 0.7 (0.5 to 0.9) |
| Tunisia | 49.8 (35.3 to 66.8) | 56.1 (36.6 to 80.1) |  | 5.6 (3.4 to 8.5) | 7.5 (4.1 to 12.2) |
| Turkey | 396.6 (287 to 524.5) | 234.9 (170.1 to 310.8) |  | 56.9 (37.6 to 82.5) | 27.5 (18.5 to 39.2) |
| Turkmenistan | 36.3 (31.4 to 41.1) | 40.9 (31.1 to 54.2) |  | 4.2 (2.9 to 5.5) | 7.4 (5 to 10.3) |
| Tuvalu | 0.1 (0.1 to 0.2) | 0.2 (0.1 to 0.3) |  | 0 (0 to 0) | 0 (0 to 0) |
| Uganda | 27.9 (16.9 to 42.5) | 107.7 (67.2 to 162) |  | 3.2 (1.7 to 5.8) | 7.7 (4.2 to 13) |
| Ukraine | 299.1 (242.8 to 363.2) | 326.7 (231.2 to 448.2) |  | 29.8 (23.1 to 37.9) | 27.1 (18.6 to 37.3) |
| United Arab Emirates | 13.7 (9 to 19.8) | 31.3 (19.4 to 44) |  | 1.8 (1 to 2.8) | 3.8 (2.2 to 5.7) |
| United Kingdom | 150.1 (146.7 to 153.3) | 58.9 (57.1 to 60.9) |  | 11.5 (11.1 to 12) | 2.4 (2.3 to 2.5) |
| United Republic of Tanzania | 61.2 (40.7 to 88.2) | 226 (140.1 to 331.4) |  | 5.2 (3.1 to 8.4) | 15.3 (8.1 to 26) |
| United States of America | 792.2 (768.7 to 816.6) | 582.6 (541.3 to 617.4) |  | 44.9 (43 to 47) | 34.7 (32.2 to 37.2) |
| United States Virgin Islands | 0.7 (0.5 to 0.9) | 0.3 (0.2 to 0.5) |  | 0 (0 to 0.1) | 0 (0 to 0) |
| Uruguay | 10.1 (8.8 to 11.5) | 4.5 (3.8 to 5.3) |  | 1.2 (1 to 1.5) | 0.4 (0.4 to 0.5) |
| Uzbekistan | 185.9 (162 to 210.7) | 306.5 (251.9 to 363.3) |  | 21.3 (18.3 to 25.1) | 25.6 (21.1 to 30.7) |
| Vanuatu | 2.3 (1.4 to 3.2) | 5.9 (4.1 to 8) |  | 0.1 (0.1 to 0.2) | 0.2 (0.1 to 0.4) |
| Venezuela | 119.6 (106.1 to 135.1) | 146.1 (109.5 to 192.2) |  | 7.6 (6.1 to 9.3) | 7.1 (4.9 to 10) |
| Vietnam | 150.9 (99.3 to 221.8) | 227.2 (145 to 350.8) |  | 30.1 (18.6 to 46.6) | 43.5 (24.2 to 72.4) |
| Yemen | 98.4 (54.5 to 158.4) | 234.1 (141.4 to 364.5) |  | 11.1 (5 to 19.9) | 36.2 (17 to 60.7) |
| Zambia | 14.2 (9.6 to 20.6) | 54.3 (32 to 83.9) |  | 2.1 (1.1 to 3.6) | 5.5 (3 to 9.4) |
| Zimbabwe | 7.8 (5.3 to 11.1) | 40 (24.6 to 61.1) |  | 1.3 (0.8 to 1.9) | 6.8 (3.9 to 11.1) |

| **Supplementary Table 10. Age standardized DALYs number of ischemic heart disease vs ischemic stroke in youths and young Adults (15-39 years) at country level, 1990-2021, both sexes** | | | | | |
| --- | --- | --- | --- | --- | --- |
|  | **Ischemic heart disease (DALYs number, 95% UI)** | |  | **Ischemic stroke (DALYs number, 95% UI)** | |
|  | **Age standardized number in 1990** | **Age standardized number in 2021** |  | **Age standardized number in 1990** | **Age standardized number in 2021** |
| Afghanistan | 5515.4 (3434.4 to 8279.8) | 18359 (12061.2 to 27504.1) |  | 915.4 (574.2 to 1400.6) | 3755.7 (2397.6 to 5985.7) |
| Albania | 711.1 (551.2 to 903.8) | 354.4 (263.4 to 471.6) |  | 132.3 (90.1 to 183.4) | 77.3 (51.5 to 110.4) |
| Algeria | 14444.5 (10364.9 to 19627.1) | 14358.3 (10029.1 to 19705.5) |  | 2443.3 (1686.4 to 3435.5) | 3406.2 (2365.5 to 4801.7) |
| American Samoa | 22.1 (15.6 to 30.8) | 25.1 (17.3 to 33.9) |  | 3.3 (2.4 to 4.4) | 2.7 (1.9 to 3.6) |
| Andorra | 6.1 (4.2 to 8.8) | 2.9 (1.9 to 4.3) |  | 1.4 (1 to 1.9) | 1 (0.7 to 1.5) |
| Angola | 1299.5 (808.4 to 1923.1) | 4110.2 (2600 to 6185.1) |  | 405.8 (283.7 to 559) | 1123 (783 to 1554.6) |
| Antigua and Barbuda | 12.2 (10.4 to 14.4) | 4.2 (3.3 to 5.1) |  | 2.3 (1.9 to 2.7) | 1.6 (1.3 to 2.1) |
| Argentina | 8252.8 (7209.3 to 9388) | 4265.8 (3602.4 to 4941.9) |  | 1070.5 (842.3 to 1355) | 866.7 (616.4 to 1187.8) |
| Armenia | 1513.3 (1312.3 to 1705.5) | 717.6 (619.7 to 822) |  | 248.7 (187.3 to 316.8) | 132.2 (97.7 to 175.8) |
| Australia | 2300.6 (1991.5 to 2638.4) | 1210.1 (1016.3 to 1423.2) |  | 410.7 (306.9 to 541.4) | 376.2 (251.4 to 524.1) |
| Austria | 1102.1 (965.8 to 1266.2) | 326.6 (278.6 to 376.7) |  | 281.5 (217.9 to 359.5) | 133.9 (86.5 to 194.1) |
| Azerbaijan | 5036 (4159.8 to 5959.6) | 3818.3 (2963 to 4765.3) |  | 447.2 (326.7 to 588.2) | 438.9 (300.8 to 597.9) |
| Bahrain | 365.9 (294.9 to 445.9) | 528 (402 to 671.5) |  | 40.7 (30.1 to 52.6) | 90.8 (66.7 to 118.1) |
| Bangladesh | 36853.6 (26214.4 to 50147.8) | 49764.1 (34370.9 to 68771) |  | 7378.2 (4694.5 to 11634.5) | 45.1 (38 to 53.1) |
| Barbados | 45.1 (38 to 53.1) | 17.4 (12.9 to 23.1) |  | 11.2 (9.2 to 13.4) | 6.5 (5.1 to 8.3) |
| Belarus | 4793.6 (4069 to 5607.6) | 3248.4 (2577.9 to 4001.2) |  | 708.5 (541.1 to 891.8) | 411.7 (305.2 to 540.6) |
| Belgium | 1607 (1403.3 to 1819.9) | 332.5 (282.2 to 385.9) |  | 341.3 (271 to 427.8) | 135.3 (92.5 to 193.4) |
| Belize | 32 (26.9 to 37.5) | 46.1 (37.8 to 55.5) |  | 4.5 (3.6 to 5.5) | 10 (7.9 to 12.6) |
| Benin | 329.5 (219 to 485.6) | 1151.7 (734.3 to 1694.2) |  | 209.8 (148.5 to 283.1) | 621.7 (441.6 to 849.4) |
| Bermuda | 16.2 (13.7 to 19) | 3.2 (2.5 to 4) |  | 2 (1.5 to 2.5) | 0.8 (0.6 to 1) |
| Bhutan | 196.5 (108.5 to 302.9) | 243.8 (143.9 to 373.1) |  | 18.3 (12.3 to 26) | 22.1 (14.7 to 31.4) |
| Bolivia | 2293 (1571.6 to 3333.6) | 2105.1 (1345.2 to 3226.2) |  | 320.9 (206 to 482.2) | 307.4 (208.7 to 424.5) |
| Bosnia and Herzegovina | 1376.6 (1100.4 to 1707) | 346.8 (236.2 to 471.2) |  | 370.9 (266.8 to 493.5) | 176.2 (127.6 to 231.2) |
| Botswana | 133.2 (65.2 to 221.4) | 267.8 (137.3 to 424.7) |  | 50.3 (32.5 to 72.4) | 93.7 (62.9 to 131.2) |
| Brazil | 42741.6 (40422.1 to 45207.2) | 42443 (39913.8 to 45228.5) |  | 7247.5 (6607.5 to 7986.4) | 5096.4 (4489.4 to 5821.3) |
| Brunei | 163.5 (122.4 to 212.7) | 162.9 (122.3 to 207.9) |  | 17.7 (12.9 to 24) | 15.4 (11 to 20.7) |
| Bulgaria | 4238.6 (3791.9 to 4715.7) | 2150.8 (1777.8 to 2557.2) |  | 632.5 (523.5 to 754.5) | 383.4 (308.1 to 470.9) |
| Burkina Faso | 548.9 (352.7 to 825.5) | 1743.7 (1073 to 2673.6) |  | 301.2 (206.8 to 424.3) | 802.6 (551.1 to 1118.9) |
| Burundi | 1283.2 (817.1 to 1943.8) | 2849.5 (1880.8 to 4059.6) |  | 328.8 (219.8 to 477.4) | 512 (352.3 to 729.4) |
| Cabo Verde | 48.9 (33.5 to 68.3) | 87.1 (55.5 to 130.5) |  | 24.4 (17.4 to 33.6) | 38.2 (26.7 to 53.2) |
| Cambodia | 3014.6 (2125.5 to 4152.6) | 4571.1 (2927.1 to 7036.2) |  | 485.2 (343.3 to 669.8) | 744.1 (519.8 to 1042) |
| Cameroon | 812.7 (508.3 to 1197.1) | 4380.1 (2616.8 to 6799.2) |  | 470.3 (325.4 to 662.8) | 1855.5 (1270.2 to 2659.7) |
| Canada | 3701.9 (3229.5 to 4217.5) | 1977.6 (1680.2 to 2308.9) |  | 956.3 (683.1 to 1312.7) | 827.3 (558.9 to 1158.3) |
| Central African Republic | 459.1 (274.2 to 736.7) | 1042.9 (587.8 to 1698.2) |  | 110.9 (75.7 to 156.2) | 212.5 (139 to 304.1) |
| Chad | 469.3 (300.4 to 705.3) | 1888.4 (1168.6 to 2793.6) |  | 291.6 (199.3 to 424.4) | 908.1 (625.6 to 1310.8) |
| Chile | 1508.3 (1288.9 to 1753.6) | 1497.2 (1255.7 to 1743.7) |  | 473.2 (376 to 600.1) | 410.3 (296.1 to 562.8) |
| China | 333875.9 (289051.5 to 382087.6) | 291780.4 (241650 to 348657.5) |  | 82040.3 (65475.7 to 101004.2) | 69993 (55513.4 to 85526.1) |
| Colombia | 8824.3 (7730.1 to 10055.2) | 6455.8 (5261.9 to 7757.9) |  | 1420 (1198.8 to 1666.7) | 944.7 (749.8 to 1174.9) |
| Comoros | 71.6 (29.2 to 116.1) | 139.7 (91.1 to 199.1) |  | 21.9 (13.5 to 32.8) | 29.9 (20.8 to 41.4) |
| Cook Islands | 7.7 (5.1 to 11.3) | 4.7 (3 to 6.9) |  | 1.2 (0.9 to 1.7) | 0.8 (0.6 to 1.1) |
| Costa Rica | 544.6 (471.3 to 620.6) | 756.9 (639.6 to 881.9) |  | 83.7 (67.4 to 105.3) | 89.6 (68.5 to 114.7) |
| Croatia | 1379.1 (1225.2 to 1544.4) | 269.1 (224.8 to 316.5) |  | 224.4 (174.3 to 285.5) | 83.3 (55.6 to 116.7) |
| Cuba | 3259.4 (2884.5 to 3672.6) | 1199.3 (981.1 to 1429.1) |  | 427.6 (350.8 to 515.8) | 209.5 (164.7 to 260.9) |
| Cyprus | 102.9 (78 to 134.4) | 90.3 (66.5 to 119.9) |  | 15.2 (10.8 to 20.6) | 17.6 (11.8 to 24.7) |
| Czechia | 2968.5 (2659.4 to 3300.2) | 729.9 (624 to 841.4) |  | 548.5 (427.8 to 695.3) | 235 (158.6 to 328.6) |
| Democratic Republic of the Congo | 4584.2 (2777.3 to 7149.4) | 10395.6 (6427.5 to 16004.9) |  | 1329.1 (918.5 to 1886.8) | 2634.6 (1750.3 to 3738.2) |
| Denmark | 806.6 (708.4 to 914.9) | 180.6 (153.6 to 209.7) |  | 150 (117.7 to 189) | 65.7 (43.7 to 92.6) |
| Djibouti | 52.9 (30.3 to 82.8) | 262.1 (147.3 to 408.7) |  | 16.1 (10.9 to 22.6) | 53.8 (36.8 to 75.8) |
| Dominica | 7.3 (5.3 to 9.7) | 5.8 (3.9 to 8.4) |  | 1.8 (1.4 to 2.4) | 1.7 (1.2 to 2.3) |
| Dominican Republic | 3003.6 (2341.6 to 3775.3) | 4985 (3555.8 to 6805) |  | 306.1 (227.6 to 406.1) | 486.3 (349.3 to 669.6) |
| East Timor | 267.6 (174.4 to 389.4) | 534.9 (335.5 to 801.2) |  | 39.1 (27.5 to 54.1) | 72.7 (46.9 to 104.8) |
| Ecuador | 3005.3 (2606.8 to 3422.2) | 4173.6 (3279.4 to 5239.7) |  | 587.7 (477.3 to 700.2) | 438.8 (342.4 to 552.9) |
| Egypt | 74796.4 (61098.2 to 90214.4) | 107564.4 (85159.1 to 132696.9) |  | 7825.8 (4539.1 to 12641.7) | 13372.7 (9375.8 to 18991.8) |
| El Salvador | 2024.5 (1617.8 to 2553.2) | 1625.1 (1172.5 to 2203.7) |  | 213.9 (159.7 to 281.3) | 152.5 (112.5 to 203.9) |
| Equatorial Guinea | 64.5 (38.5 to 101.5) | 264.6 (140.9 to 437.4) |  | 16.5 (11.1 to 23.5) | 63.2 (42.8 to 92) |
| Eritrea | 563.5 (348 to 866.1) | 1690.1 (1014.3 to 2578) |  | 131 (86.8 to 193.1) | 264.2 (179.8 to 372.8) |
| Estonia | 594.3 (494.4 to 710.7) | 66.6 (53.5 to 81.1) |  | 84.1 (64.4 to 106.3) | 31.7 (21.1 to 45.2) |
| eSwatini | 56.8 (30.9 to 88.6) | 211.3 (117 to 343.6) |  | 22.9 (15.3 to 32.1) | 48.1 (31.6 to 70) |
| Ethiopia | 10732.4 (8041.7 to 15182.2) | 15648.6 (11634.1 to 20112.8) |  | 1494.5 (1040.3 to 2149.2) | 2666.8 (1928.4 to 3524.9) |
| Federated States of Micronesia | 82.5 (54.2 to 117.3) | 101.6 (67.4 to 142.1) |  | 9.3 (6.4 to 13.2) | 9.5 (6.6 to 13.1) |
| Fiji | 689.6 (526.9 to 885.1) | 692.7 (502.5 to 916.6) |  | 64 (46.8 to 84.6) | 65.3 (46.8 to 87.2) |
| Finland | 1235.3 (1069.8 to 1406.1) | 288.2 (244.8 to 337.5) |  | 222.3 (177.5 to 271.5) | 85.3 (58 to 120.8) |
| France | 5540.1 (4754.6 to 6366.4) | 1964.8 (1665 to 2317.3) |  | 1295.1 (1011.5 to 1643.3) | 635.7 (454.6 to 863) |
| Gabon | 128.1 (80.5 to 192.4) | 233.4 (131.2 to 374.2) |  | 36.2 (25.5 to 49.1) | 66 (44.8 to 91.2) |
| Gambia | 116.5 (71.8 to 178.4) | 448.5 (284.7 to 689.1) |  | 69.5 (47.9 to 99) | 190.8 (127.1 to 275.4) |
| Georgia | 3525.3 (3296.9 to 3788.2) | 816.3 (730.7 to 897.2) |  | 417.5 (307.2 to 532.3) | 209.2 (160.2 to 263.6) |
| Germany | 16541.8 (14520 to 18632.9) | 3284.8 (2817.5 to 3852.5) |  | 3862 (3058.6 to 4792) | 1435.3 (972.5 to 2062.5) |
| Ghana | 3062.3 (2092.2 to 4213.7) | 4762.5 (3084.1 to 7138.4) |  | 1545.5 (1088 to 2164.6) | 3684.8 (2613.1 to 5033.1) |
| Greece | 2227.7 (1977.3 to 2485) | 1061.9 (930.4 to 1204.2) |  | 407.6 (331 to 494.9) | 146.1 (105.1 to 199.6) |
| Greenland | 15.9 (10.9 to 23.2) | 4 (2.6 to 5.6) |  | 3.4 (2.5 to 4.6) | 1.5 (1 to 2.1) |
| Grenada | 26.1 (22 to 30.8) | 11.9 (9.5 to 14.8) |  | 6.9 (5.6 to 8.3) | 3 (2.4 to 3.7) |
| Guam | 57.3 (41.8 to 73.3) | 83.3 (67.8 to 100.8) |  | 9.2 (6.6 to 12.2) | 8.9 (6.4 to 11.8) |
| Guatemala | 3631 (3205.2 to 4083.3) | 4334.4 (3611.2 to 5145.1) |  | 280.7 (221.1 to 344.2) | 373.3 (302.1 to 456.1) |
| Guinea | 533.7 (358.8 to 781) | 1892.3 (1221.9 to 2761.4) |  | 309.2 (214.8 to 430.7) | 876.4 (611.7 to 1242.2) |
| Guinea-Bissau | 201.9 (130.1 to 302.7) | 527.4 (336 to 790.1) |  | 89.9 (62.8 to 129.2) | 198.8 (134.1 to 294.1) |
| Guyana | 321.6 (259 to 388.1) | 166.1 (120.9 to 222) |  | 59.9 (48.3 to 73.5) | 42.8 (32.5 to 56.8) |
| Haiti | 2671.1 (1813.6 to 3794.6) | 4859 (3165.9 to 7052.1) |  | 557.5 (340.1 to 880.5) | 898 (540.5 to 1415.7) |
| Honduras | 877.9 (624.9 to 1193.8) | 1286 (653.8 to 2096.5) |  | 279.3 (188 to 407) | 325.5 (201.3 to 506.5) |
| Hungary | 5634.8 (5012.4 to 6291.9) | 897 (755.3 to 1053.6) |  | 896.7 (749.5 to 1075.1) | 271.9 (194.4 to 371.8) |
| Iceland | 53.1 (46.1 to 60.9) | 23.4 (19.7 to 27.5) |  | 7.9 (6 to 10) | 4.8 (3.2 to 6.9) |
| India | 453183.3 (393868.2 to 518305.4) | 692866.4 (621916.8 to 764804.6) |  | 26803.7 (20144.9 to 33953.8) | 40188 (29937.9 to 53027.9) |
| Indonesia | 87947.2 (74065.6 to 103714.8) | 140219.4 (112389.2 to 183652) |  | 17375.4 (13684.5 to 21667.4) | 22635.6 (16807.8 to 29156.4) |
| Iran | 24379.1 (21538.9 to 27057.7) | 28439.9 (26265.8 to 30778.4) |  | 6772.1 (5801.1 to 7922.4) | 7987.6 (6885.1 to 9125.6) |
| Iraq | 6462.9 (4774.8 to 8622.6) | 9910.7 (6886.6 to 14552.7) |  | 2510.9 (1851.6 to 3370.7) | 4290.2 (3089.9 to 5956.6) |
| Ireland | 600.4 (532.4 to 677.8) | 207.6 (175 to 244.2) |  | 95.5 (73.8 to 122.6) | 51 (33.7 to 73.7) |
| Israel | 761.2 (674.1 to 857.2) | 244.1 (209 to 283.9) |  | 120.8 (91.6 to 157.2) | 114.1 (74.9 to 164.7) |
| Italy | 7048.1 (6731.5 to 7355.6) | 2130.1 (1989 to 2273.4) |  | 1736.4 (1425 to 2103.5) | 634.7 (462.6 to 838.2) |
| Ivory Coast | 1642.2 (1043.8 to 2404) | 4852.1 (3026.9 to 7344.3) |  | 903.6 (645.8 to 1241.1) | 2131.3 (1458.3 to 3083.5) |
| Jamaica | 196.3 (163.2 to 234.3) | 186.8 (135.3 to 255.5) |  | 91.2 (71.6 to 112.4) | 85.7 (65 to 111.9) |
| Japan | 11258.4 (10819.4 to 11713.4) | 4599.5 (4384.9 to 4832.7) |  | 3595.7 (2851.2 to 4458.4) | 1913.1 (1395.4 to 2509.1) |
| Jordan | 1474.7 (1153.2 to 1855.9) | 2583.7 (1945 to 3366.5) |  | 454.5 (339.2 to 589.1) | 956.7 (720.9 to 1228.5) |
| Kazakhstan | 9358.3 (7906.8 to 10946.3) | 2799 (2119.9 to 3615.7) |  | 2182.9 (1809.9 to 2579.1) | 1347.6 (1032.9 to 1709.7) |
| Kenya | 1419.1 (1067.1 to 1798.3) | 5864.1 (4369.6 to 7808.5) |  | 723.4 (537.7 to 936.4) | 1662.8 (1247.1 to 2118.7) |
| Kiribati | 48.8 (33.9 to 67.3) | 110.8 (73 to 152.5) |  | 7.9 (5.8 to 10.4) | 14.4 (10.2 to 19.7) |
| Kuwait | 1086 (964.5 to 1217.9) | 2260.5 (1866 to 2744) |  | 148.5 (117.5 to 186.4) | 349 (266.7 to 448.1) |
| Kyrgyzstan | 2285.4 (1946.3 to 2656.2) | 1935.1 (1570.2 to 2333) |  | 497.2 (403.5 to 593.1) | 519.8 (411.8 to 647.3) |
| Laos | 3188.5 (2138.3 to 4546.3) | 4780.9 (3154.8 to 6925.2) |  | 358.6 (250.9 to 511.7) | 572.7 (398.2 to 805.8) |
| Latvia | 1139.9 (969.5 to 1316.4) | 226.3 (177 to 285.9) |  | 162.6 (128.4 to 202.8) | 54.5 (39.2 to 72.6) |
| Lebanon | 1369.2 (982.9 to 1875.6) | 1192.8 (908.3 to 1536.4) |  | 236.6 (171.9 to 339) | 335 (246.9 to 436.1) |
| Lesotho | 46.4 (26.1 to 73.4) | 231.6 (136.2 to 377.2) |  | 32.3 (20.3 to 46.4) | 73.7 (50.3 to 103.8) |
| Liberia | 276.7 (183.9 to 407.4) | 900.5 (576.7 to 1354.3) |  | 149.9 (104.5 to 203.9) | 362 (245.7 to 528.2) |
| Libya | 2290.3 (1640.4 to 3077.4) | 4990.4 (3510.1 to 6871.9) |  | 399.9 (280.1 to 549.3) | 1027.8 (698 to 1459.1) |
| Lithuania | 1668.3 (1440.3 to 1910.4) | 309.4 (259.1 to 366.8) |  | 227.3 (170.7 to 296.9) | 84.1 (57.6 to 118.4) |
| Luxembourg | 67.1 (57.4 to 78.6) | 16.6 (13.8 to 19.9) |  | 16.5 (13.4 to 20.1) | 8 (5.3 to 11.4) |
| Madagascar | 2719.9 (1903.2 to 3759.1) | 8168.6 (4985.7 to 12102.6) |  | 827 (592.5 to 1147.7) | 1864.9 (1286.3 to 2604.3) |
| Malawi | 1608.7 (1089 to 2240.6) | 4349.1 (2888.4 to 6161.6) |  | 395.5 (273.1 to 546.8) | 896.6 (616.7 to 1263.8) |
| Malaysia | 5783.4 (4365 to 7411.8) | 11167.5 (8570.3 to 13970.3) |  | 1095.8 (808.9 to 1447.9) | 1853.1 (1345.3 to 2455.6) |
| Maldives | 103.5 (75.3 to 144.1) | 183.8 (130.3 to 246.2) |  | 16.1 (11.6 to 21.9) | 30.4 (21.4 to 40.2) |
| Mali | 802.7 (508.4 to 1208.9) | 2427.6 (1582.4 to 3681.1) |  | 431.9 (288.4 to 641.5) | 1156.2 (779.1 to 1681.7) |
| Malta | 68.2 (60 to 76.2) | 25.1 (21.6 to 29) |  | 12.3 (9.8 to 15.2) | 5.3 (3.7 to 7.4) |
| Marshall Islands | 29.4 (20.4 to 41.7) | 64.3 (44.8 to 89.1) |  | 3.3 (2.3 to 4.6) | 5.3 (3.7 to 7.6) |
| Mauritania | 276.3 (175.2 to 408.7) | 422.6 (242.6 to 667.5) |  | 156.2 (106.2 to 227.3) | 231.7 (153.9 to 346) |
| Mauritius | 737.1 (650.4 to 824.5) | 546.1 (472.5 to 617) |  | 128.2 (105.6 to 153.9) | 78.6 (63.4 to 95.7) |
| Mexico | 15485.1 (14897.4 to 16054.8) | 39805.4 (36174.6 to 43837.7) |  | 3764.3 (3313.3 to 4270.6) | 3371.2 (2818.8 to 3992.8) |
| Moldova | 1741.1 (1550.3 to 1939.9) | 1147.1 (1003.3 to 1299.1) |  | 226.2 (169.6 to 293.4) | 129 (92.3 to 176.6) |
| Monaco | 3.4 (2.3 to 4.8) | 2 (1.1 to 3.2) |  | 1 (0.7 to 1.3) | 0.6 (0.4 to 0.9) |
| Mongolia | 677.2 (502.2 to 906.1) | 899.4 (674.9 to 1182.2) |  | 108.6 (74.2 to 148.7) | 166.9 (117.1 to 225.4) |
| Montenegro | 206.4 (158.9 to 260.6) | 90.9 (66.9 to 118.9) |  | 20.9 (14.4 to 29.2) | 15.6 (10.4 to 21.8) |
| Morocco | 19019 (13855.7 to 25591.1) | 14563.9 (9741.7 to 23324.6) |  | 3119.4 (2039.1 to 4618.5) | 3485.4 (2318.7 to 5383.9) |
| Mozambique | 503.8 (325.1 to 741.7) | 2523.5 (1449.2 to 4199.3) |  | 472.6 (331.5 to 648.6) | 1496.1 (987 to 2149.4) |
| Myanmar | 32552.6 (21270.7 to 48080.3) | 21859.8 (15310.7 to 31118.3) |  | 4153 (2963.5 to 5855.3) | 3897.6 (2770.9 to 5366.4) |
| Namibia | 115.8 (64.9 to 176.1) | 279.9 (145.7 to 470.1) |  | 48.1 (32.2 to 66.5) | 81.4 (51.8 to 116.8) |
| Nauru | 15.6 (10.7 to 21.8) | 21.6 (15.1 to 30.8) |  | 1.7 (1.1 to 2.4) | 2 (1.4 to 2.9) |
| Nepal | 7453.4 (4815.2 to 10809.2) | 11334.6 (7407.1 to 16524.6) |  | 576.5 (377.2 to 833.3) | 783.3 (511.6 to 1162.7) |
| Netherlands | 2213.9 (1945.8 to 2496.4) | 403.1 (346.5 to 465.6) |  | 476.9 (351.8 to 629.7) | 227.6 (148.1 to 324.7) |
| New Zealand | 580.6 (520 to 644.9) | 268.1 (234.3 to 306.2) |  | 104.4 (79.9 to 136.3) | 89 (60.1 to 125.3) |
| Nicaragua | 659.1 (519.8 to 834.7) | 1196.9 (905 to 1569.8) |  | 120.3 (91.2 to 154.3) | 151.2 (111.3 to 196.3) |
| Niger | 429.6 (241.3 to 707.5) | 1362.8 (748.8 to 2176.3) |  | 317.3 (214.3 to 460.7) | 860.5 (578.5 to 1243.2) |
| Nigeria | 9213.5 (6646.3 to 12518) | 26498.3 (17162.1 to 35827.4) |  | 3841.1 (2898.8 to 5090.1) | 10026.7 (7644.6 to 12755.2) |
| Niue | 1.3 (0.8 to 2) | 1.1 (0.7 to 1.6) |  | 0.2 (0.1 to 0.2) | 0.1 (0.1 to 0.2) |
| North Korea | 7656.5 (4779.3 to 11744) | 12908.9 (8393.9 to 20253.1) |  | 1532.6 (1090.6 to 2086.2) | 2068.4 (1455.4 to 2994.3) |
| North Macedonia | 629.5 (499.7 to 782.5) | 272.5 (199.9 to 366) |  | 165.4 (124.5 to 210.9) | 100.6 (71.7 to 136.6) |
| Northern Mariana Islands | 21.7 (13.4 to 31.6) | 16.1 (11.4 to 22.4) |  | 4 (2.8 to 5.5) | 2.1 (1.5 to 2.9) |
| Norway | 612.1 (582.1 to 646.4) | 106.5 (98.4 to 115.7) |  | 125.5 (98 to 158.6) | 72.1 (49.3 to 100) |
| Oman | 842.9 (571.2 to 1186.2) | 1484.5 (1088.8 to 1961) |  | 187 (133.2 to 251.2) | 478.7 (358.5 to 618.4) |
| Pakistan | 40960.3 (28362.5 to 53119.3) | 162930.5 (123210 to 211804.9) |  | 3604.6 (2506 to 4853.7) | 11010.8 (7993.9 to 15029.7) |
| Palau | 13.6 (9 to 19.1) | 17.9 (13.1 to 23.7) |  | 1.8 (1.3 to 2.5) | 2 (1.4 to 2.7) |
| Palestine | 765.8 (542.2 to 1060.6) | 1361.3 (1051.8 to 1727.7) |  | 183 (131 to 248.7) | 349.3 (265.3 to 450.4) |
| Panama | 325.1 (280.4 to 373.5) | 529.9 (421 to 657.2) |  | 92.6 (75.6 to 110.7) | 88.8 (70.1 to 111.2) |
| Papua New Guinea | 1531.2 (820.4 to 2497.2) | 4932.7 (2985.6 to 7273.6) |  | 187.6 (122.5 to 275.8) | 456.2 (309.7 to 645.1) |
| Paraguay | 669.4 (514.2 to 849.9) | 966 (694.5 to 1302.6) |  | 99.9 (77.1 to 128.5) | 128.5 (96.4 to 168.6) |
| Peru | 5800 (4370.5 to 7574.7) | 6335.8 (4390.3 to 8903.7) |  | 783.3 (575.8 to 1036.9) | 940 (680.4 to 1255.7) |
| Philippines | 45987.6 (41744.5 to 51005.1) | 78618.3 (66732.4 to 91804.2) |  | 3597.6 (2951.8 to 4314.3) | 7388.1 (5972.3 to 8969.2) |
| Poland | 21624.1 (20978 to 22264) | 2397.4 (2181.1 to 2616.8) |  | 2238.2 (1897.6 to 2628.1) | 1066.3 (829.8 to 1329.2) |
| Portugal | 1571.1 (1346.5 to 1838) | 487.7 (412.2 to 569.6) |  | 623.4 (532.5 to 730.6) | 129.7 (98.1 to 169.1) |
| Puerto Rico | 765.1 (663.9 to 873.6) | 309.1 (250.3 to 375.5) |  | 92.5 (74.9 to 114) | 41.1 (29.8 to 54.5) |
| Qatar | 246.8 (179 to 326.1) | 666.9 (471.4 to 915.7) |  | 36.5 (25.9 to 48.6) | 185.8 (127.4 to 255) |
| Republic of Serbia | 3003.3 (2377.3 to 3652.3) | 876.6 (652.5 to 1123.7) |  | 711.4 (534.6 to 916.7) | 316.8 (219.4 to 436.3) |
| Republic of the Congo | 496.6 (295.8 to 773.2) | 1120.7 (703.1 to 1750.6) |  | 112.3 (76.6 to 157.6) | 248.7 (170.2 to 345.6) |
| Romania | 8289.9 (7387.2 to 9340.1) | 2980 (2567.2 to 3429) |  | 1466.9 (1133 to 1833.5) | 606.7 (456.7 to 790.1) |
| Russia | 80507.8 (77599.8 to 83019.7) | 41829.7 (38798.3 to 45173.6) |  | 13620.4 (11700.3 to 15786.1) | 9267.8 (7961 to 10796.1) |
| Rwanda | 1612.1 (1009.7 to 2468.2) | 1708.9 (1042.5 to 2688.1) |  | 424.8 (287 to 616.5) | 417.1 (282.2 to 589.3) |
| Saint Kitts and Nevis | 14 (12 to 16.2) | 3.6 (2.3 to 5.2) |  | 4 (3.2 to 5) | 1.6 (1.2 to 2.1) |
| Saint Lucia | 20.6 (17.5 to 24.2) | 10.9 (8.5 to 13.6) |  | 7.9 (6.5 to 9.4) | 5 (4 to 6.2) |
| Saint Vincent and the Grenadines | 33.2 (28.5 to 38) | 14.3 (11.6 to 17.4) |  | 5.9 (4.9 to 7.1) | 3.6 (2.8 to 4.3) |
| Samoa | 60.9 (39.8 to 89.6) | 122 (79.6 to 175.3) |  | 10.1 (7 to 13.7) | 13.5 (9.6 to 18.4) |
| San Marino | 1.4 (1 to 1.9) | 0.5 (0.3 to 0.8) |  | 0.6 (0.4 to 0.8) | 0.4 (0.3 to 0.5) |
| São Tomé and Principe | 10.3 (6.2 to 15.5) | 30.8 (17.5 to 49.7) |  | 7.2 (5 to 10) | 18.8 (12.5 to 27.5) |
| Saudi Arabia | 7160.3 (4910.7 to 9925.3) | 29966.5 (19696.2 to 44036.8) |  | 1714 (1189.1 to 2382.3) | 5575.6 (3774.5 to 7950.2) |
| Senegal | 1090.1 (722.7 to 1559.6) | 2171.8 (1404.3 to 3233.7) |  | 571.5 (409 to 785.6) | 1021.6 (708.8 to 1419.4) |
| Seychelles | 35.5 (27.8 to 44.3) | 27.2 (21.3 to 34.3) |  | 6 (4.4 to 8) | 5.7 (4 to 7.6) |
| Sierra Leone | 570.9 (355.4 to 859.4) | 1710.2 (1060.1 to 2568) |  | 320.8 (225.5 to 449.5) | 739.7 (490.3 to 1072.8) |
| Singapore | 1080.3 (956.6 to 1213) | 590.1 (523.5 to 659.2) |  | 149.2 (115.7 to 191.4) | 101.9 (66 to 147) |
| Slovakia | 2164.2 (1729.3 to 2632.5) | 635.1 (484.5 to 811.4) |  | 315.2 (227.6 to 411.1) | 174.7 (121.9 to 237.7) |
| Slovenia | 301.4 (260.8 to 345) | 48.5 (39.7 to 58.3) |  | 75 (54.5 to 99.8) | 29.7 (18.8 to 43.8) |
| Solomon Islands | 197.7 (101.5 to 289.2) | 538.7 (367.2 to 745.1) |  | 17.6 (11.9 to 24.8) | 41.1 (28.2 to 56.9) |
| Somalia | 972.6 (591.8 to 1563) | 3189.5 (1878.7 to 5033.1) |  | 284.8 (183 to 422.7) | 667.1 (432.7 to 975.3) |
| South Africa | 10278.8 (8773.7 to 12110.5) | 9465 (8135.7 to 11147.6) |  | 2906.8 (2403.2 to 3519.6) | 2841.9 (2304.3 to 3468.4) |
| South Korea | 7081.7 (4995.7 to 9551.9) | 1998.2 (1477.3 to 2634.8) |  | 3028.4 (2248.1 to 3901.4) | 1157.7 (770 to 1600.6) |
| South Sudan | 702.8 (432.9 to 1083) | 1662.3 (1015 to 2571.9) |  | 199.4 (132.4 to 281.1) | 303.3 (201.6 to 434.7) |
| Spain | 6216.5 (5341.8 to 7159.5) | 2052.3 (1759.3 to 2359) |  | 1291 (1048.9 to 1591) | 477.8 (342.1 to 664.4) |
| Sri Lanka | 6417.7 (4951.3 to 8182.8) | 5249.4 (3500.1 to 7365.5) |  | 1543.9 (1173.3 to 1987.7) | 1231.2 (878.7 to 1679.8) |
| Sudan | 17042.7 (11369.3 to 24313.6) | 28271.7 (16287.9 to 42058.4) |  | 2371.4 (1411.3 to 3792.7) | 5255.7 (3098.8 to 8191.4) |
| Suriname | 137.9 (97.2 to 181) | 115.6 (81.8 to 158.5) |  | 20.1 (14.2 to 27) | 22 (16.1 to 29.5) |
| Sweden | 752.2 (671 to 841.6) | 175.6 (146.4 to 209.8) |  | 221.6 (171.4 to 282.3) | 197.1 (128.2 to 286.8) |
| Switzerland | 1104.9 (955.6 to 1265.2) | 207.9 (177.9 to 243.1) |  | 185.5 (145.6 to 237.3) | 85.7 (57.8 to 120.3) |
| Syria | 16420.3 (12790.4 to 20339.8) | 9225.3 (6801.6 to 12539) |  | 2072.8 (1543.2 to 2766.1) | 1258.5 (919.7 to 1687.2) |
| Tajikistan | 2386.4 (1913 to 2893.5) | 2953.9 (2159.1 to 3835.4) |  | 487.7 (357.7 to 654.9) | 643.5 (452.2 to 870.8) |
| Thailand | 12713 (8372.7 to 17774.3) | 13094.1 (8915.3 to 18536.6) |  | 2935 (2058.8 to 3966.4) | 3187.7 (2267.7 to 4256.3) |
| The Bahamas | 81.4 (68.9 to 95) | 58.1 (44.1 to 76.4) |  | 11.1 (8.9 to 13.7) | 11.1 (8.6 to 14) |
| Togo | 453.4 (304.7 to 653.2) | 1254.8 (769.6 to 1919.4) |  | 250.7 (181.2 to 336.7) | 567.9 (391.3 to 799.6) |
| Tokelau | 0.7 (0.5 to 1.1) | 0.9 (0.7 to 1.3) |  | 0.1 (0.1 to 0.1) | 0.1 (0.1 to 0.1) |
| Tonga | 22.9 (16.4 to 30.6) | 34.3 (22.9 to 52) |  | 4.2 (2.9 to 5.9) | 4.7 (3.3 to 6.5) |
| Trinidad and Tobago | 444.5 (389.7 to 505.6) | 375.5 (282.7 to 495.4) |  | 77.9 (64.6 to 93.3) | 57.9 (44.8 to 72.4) |
| Tunisia | 2993.6 (2121.7 to 4012.4) | 3288.3 (2153.4 to 4669.4) |  | 549.5 (382.7 to 750.8) | 739.5 (498.3 to 1043.1) |
| Turkey | 23470.7 (17028.1 to 31079.9) | 14125.6 (10354.2 to 18647.5) |  | 5684.3 (4191.1 to 7480.4) | 4007.7 (2937 to 5239.2) |
| Turkmenistan | 2111.7 (1829 to 2393.8) | 2379.3 (1810.2 to 3143.1) |  | 393.5 (299 to 493) | 698.8 (526.1 to 893) |
| Tuvalu | 7.2 (5 to 9.8) | 11.9 (8.4 to 16) |  | 0.8 (0.6 to 1.1) | 1 (0.7 to 1.4) |
| Uganda | 1725.9 (1051.8 to 2618.7) | 6679.6 (4176 to 10010) |  | 570.2 (385.9 to 797.5) | 1354.2 (906.3 to 1899.5) |
| Ukraine | 16983.9 (13833.2 to 20597) | 18392.8 (13028.7 to 25145.4) |  | 3608.8 (2739.1 to 4631.8) | 2965.4 (2190.9 to 3883.6) |
| United Arab Emirates | 816.6 (539.7 to 1173.5) | 1862.5 (1189.4 to 2606.3) |  | 220.4 (159.8 to 299.3) | 667.3 (487.2 to 900.6) |
| United Kingdom | 8593.4 (8382.4 to 8809.6) | 3422.6 (3296.3 to 3570.1) |  | 1626.9 (1326.5 to 1967) | 894.8 (657.7 to 1163.1) |
| United Republic of Tanzania | 3781.8 (2527.7 to 5433.9) | 13851.7 (8664.4 to 20261.4) |  | 823.9 (573.5 to 1127.1) | 2366.3 (1591.3 to 3278.3) |
| United States of America | 45235.1 (43868.5 to 46737.2) | 33776.5 (31405.7 to 35792.9) |  | 11345.5 (8575.3 to 14497.1) | 10748.4 (8122.2 to 13739.9) |
| United States Virgin Islands | 37.8 (27.1 to 51.1) | 17.7 (10.4 to 26.8) |  | 3.2 (2.3 to 4.4) | 1.5 (1 to 2.3) |
| Uruguay | 593.3 (514.5 to 679) | 269.6 (226.9 to 317.9) |  | 135.4 (107.5 to 168.8) | 72.8 (53.8 to 96.1) |
| Uzbekistan | 10902.6 (9485.6 to 12394) | 17898.2 (14731.1 to 21251.3) |  | 1978.5 (1638.2 to 2367.7) | 2712.8 (2175.9 to 3333.3) |
| Vanuatu | 129.4 (82.7 to 184.1) | 339.4 (233.3 to 457.4) |  | 13.9 (9.7 to 18.9) | 30.5 (21.8 to 41.2) |
| Venezuela | 7091.1 (6291 to 8007.4) | 8523.8 (6413.5 to 11174.7) |  | 691.3 (562.2 to 838.2) | 674.6 (509.9 to 877.9) |
| Vietnam | 8921.8 (5893.7 to 13069.9) | 13268.8 (8582.9 to 20283) |  | 3604.7 (2536.4 to 4921.7) | 5246.3 (3666.2 to 7336.7) |
| Yemen | 5692.6 (3130.7 to 9235.1) | 13640.5 (8207.4 to 21353.4) |  | 947.7 (563.9 to 1473.6) | 2983.6 (1790.5 to 4482.4) |
| Zambia | 882.7 (598.5 to 1272.7) | 3286.3 (1947 to 5064.2) |  | 273.3 (182 to 389.2) | 719.4 (484.1 to 1010.7) |
| Zimbabwe | 482.3 (333.5 to 682.4) | 2389.1 (1480.9 to 3639) |  | 274.5 (186.8 to 378.1) | 753.5 (514.6 to 1059.5) |
